# Ectopic expression of Cripto-1 in transgenic mouse embryos causes hemorrhages, fatal cardiac defects and embryonic lethality

**DOI:** 10.1038/srep34501

**Published:** 2016-09-30

**Authors:** Xiaolin Lin, Wentao Zhao, Junshuang Jia, Taoyan Lin, Gaofang Xiao, Shengchun Wang, Xia Lin, Yu Liu, Li Chen, Yujuan Qin, Jing Li, Tingting Zhang, Weichao Hao, Bangzhu Chen, Raoying Xie, Yushuang Cheng, Kang Xu, Kaitai Yao, Wenhua Huang, Dong Xiao, Yan Sun

**Affiliations:** 1 Institute of Comparative Medicine & Laboratory Animal Center, Southern Medical University, Guangzhou 510515, China; 2 Guangdong Provincial Key Laboratory of Cancer Immunotherapy Research and Guangzhou Key Laboratory of Tumor Immunology Research, Cancer Research Institute, Southern Medical University, Guangzhou 510515, China; 3 Department of Anatomy, Guangdong Provincial Key Laboratory of Construction and Detection in Tissue Engineering, School of Basic Medical Science, Southern Medical University, Guangzhou 510515, China; 4 Department of Medical Oncology, The Third Affiliated Hospital of Kunming Medical University (Tumor Hospital of Yunnan Province), Kunming 650118, China; 5 Department of Endocrinology, The Second Affiliated Hospital, Guangzhou Medical University, Guangzhou 510260, China; 6 Department of General Surgery, Sun Yat-sen Memorial Hospital of Sun Yat-sen University, Guangzhou 510120, China; 7 Zhongshan School of Medicine, Sun Yat-sen University, Guangzhou 510080, China

## Abstract

Targeted disruption of Cripto-1 in mice caused embryonic lethality at E7.5, whereas we unexpectedly found that ectopic Cripto-1 expression in mouse embryos also led to embryonic lethality, which prompted us to characterize the causes and mechanisms underlying embryonic death due to ectopic Cripto-1 expression. RCLG/EIIa-Cre embryos displayed complex phenotypes between embryonic day 14.5 (E14.5) and E17.5, including fatal hemorrhages (E14.5-E15.5), embryo resorption (E14.5-E17.5), pale body surface (E14.5-E16.5) and no abnormal appearance (E14.5-E16.5). Macroscopic and histological examination revealed that ectopic expression of Cripto-1 transgene in RCLG/EIIa-Cre embryos resulted in lethal cardiac defects, as evidenced by cardiac malformations, myocardial thinning, failed assembly of striated myofibrils and lack of heartbeat. In addition, Cripto-1 transgene activation beginning after E8.5 also caused the aforementioned lethal cardiac defects in mouse embryos. Furthermore, ectopic Cripto-1 expression in embryonic hearts reduced the expression of cardiac transcription factors, which is at least partially responsible for the aforementioned lethal cardiac defects. Our results suggest that hemorrhages and cardiac abnormalities are two important lethal factors in Cripto-1 transgenic mice. Taken together, these findings are the first to demonstrate that sustained Cripto-1 transgene expression after E11.5 causes fatal hemorrhages and lethal cardiac defects, leading to embryonic death at E14.5-17.5.

The inappropriate activation of embryonic genes such as Oct4 contributes to oncogenesis in somatic tissues^1^,^2^,^3^. Cripto-1 gene is a key player in this complex scenario^4^,^5^,^6^,^7^,^8^,^9^,^10^,^11^,^12^. Cripto-1 is a multifunctional modulator involved in embryogenesis and oncogenesis^4^,^5^,^6^,^7^,^8^,^9^,^10^,^11^,^12^. Previous profiling experiments have demonstrated that Cripto-1 is frequently up-regulated in many types of cancer^4^,^5^,^6^,^7^,^8^. The overexpression of Cripto-1 transgene in transgenic mouse mammary gland caused mammary hyperplasia and adenocarcinoma^13^,^14^, suggesting that Cripto-1 functions as an oncogene *in vivo*. However, a direct evidence that Cripto-1 as oncogene can also initiate other tumors is not obtained experimentally in transgenic mice because the suitable transgenic mice overexpressing Cripto-1 transgene are unavailable.

To understand the roles and mechanisms of Cripto-1 in embryogenesis and oncogenesis, Cripto-1 transgenic mice, designated as RCLG transgenic mice, were generated in this study. Both Cripto-1 and luciferase (Luc) transgenes can be simultaneously “switched-on” in a Cre-dependent manner in the same cells, tissues and organs of these mice.

Unexpectedly, our preliminary results failed to detect RCLG/EIIa-Cre genotype in 68 newborn progeny derived from matings between heterozygous RCLG transgenic mice and homozygous EIIa-Cre mice (Supplementary Figure S1 and Table S3), suggesting that the ubiquitous expression of Cripto-1 transgene may result in embryonic lethality. Studies have suggested that Cripto-1 is critically important for early embryonic^4^,^5^,^6^,^7^,^8^ and myocardial development^9^,^15^,^16^,^17^, and the targeted disruption of Cripto-1 in mice caused embryonic lethality primarily due to failures in post gastrulation morphogenesis and the impaired orientation of the anterior-posterior axis^9^,^10^,^18^. Furthermore, gain-of-function experiments in chick embryos have demonstrated that the addition of soluble Cripto to chick tissues suppressed posterior mesodermal fates and promoted anterior mesendodermal fates^11^. Based on these observations, the present study aimed to confirm the existence of embryonic lethality due to ectopic Cripto-1 expression, and further define the causes and mechanisms underlying embryonic death.

## Results

### The sustained overexpression of Cripto-1 in transgenic mice causes embryonic lethality

To further determine whether or not Cripto-1 can initiate the mentioned-above tumors, heterozygous RCLG transgenic mice were first crossed to homozygous EIIa-Cre mice to generate RCLG/EIIa-Cre double transgenic mice, in which the Cripto-1 and Luc transgenes are simultaneously activated in a diffuse pattern. Additionally, because the efficiency of Cre-mediated recombination in RCLG/EIIa-Cre transgenic mice did not reach 100%, some cells in the RCLG/EIIa-Cre transgenic mice still harbored the mRFP gene. Therefore, mRFP and Luc expression were simultaneously detectable in the RCLG/EIIa-Cre transgenic mice through the use of whole-animal fluorescence and bioluminescence imaging.

**Figure 1:**
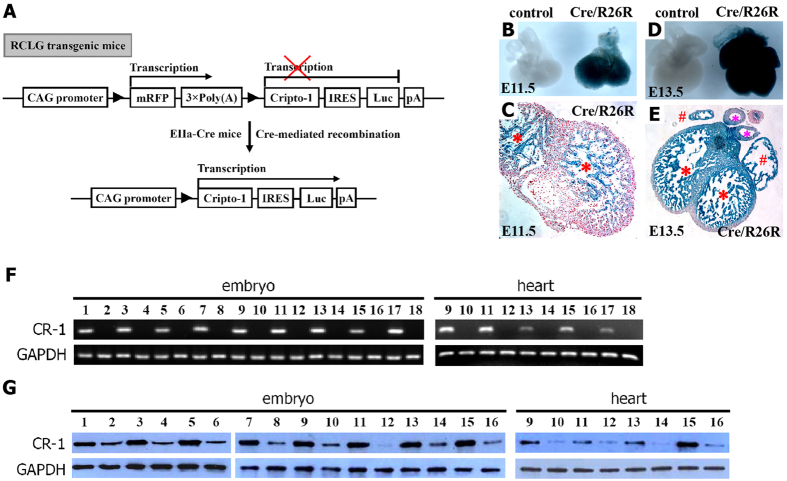
Cripto-1 (CR-1) transgene activation in embryos upon Cre-mediated excision by EIIa-Cre mice. (**A**) The strategy for the conditional expression of Cripto-1 and Luc transgenes. Please see the Materials and Methods for additional details on the strategy for the conditional expression of the transgenes. (**B–E**) Cre-mediated excision efficiency in embryonic hearts. (**B,D**) Whole-mount blue-stained embryonic hearts. (**C,E**) Histological sections of E11.5 (**C**) and E13.5 (**E**) embryonic hearts. The ventricular surface is indicated with a “*”, the atrial appendage is indicated with a “#”, and arteries are indicated with a “*”. (**F**) RT-PCR analysis using human-specific primer for human Cripto-1 transgene expression in RCLG/EIIa-Cre transgenic embryos and embryonic hearts. The cropped gels are used in Fig. 1F, and the full-length gel images are available in Supplementary Figure S28. The gels have been run under the same experimental conditions. (**G**) Western blot for the Cripto-1 protein in RCLG/EIIa-Cre transgenic embryos and their hearts. For (**F**,**G**), lane 1: E7.5+RCLG/Cre; lane 2: E7.5+Cre; lane 3: E8.5+RCLG/Cre; lane 4: E8.5+Cre; lane 5: E9.5+RCLG/Cre; lane 6: E9.5+Cre; lane 7: E10.5+RCLG/Cre; lane 8: E10.5+Cre; lane 9: E11.5+RCLG/Cre; lane 10: E11.5+Cre; lane 11: E12.5+RCLG/Cre; lane 12: E12.5+Cre; lane 13: E13.5+RCLG/Cre; lane 14: E13.5+Cre; lane 15: E14.5+RCLG/Cre; lane 16: E14.5+Cre; lane 17: E15.5+RCLG/Cre; lane 18: E15.5+Cre. The cropped blots are used in Fig. 1G, and the quantification of Cripto-1 expression and the full-length gel images are available in Supplementary Figures S6 and S28, respectively. The blots have been run under the same experimental conditions.

Unexpectedly, we failed to identify any Luc- and mRFP-positive mice from the 68 newborn progeny derived from mating heterozygous RCLG mice with homozygous EIIa-Cre mice, despite using *in vivo* fluorescence and bioluminescence imaging (Supplementary Figure S1A,B and Table S3) and PCR analysis (Supplementary Figure S1C). However, Luc- and mRFP-positive offspring were observed in the newborn progeny derived from mating RLG transgenic mice with homozygous EIIa-Cre mice (Supplementary Figure S2 and Table S3), suggesting that the both Cre/*lox* P system and the *in vivo* imaging system were functioning properly. Furthermore, both the Cripto-1 and Luc transgenes can be simultaneously “switched-on” in RCLG/EIIa-Cre transgenic mice (Fig. 1A). Based on the above findings, we hypothesized that the sustained expression of the Cripto-1 transgene during embryonic development can cause embryonic lethality.

### Time of embryonic lethality as determined by *in vivo* bioluminescence imaging

As described above, both the Cripto-1 and Luc transgenes are under the control of a CAG promoter and can be simultaneously activated in RCLG/EIIa-Cre transgenic mice (Fig. 1A). Thus, we first intended to determine whether the sustained expression of the Cripto-1 transgene led to embryonic lethality, and further sought to define the time of embryonic lethality by detecting Luc-positive embryos using *in vivo* bioluminescence imaging. To achieve these goals, heterozygous or homozygous RCLG mice were crossed with homozygous EIIa-Cre mice, and whole-body bioluminescence imaging was used to identify Luc-positive embryos at the indicated time points (Supplementary Figure S3A–D).

In general, we found that the intensity of the Luc signal gradually increased from embryonic day 11.5 (E11.5) to E13.5 (Supplementary Figure S3A–D), after which the Luc signal gradually weakened (Supplementary Figure S3A–D) before completely disappearing at E16.5 and E17.5 (Supplementary Figure S3A), indicating that the Luc-positive embryos may die or be lost between E14.5 and E17.5.

After the *in vivo* bioluminescence imaging of pregnant females, the embryos were immediately harvested at the indicated time points and subsequently imaged using the IVIS system (Supplementary Figure S4A–D) and a stereo fluorescence microscope (Supplementary Figure S3E–H). As shown in Supplementary Figure S4, one live embryo (Supplementary Figures S3F and S4A) and one live Genotype-2 embryo (Supplementary Figures S3G and S4C) emitted strong Luc signals, whereas the dead or resorbed Genotype-2 embryos showed very weak or no Luc signals (Supplementary Figure S4A,C). Furthermore, the live, dead or resorbed Genotype-2 embryos emitted red fluorescence (Supplementary Figure S4B,D). The control Genotype-1 embryos did not exhibit the Luc or mRFP signals (Supplementary Figure S4).

As shown in Supplementary Figure S3E, pregnant female 693 did not have any embryos in her uterus at E17.5, while pregnant female 1343 exhibited six resorbed embryos and one live embryo in her uterus at E17.5 (Supplementary Figure S3F). Pregnant female 1267 had five resorbed Genotype-2 embryos and one live Genotype-2 embryo in her uterus at this time (Supplementary Figure S3G), and pregnant female 1274 exhibited five dead or resorbed Genotype-2 fetuses in her uterus (Supplementary Figure S3H). All of the Genotype-1 embryos were normal (Supplementary Figure S3G,H).

In summary, these findings point to embryonic lethality and indicate that the time of embryonic death is between E14.5 and E17.5.

### EIIa-Cre-mediated Cripto-1 transgene activation in embryos

After generating RCLG mice for the conditional overexpression of Cripto-1 transgene (Fig. 1A), we subsequently crossed EIIa-Cre mice with RCLG mice to produce RCLG/EIIa-Cre transgenic mice, in which Cripto-1 and Luc transgenes can be simultaneously activated (Fig. 1A).

Data from Jackson Laboratory show that Lac Z expression is efficiently activated in E10.5 embryos and embryonic hearts of EIIa-Cre/R26R mice (data not shown). We also investigated EIIa-Cre-mediated recombination efficiency in embryonic hearts at E11.5 and E13.5 by crossing EIIa-Cre mice to R26R reporter mice (Fig. 1B–E). Embryonic hearts heterozygous for both EIIa-Cre and R26R alleles displayed ubiquitous blue staining (Fig. 1B,D). Sections of EIIa-Cre/R26R embryonic hearts at E11.5 and E13.5 illustrated a high level of X-Gal staining (Fig. 1C,E), while Lac Z staining increased considerably throughout embryonic heart at E13.5 (Fig. 1E). Together, EIIa-Cre-mediated recombination is highly efficient in embryos and embryonic heart.

As shown in Supplementary Figure S5, RCLG transgenic embryos and their hearts exhibited ubiquitous and strong mRFP transgene expression from E10.5 to E16.5, indicating the ubiquitous activity of CAG promoter (Fig. 1A) in embryos and embryonic hearts. As described above, both Cripto-1 and Luc transgenes are under the control of CAG promoter in RCLG/EIIa-Cre transgenic embryos and are simultaneously activated (Fig. 1A). Based on these results, we concluded that RCLG/EIIa-Cre embryos do express Cripto-1 after EIIa-Cre-mediated excision.

We subsequently detected human Cripto-1 expression at mRNA and protein levels in RCLG/EIIa-Cre embryos and embryonic hearts (Fig. 1F,G). After performing RT-PCR using primers specific for human Cripto-1, we only detected mRNA in RCLG/EIIa-Cre embryos and embryonic hearts (Fig. 1F). Western blot analysis revealed the significant enhanced protein expression of Cripto-1 in RCLG/EIIa-Cre embryos and embryonic hearts, compared with controls (Fig. 1G and Supplementary Figure S6). Together, these data indicate that RCLG/EIIa-Cre mice efficiently activated Cripto-1 transgene.

### EIIa-Cre-mediated Cripto-1 activation leads to hemorrhaging and embryonic death

**Figure 2:**
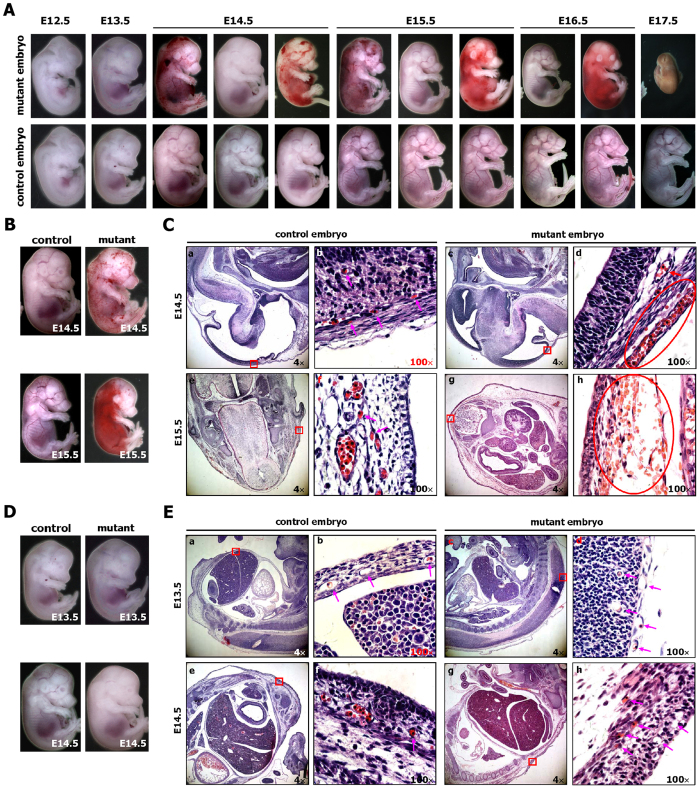
The EIIa-Cre-mediated activation of Cripto-1 leads to hemorrhaging and embryonic lethality. (**A**) Whole-mount views of representative control (EIIa-Cre) and mutant (RCLG/EIIa-Cre) embryos at E12.5 to E17.5. (**B,C**) Histological analysis of control (a,b,e,f) and mutant (c,d,g,h) embryos at E14.5 and E15.5. E14.5 and E15.5 mutant embryos (**B**) demonstrated hemorrhages. Sagittal sections of E14.5 control and mutant embryos (indicated in **B**) were stained with H&E (a–d), while transverse sections of E15.5 control and mutant embryos (indicated in **B**) were stained with H&E (e–h). In Fig. 2C(b,d,f,h) are higher magnifications of the red rectangular regions indicated in (a,c,e,g), respectively. The pink arrows indicate normal capillaries (b,f) with erythrocytes in the control embryos. The red ovals in (d,h) outline hemorrhaging of the body surface capillaries of mutant embryos, while the red arrow shows a normal great vessel (d) in a mutant embryo. (**D,E**) Histological analysis of control (a,b,e,f) and mutant (c,d,g,h) embryos at E13.5 and E14.5. No significant difference was found in the body surfaces of E13.5 control and mutant embryos (**D**, upper image), whereas the E14.5 mutant embryo appears pale compared to the E14.5 control embryo (**D**, lower image). Sagittal section of E13.5 and E14.5 control and mutant embryos (indicated in **D**) were stained with H&E. In Fig. 2E,(b,d,f,h) are higher magnifications of the red boxes indicated in (a,c,e,g), respectively. The pink arrows indicate normal capillaries (b,d,f,h) with or without erythrocytes, which are nucleated in both control and mutant embryos at this stage of development.

As shown in Supplementary Figure S3, we determined the approximate time of embryonic death in RCLG/EIIa-Cre embryos by *in vivo* bioluminescence imaging. To further ascertain the exact time and causes of embryonic death, we extensively analyzed the macroscopic appearance of 936 E12.5-E17.5 embryos resulting from the matings of RCLG mice and homozygous EIIa-Cre mice (Table 1). The macroscopic analysis identified abnormal embryos as early as E14.5 (Fig. 2A and Table 1). Whole-mount imaging of mutant embryos between E14.5 and E17.5 revealed distinct abnormal phenotypes, including hemorrhaging (13%, 53/405), resorbed embryos (51%, 207/405), pale body surface (15%, 62/405) and no abnormal appearance (20%, 83/405), while E11.5 to E16.5 littermate controls (Fig. 2A and Table 1) and RLG/EIIa-Cre control embryos (Supplementary Figure S7) showed normal development.

One of the most obvious abnormalities in the viable or unresorbed RCLG/EIIa-Cre embryos (i.e., mutant embryos) at E14.5 to E15.5 was severe hemorrhaging across body surface (Fig. 2A,B, Supplementary Figures S9 and S10 and Table 1), while the partially resorbed mutant embryos displayed widespread hemorrhages at E14.5 to E16.5 (Fig. 2A, Supplementary Figures S9–S11 and Table 1), indicating that hemorrhages might be the leading cause of embryo death. Moreover, the hemorrhages invariably preceded embryonic death, supporting a casual relationship.

Histological examination subsequently revealed that the hemorrhages only occurred under the skin (Fig. 2C,D,h) and did not occur in the internal organs [i.e., liver (Supplementary Figure S13) and other organs (data not shown)] of the E14.5 and E15.5 mutant embryos that exhibited bleeding. Histological analysis of E14.5 and E15.5 mutant embryos with grossly detectable hemorrhages showed blood cell leakage from body surface capillaries into subcutaneous tissues (Fig. 2C,D,h), while immunohistochemical (IHC) staining (for an endothelial marker CD34) of the sections from mutant embryos at E14.5-E15.5 revealed disrupted subcutaneous capillaries with the diffusion of red blood cells (Fig. 2C,D,h and Supplementary Figure S14-l,p). In addition, histological examination revealed that, similar to control embryos, all of E12.5 (Supplementary Figure S15D,F) and E13.5 (Fig. 2E-d) mutant embryos examined displayed normal, erythrocyte-containing capillaries under the subcutaneous tissues, suggesting that the subcutaneous capillaries of E12.5 and E13.5 mutant embryos can develop normally, as confirmed by evaluating the integrity of surface capillary with an endothelial marker (i.e., CD34) (Supplementary Figure S14d,h). Therefore, we suspect that the subcutaneous capillary lesions (with an unknown etiology) occur in those E14.5 and E15.5 mutant embryos with severe subcutaneous hemorrhages, thereby causing blood cell leakage from body surface capillaries into subcutaneous tissues.

Macroscopic analysis of the embryos from timed matings revealed the frequent occurrence of partially and completely resorbed mutant embryos (51%, 207/405) between E14.5 and E17.5 (Fig. 2A, Supplementary Figures S9–S12 and Table 1). A few mutant embryos were viable up to E17.5 (Table 1); however, no viable mutant embryos were observed at E18.5 or later (data not shown). Therefore, these findings suggest that EIIa-Cre-mediated activation of Cripto-1 transgene leads to embryonic lethality between E14.5 and E17.5.

The macroscopic analysis also revealed that 15% of E14.5, E15.5 and E16.5 mutant embryos were pale and lacked prominent vasculature when compared with control embryos (Fig. 2A,D, Supplementary Figures S9–S11 and Table 1), while histological examination revealed that E14.5 mutant embryos with pale body surface exhibited normal capillaries without erythrocytes under the subcutaneous tissues (Fig. 2E-h), suggesting that the pale body surface may be due to the lack of erythrocytes in the capillary blood vessels under the skin. Twenty percent of the 405 mutant embryos between E14.5 and E16.5 did not appear particularly abnormal (Fig. 2A, Supplementary Figures S9–S11 and Table 1). However, mutant embryos with pale body surface or no abnormal appearance were hardly found at E17.5 (Table 1), and were not observed at E18.5 or later (data not shown), suggesting that hemorrhaging may not be the only cause of embryonic lethality in these mice.

In summary, the sustained expression of Cripto-1 transgene in mouse embryos leads to hemorrhages and embryonic death. Severe hemorrhaging under the subcutaneous capillaries at E14.5-E16.5 might be the leading cause of embryo death.

### Cardiac defects in mutant embryos (RCLG/EIIa-Cre)

The addition of soluble Cripto-1 protein during the 0–2-d interval effectively restored the ability of Cripto-1^−/−^ embryonic stem cells (ES cells) to differentiate into cardiomyocytes, whereas the addition of the same protein at later time points dramatically reduced cardiomyocyte differentiation^16^, suggesting that Cripto-1 activity was required to promote specification of the cardiac lineage. These findings encouraged us to explore whether the sustained expression of Cripto-1 in embryos affects embryonic heart development.

**Figure 3:**
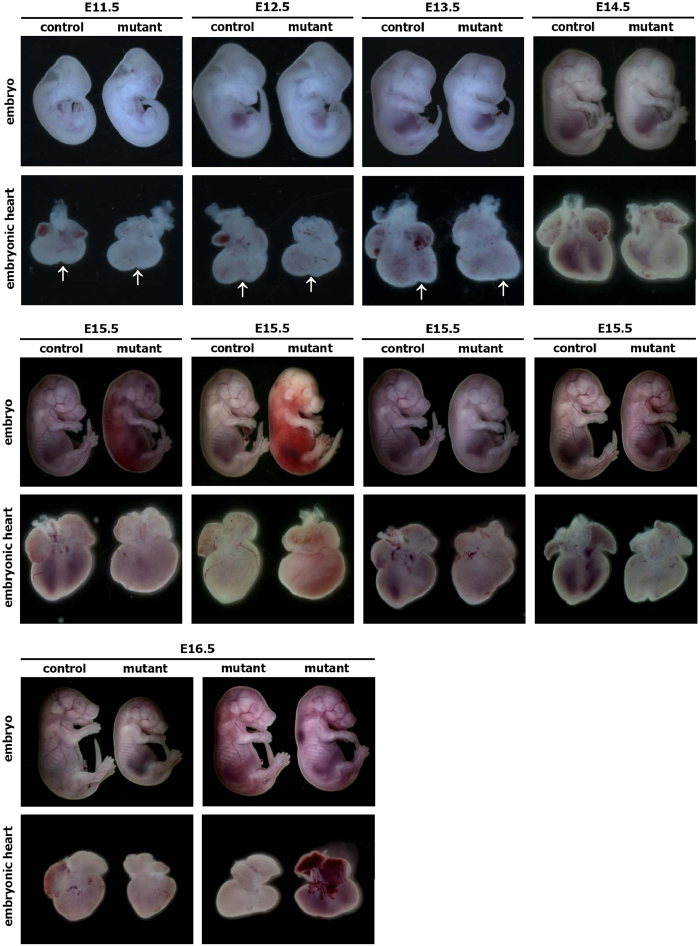
Whole-mount views of representative hearts obtained from control (EIIa-Cre) and mutant (RCLG/EIIa-Cre) embryos at E11.5 to E16.5. The lower images show the hearts of the E11.5 to E16.5 control and mutant embryos shown in the upper images. At E14.5, E15.5 and E16.5, all of the mutant embryos exhibiting hemorrhages, a pale body surface or no abnormal appearance displayed abnormal heart morphology. The arrows indicate the interventricular sulcus.

We first examined the gross morphology of the hearts from E11.5-E16.5 control and mutant embryos with stereomicroscope. No significant morphological abnormalities were detected at E11.5 (Fig. 3). The morphological differences between control and mutant hearts were evident one day later (E12.5), and the changes became obvious at E13.5 and later time points (Fig. 3). The ventricular wall of mutant hearts was often enlarged and rounded, with little external evidence of the interventricular sulcus (arrows) (Fig. 3). The appearance of the interventricular sulcus in control embryos is a consequence of interventricular septum formation. The lack of this anatomical marker is an indication of the abnormal morphogenesis of the septum in E12.5 and E13.5 mutant embryos. Furthermore, all of the embryonic hearts obtained from E14.5-E16.5 mutant embryos with the different phenotypes, including hemorrhage, resorbed, pale body surface and no abnormal appearance, displayed abnormal cardiac morphology (Fig. 3). Additionally, extensive whole-mount imaging of mutant and control hearts revealed similar defects in multiple E12.5-E16.5 mutant hearts.

**Figure 4:**
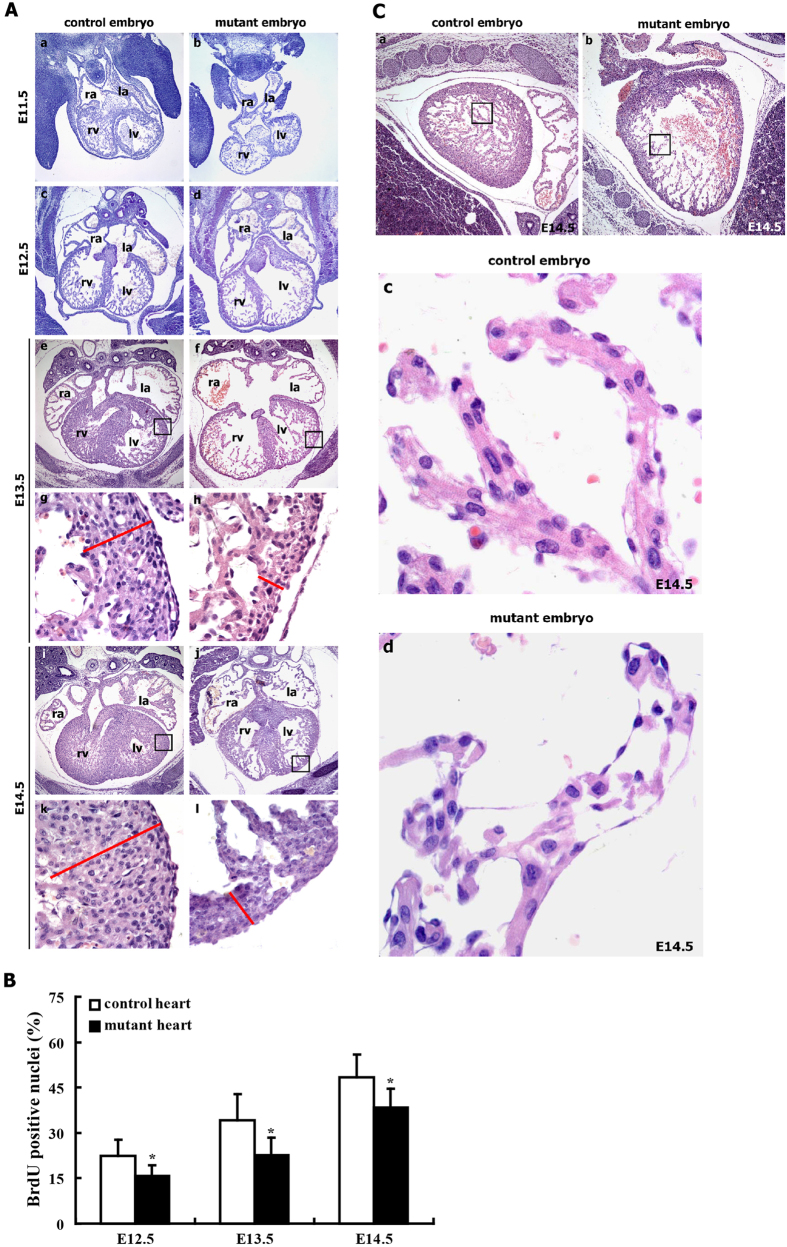
Cardiac defects in mutant embryos (RCLG/EIIa-Cre). (**A**) H&E staining of transverse sections of control and mutant embryos. The upper images show the entire hearts of E13.5 and E14.5 embryo, and the lower images (g,h,k,l) are higher magnifications of the rectangular regions indicated in images (e,f,i,j), respectively. Higher magnification of E13.5 and E14.5 hearts revealed thinning due to a decrease in the myocardial cell layers in mutant embryos. The red lines show the thickness of the myocardial layer of the left ventricle (lv). Abbreviations: ra, right atrium; la, left atrium; rv, right ventricle; lv, left ventricle. (**B**) The cardiomyocyte proliferation index was calculated by dividing the number of BrdU positive nuclei by the total number of nuclei in the E12.5, E13.5 and E14.5 mutant hearts. Representative BrdU staining of embryonic heart sections at E12.5-E14.5 is indicated in Supplementary Figure S16. (**C**) The cardiomyocytes in the mutant embryos failed to assemble striated myofibrils. (c,d) are higher magnifications of the regions indicated in (a,b), respectively.

Given the macroscopic cardiac defects observed in E12.5-E16.5 mutant embryos, we further analyzed the histological appearance of mutant embryos and control littermates at various developmental stages. Histological sections of E11.5–14.5 embryos revealed a narrow temporal window for the onset of cardiac defects in mutant embryos, in addition to revealing massive cardiac defects in both cardiac compact layer and cardiac trabeculations of E12.5-E14.5 mutant embryos (Fig. 4A). We were unable to detect any significant differences in the thickness of the cardiac compact layer of mutant and control hearts at E11.5 (Fig. 4A-a,b). Myocardial wall thinning first became apparent at E12.5 (Fig. 4A-c,d). E13.5-14.5 mutant hearts displayed a dramatic reduction in the thickness of myocardial cell layer compared with control hearts (Fig. 4A-e–l). Additionally, E12.5-E14.5 mutant hearts displayed few, thin cardiac trabeculae, whereas control hearts demonstrated many thick cardiac trabeculae (Fig. 4A-c–f,i,j), indicating poor trabeculation in mutant hearts. Moreover, the endothelial cells were closely attached to the myocardium and chordae tendineae in control hearts, whereas the endothelial cells of mutant hearts were detached from the myocardium and chordae tendineae (Fig. 4A-c–f,i,j). Extensive histological analysis revealed similar defects in several mutant hearts.

To determine the molecular mechanism underlying the myocardial thinning observed in mutant hearts, we evaluated myocardial proliferation. The proliferation rate was measured in E12.5, E13.5 and E14.5 embryos by analyzing BrdU incorporation in pregnant female mice. The mutant embryonic hearts at E12.5-E14.5 displayed reduced cellular proliferation (Fig. 4B and Supplementary Figure S16), suggesting that the reduction in the myocardial cell layer might result from reduced myocyte proliferation.

Together, these findings strongly demonstrate that the sustained expression of Cripto-1 in E12.5-E16.5 embryonic hearts seriously impairs embryonic heart development, as evidenced by abnormally thin myocardium, dilated cardiac chambers and poor trabeculation.

### Impaired coronary vessel formation in E14.5 mutant hearts (RCLG/EIIa-Cre)

There were no significant differences in coronary vascular formation between E12.5 control and mutant hearts, which displayed a very small number of blood vessels (Supplementary Figure S17A,B). An examination of histological sections revealed that the reduction in coronary vessel density first occurred in mutant hearts at E13.5 (Supplementary Figure S17C–F). E14.5 mutant hearts displayed a dramatic reduction in coronary vessel density compared with control hearts (Supplementary Figure S18). Similar defects were seen in multiple mutant embryos at E13.5 and E14.5. Collectively, these results strongly suggest that the ectopic expression of Cripto-1 impairs coronary vessel formation in mutant hearts at E13.5 and E14.5.

### E13.5 to E16.5 mutant hearts do not exhibit cardiac contractility

The abnormal morphology and structure of mutant hearts prompted us to determine whether the hearts of mutant embryos can beat normally. We performed time-lapse video microscopy (AZ-100, Nikon) to investigate the beating of hearts obtained from mutant embryos (RCLG/EIIa-Cre) *in vitro*. In these experiments, the heartbeats of control and mutant hearts obtained from E12.5 to E16.5 embryos were recorded (Supplementary Movies S1–S5). At E12.5, the control embryonic heart beat slowly and a small amplitude, whereas the heartbeat of mutant heart was not observed (Supplementary Movie S1). From E13.5 to E16.5, the control hearts beat at a regular rhythm, whereas the heartbeat of the mutant hearts was barely detectable (Supplementary Movies S2–S5), indicating the defects in cardiac contractility in mutant embryonic hearts at E13.5 to E16.5. Furthermore, we found that the cardiomyocytes in E14.5 mutant hearts failed to assemble into striated myofibrils (Fig. 4C), which play an important role in cardiac contraction. Collectively, the defects in heartbeat and myofibril assembly in mutant embryos suggest the possibility of abnormal cardiac function.

### Epicardial blisters in mutant mouse embryonic hearts

As shown in Supplementary Figure S19, the histological sections of E13.5 mouse embryonic hearts were employed to reveal the epicardial morphology of control (a,b) and mutant embryonic (c,d) hearts. The striking epicardial blisters (asterisks) were observed in the mutant mouse embryonic heart (Supplementary Figure S19).

### Temporally regulated Cripto-1 activation causes cardiac defects in mutant embryos (RCLG/hUb-CreERT2)

This expression pattern reveals that Cripto-1 plays a important role in the early stage, but not in the late stage of embryonic heart development^12^,^19^, which prompted us to determine whether the aforementioned cardiac defects was attributed to the ectopic Cripto-1 expression at later stages of embryonic heart development. The previous reports demonstrated that tamoxifen (TM)-inducible recombination can be used to effectively modify gene function in mouse embryos and most embryonic organs (i.e., embryonic heart)^20^,^21^. As shown in Supplementary Figure S20A, the homozygous RCLG mice are mated with the heterozygous hUb-CreERT2 mice^22^ to generate RCLG/hUb-CreERT2 embryos. To realize the activation of Cripto-1 and Luc transgene expression at the later stages of embryonic development, pregnant mice were treated with TM at 8.5 days postcoitum (dpc) (Supplementary Figure S20A). We detected the activation of Luc expression in RCLG/hUb-CreERT2 transgenic embryos from E10.5 to E14.5 after a single intraperitoneal (IP) injection of TM at 8.5dpc (Supplementary Figure S20B). After performing RT-PCR using primers specific for human Cripto-1, we detected the strong expression of Cripto-1 transgene in E10.5-E14.5 RCLG/hUb-CreERT2 embryos, but undetected Cripto-1 transgene expression in E8.5 RCLG/hUb-CreERT2 embryos (Supplementary Figure S20C). Moreover, we also detected Cripto-1 transgene expression in E11.5-E14.5 RCLG/hUb-CreERT2 embryonic hearts (Supplementary Figure S20C). Together, these data indicate that RCLG/hUb-CreERT2 embryos efficiently activate Cripto-1 transgene at the later stages (i.e., E10.5-14.5) of embryonic development after a single IP injection of TM at 8.5dpc.

**Figure 5:**
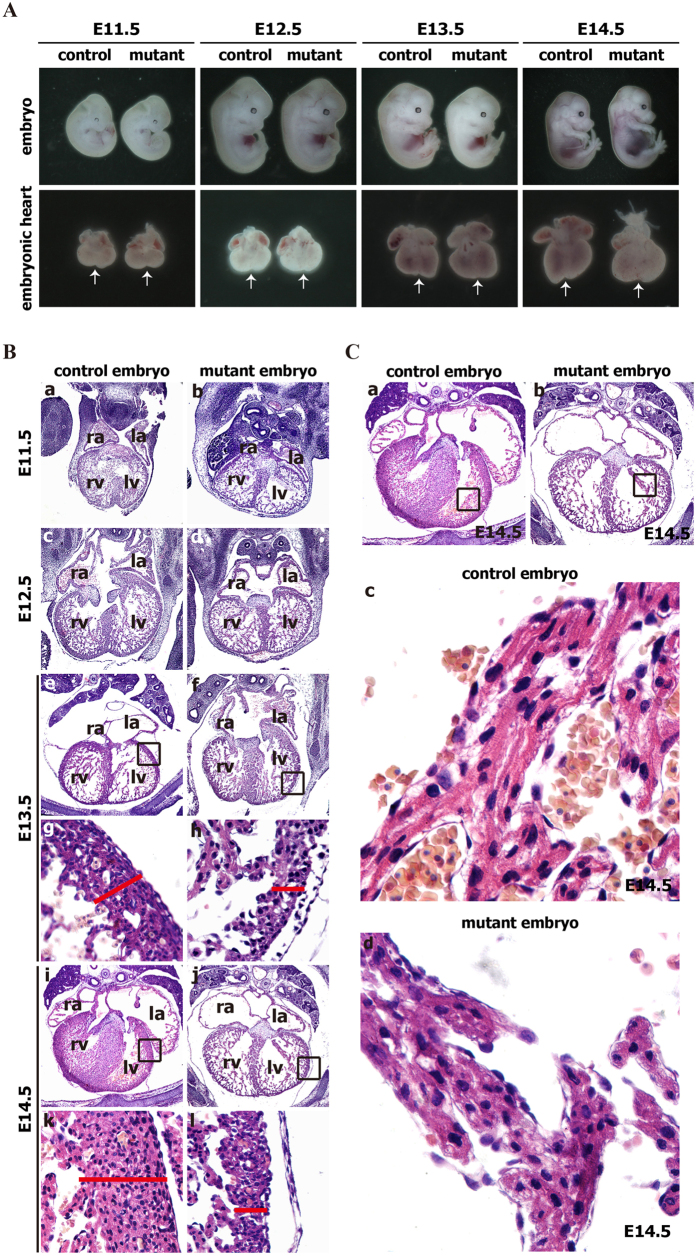
Temporally regulated Cripto-1 activation causes the cardiac defects in mutant embryos (RCLG/hUb-CreERT2). (**A**) Whole-mount views of representative hearts obtained from control (RCLG) and mutant (RCLG/hUb-CreERT2) embryos at E11.5 to E14.5. The lower images show the hearts of the E11.5 to E14.5 control and mutant embryos shown in the upper images. The arrows indicate the interventricular sulcus. (**B**) H&E staining of transverse sections of control and mutant embryos. The upper images show the entire hearts of E13.5 and E14.5 embryo, and the lower images (g,h,k,l) are higher magnifications of the rectangular regions indicated in images (e,f,i,j), respectively. Other details as in Fig. 4. (**C**) The cardiomyocytes in the mutant embryos failed to assemble striated myofibrils. (c,d) are higher magnifications of the regions indicated in (a,b), respectively.

Next, we first examined the gross morphology of the hearts from E11.5-E14.5 control and mutant (RCLG/hUb-CreERT2) embryos with stereomicroscope. No significant morphological abnormalities were detected at E11.5 (Fig. 5A). The morphological differences between control and mutant hearts were evident one day later (E12.5), and the changes became obvious at E13.5 and later time points (Fig. 5A). The ventricular wall of mutant hearts was often enlarged and rounded, with little external evidence of the interventricular sulcus (arrows) (Fig. 5A).

Given the macroscopic cardiac defects observed in E12.5-E14.5 mutant embryos, we further analyzed the histological appearance of mutant embryos and control littermates at various developmental stages. We were unable to detect any significant differences in the thickness of the cardiac compact layer of mutant and control hearts at E11.5 (Fig. 5B-a,b). Myocardial wall thinning first became apparent at E12.5 (Fig. 5B-c,d). E13.5-14.5 mutant hearts displayed a dramatic reduction in the thickness of the myocardial cell layer (Fig. 5B-e–l). Additionally, E12.5-E14.5 mutant hearts displayed few, thin cardiac trabeculae, whereas control hearts demonstrated many thick cardiac trabeculae (Fig. 5B-c–f,i,j), indicating poor trabeculation in mutant hearts.

The abnormal morphology and structure of mutant hearts prompted us to determine whether the hearts of mutant embryos can beat normally. We firstly found that the cardiomyocytes in E14.5 mutant hearts failed to assemble into striated myofibrils (Fig. 5C), which plays an important role in cardiac contraction. Based on the observation by time-lapse video microscopy, we found that E13.5 and E14.5 control hearts beat at a regular rhythm, whereas the heartbeat of E13.5 and E14.5 mutant hearts was barely detectable (Supplementary Movies S6 and S7), indicating the defects in cardiac contractility in mutant embryonic hearts at E13.5 and E14.5. Collectively, the defects in myofibril assembly and heartbeat in mutant embryos suggest the possibility of abnormal cardiac function.

Taken together, these findings strongly demonstrate that Cripto-1 transgene activation in RCLG/hUb-CreERT2 embryos at later developmental stages seriously impairs embryonic heart development, as evidenced by abnormally thin myocardium, dilated cardiac chambers, poor trabeculation, failure to assemble striated myofibrils and the lack of fetal heartbeat, similar to what was observed upon the sustained expression of Cripto-1 in RCLG/EIIa-Cre embryonic hearts at E12.5-E16.5.

### Reduced cardiac gene expression in mutant embryos

**Figure 6:**
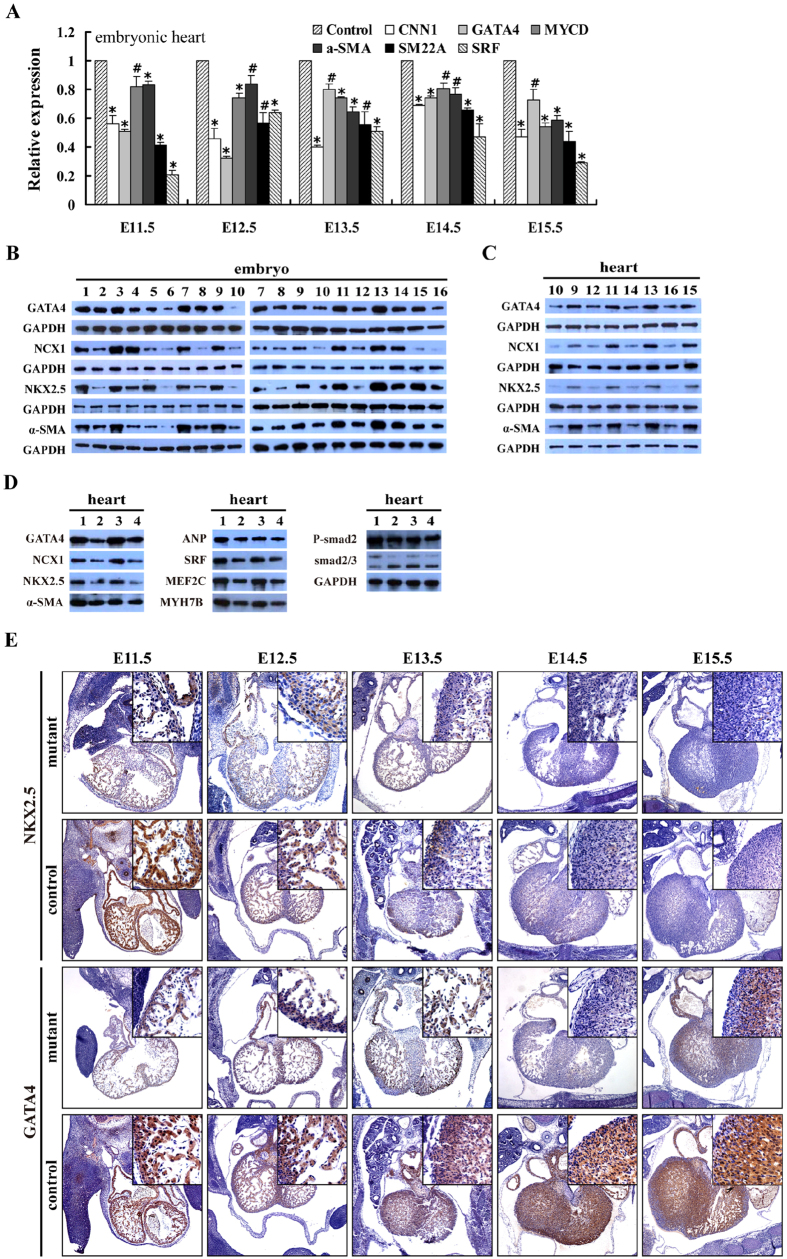
Mutant embryonic hearts exhibit altered cardiac gene expression. (**A**) qRT-PCR for the indicated gene expression in mutant embryonic hearts (RCLG/EIIa-Cre). The cropped gels are used in Fig. 6A, and the full-length gel images are available in Supplementary Figure S28. The gels have been run under the same experimental conditions. (**B,C**) Western blot for the indicated proteins in E7.5-E14.5 mutant embryos and E11.5-E14.5 hearts (RCLG/EIIa-Cre). GAPDH was used as a control for the Western blot. Lane 1: Cre(E7.5); lane 2: RCLG/Cre(E7.5); lane 3: Cre(E8.5); lane 4: RCLG/Cre(E8.5); lane 5: Cre(E9.5); lane 6: RCLG/Cre(E9.5); lane 7: Cre(E10.5); lane 8: RCLG/Cre(E10.5); lane 9: Cre(E11.5); lane 10: RCLG/Cre(E11.5); lane 11: Cre(E12.5); lane 12: RCLG/Cre(E12.5); lane 13: Cre(E13.5); lane 14: RCLG/Cre (E13.5); lane 15: Cre(E14.5); lane 16: RCLG/Cre(E14.5). The cropped blots are used in Fig. 6B,C, and the full-length gel images are available in Supplementary Figure S28. The blots have been run under the same experimental conditions. (**D**) Western blot analysis of the indicated proteins in E11.5 and E14.5 mutant embryonic hearts (RCLG/hUb-CreERT2). Lane 1: RCLG (E11.5); lane 2: RCLG/Cre(E11.5); lane 3: RCLG (E14.5); lane 4: RCLG/Cre(E14.5). The cropped blots are used in Fig. 6D, and the full-length gel images are available in Supplementary Figure S28. The blots have been run under the same experimental conditions. (**E**) Immunohistochemical (IHC) analyses of Nkx2.5 and GATA4 expression in RCLG/EIIa-Cre embryonic hearts. ANF, atrial natriuretic peptide; α-SMA, α-smooth muscle actin; CNN1, calponin 1; MEF2C, Myocyte-specific enhancer factor 2C; MYCD, myocardin; MYH7B, myosin, heavy chain 7B; NCX1, cardiac sodium–calcium exchanger; SM22A, smooth muscle protein 22 alpha; SRF: serum response factor.

Given the macroscopic and microscopic cardiac defects observed in E12.5-E14.5 mutant embryos, we decided to look for potential changes in the expression pattern of cardiogenic transcription factors at 7.5-15.5 days of development. Western blot analysis indicated that the expression of genes (i.e., GATA4, NCX1, Nkx2.5 and α-SMA) involved in cardiac differentiation was down-regulated in E7.5-E14.5 RCLG/EIIa-Cre embryos (Fig. 6B), while both qRT-PCR and Western blot analysis revealed the reduced expression of cardiac/SMC markers (i.e., GATA4, NCX1, Nkx2.5, α-SMA, CNN1, MYCD, SM22A and SRF) in RCLG/EIIa-Cre hearts from E11.5 to E14.5 (Fig. 6A,C). Moreover, Western blot analysis illustrated the reduced expression of genes, including GATA4, NCX1, Nkx2.5, α-SMA, ANF/ANP, SRF, MEF2C and MYH7B, and the reduced phospho-Smad2 level in the hearts from E11.5 and E14.5 RCLG/hUb-CreERT2 embryos (Fig. 6D).

The expression of Nkx2.5 and GATA4 in the hearts from E11.5 to E15.5 mutant embryos was subsequently detected by IHC (Fig. 6E). In general, we found that the immunoreactivity signal of the Nkx2.5 in the cardiomyocytes of control embryos gradually weakened from E11.5 to E13.5, until it became nearly (at E14.5) or completely (at E15.5) undetectable (Fig. 6E). In contrast, GATA4 expression maintained constant expression levels in the cardiomyocytes from E11.5 to E15.5 control embryos (Fig. 6E). We found that Nkx2.5 was downregulated in the cardiomyocytes of E11.5 to E13.5 mutant embryos, and was completely undetectable in the cardiomyocytes of mutant embryos at E14.5 and E15.5 (Fig. 6E). Furthermore, GATA4 expression was significantly reduced in cardiomyocytes of E11.5 to E15.5 mutant embryos (Fig. 6E).

### Mutant embryos exhibit aberrantly elevated levels of nucleated erythrocytes at E14.5 and E15.5

**Figure 7:**
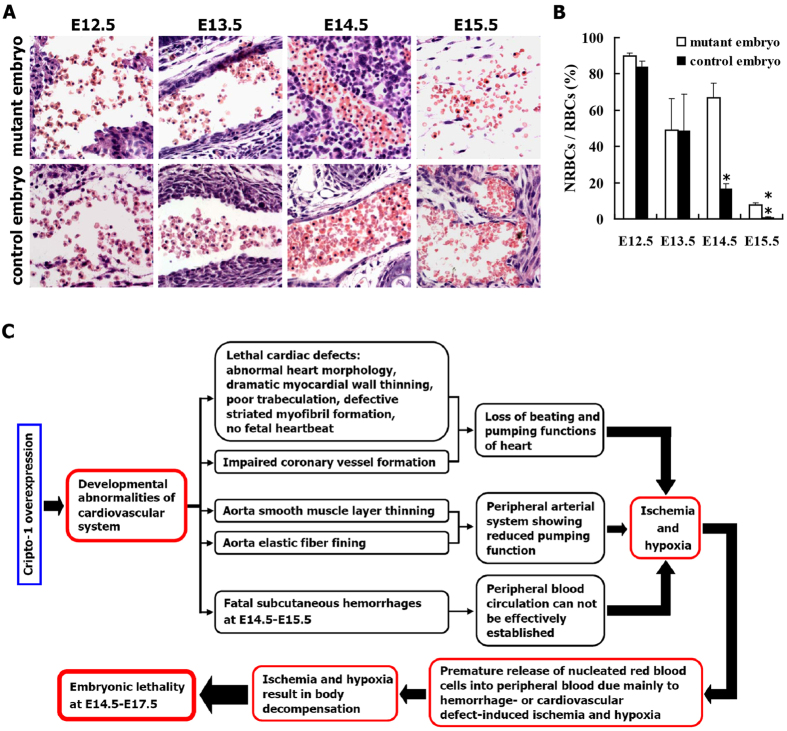
(**A,B**) Mutant embryos (RCLG/EIIa-Cre) exhibit aberrantly elevated levels of primitive erythrocytes at E14.5 and E15.5. (**A**) H&E staining of paraffin sections of representative E12.5 to E15.5 control and mutant embryos were stained with H&E. These representative sections focused on showing the nucleated red blood cells (NRBCs) in mutant and control embryos between E12.5 and E15.5. (**B**) The percentage of nucleated red blood cells (NRBCs) in E12.5 to E15.5 embryos. RBCs: red blood cells. (**C**) The mechanisms underlying embryonic lethality due to constitutive Cripto-1 expression in transgenic mice.

At E12.5, the peripheral red blood cells present in the control and mutant embryos were primarily nucleated immature erythrocytes (Fig. 7A). The number of nucleated red blood cells (NRBCs) in the peripheral blood of control embryos gradually decreased from E13.5 to E15.5 (Fig. 7A,B), and was almost undetectable at E16.5 (data not shown). As shown in Fig. 7A,B, there were no significant differences in the percentage of NRBCs in the peripheral blood of control and mutant embryos at E12.5 and E13.5. Compared with the samples from control embryos, the sections obtained from E14.5 and E15.5 mutant embryos showed a greater than 4-fold increase and a 10-fold increase in the percentage of nucleated erythrocytes in peripheral blood, respectively (Fig. 7A,B), suggesting that the decrease in the number of NRBCs in the peripheral blood of mutant embryos was slower than the decrease that occurred in control embryos. Thus, our results illustrate that E14.5 and E15.5 mutant embryos contain an aberrantly high level of nucleated erythrocytes in peripheral blood.

## Discussion

In this work, we extensively studied the phenotypes of mice that ubiquitously express Cripto-1 throughout development. This strategy was used in an attempt to explore the effects of increasing the levels of Cripto-1 during late embryogenesis. Our macroscopic and histological examination revealed that the sustained expression of Cripto-1 transgene after E11.5 led to fatal embryonic hemorrhages under subcutaneous capillaries (at E14.5 and E15.5), lethal cardiac defects (between E12.5 and E16.5) and vascular defects (see “Supplementary Results” for details). These cardiac defects primarily include abnormal cardiac morphology, myocardial wall thinning, dilated cardiac chambers, poor trabeculation, impaired coronary vessel formation, failure to assemble striated myofibrils and lack of fetal heartbeat, which in turn results in the loss of the heart’s blood pumping function and embryonic death.

During embryonic development, Cripto-1 is expressed in mouse blastocyst and primitive streak, and later is restricted to the developing heart^12^,^19^. Cripto-1 is expressed in the myocardium of the developing heart tube of E8.5 embryos and in the outflow region, or conotruncus, of E9.5-E10 embryonic heart. E9.5-E10 is the time when the heart develops into a functional chambered organ, and no *in situ* hybridization signals for Cripto-1 are detected after E10.5^12^,^19^. This expression pattern suggests that Cripto-1 may play a role in the early events leading to heart morphogenesis, but not in the late stage of embryonic heart development, as strongly supported by the following *in vitro* and *in vivo* findings from other groups^9^,^12^,^16^,^17^,^19^ and this study.

The disruption of Cripto-1 in mice led to a complete lack of cardiomyocyte differentiation *in vivo*^9^, and embryoid bodies (EBs) derived from Cripto-1^−/−^ ES cells failed to produce contracting cardiomyocytes even during extended culture periods^17^, indicating that Cripto-1 is critical for myocardial development^9^,^15^,^16^,^17^.

The previous study demonstrated that the addition of soluble Cripto-1 protein during the 0–2 day interval effectively restored the ability of Cripto-1^−/−^ ES cells to differentiate into cardiomyocytes, however, the addition of Cripto-1 at later time points dramatically reduced cardiomyocyte differentiation^16^. These functional data are supported by the expression data showing that endogenous Cripto-1 protein is present at the earliest stages of ES cell differentiation but absent at stages where contracting EBs start to appear^16^. These data suggest that Cripto-1 signaling is required for the cardiac commitment of ES cells rather than the terminal differentiation of cardiomyocytes in culture, suggesting that Cripto-1 acts early to determine cardiac fate *in vitro*^16^. More importantly, these data reveal that the endogenous Cripto-1 down-regulation is critical for late phases of ES cell-derived cardiomyocyte differentiation *in vitro*, as supported by *in vivo* findings from this study.

As mentioned above, Cripto-1 is required for embryonic heart development. However, in this study, we unexpectedly found that the sustained expression of Cripto-1 transgene in RCLG/EIIa-Cre embryos led to lethal cardiac defects. More importantly, Cripto-1 transgene activation in RCLG/hUb-CreERT2 embryos after E8.5 by TM administration imitated the phenotypes of lethal cardiac defects observed in RCLG/EIIa-Cre embryos. Moreover, Oct4 is expressed throughout the neural plate of mouse embryos until E8.0; after E9.5, ectopic Oct4 expression in this region caused cell death and affected forebrain development, suggesting that Oct4 down-regulation at later stages is necessary for normal brain development^23^. Together, these aforementioned observations from other groups^9^,^12^,^16^,^17^,^19^,^23^ and this study support this hypothesis that Cripto-1 acts early to specify cardiac fate, but that Cripto-1 down-regulation at later stages of embryonic heart development is necessary for proper cardiac morphogenesis, as the sustained expression of Cripto-1 in E11.5-E16.5 embryonic hearts severely impairs cardiac morphogenesis and therefore impairs cardiac function.

We found that the ectopic expression of Cripto-1 transgene in embryonic hearts resulted in lethal cardiac defects, accompanied by a general downward trend in the expression profile of essential cardiac transcription factors, including GATA4, NCX1, Nkx2.5 and SRF. Disruption of GATA4^24^, Nkx2.5^25^, SRF^26^,^27^ and SRF-dependent gene NCX1^28^ in mouse embryos led to embryonic lethality due to the aforementioned lethal cardiac defects, accompanied by the reduced cardiac gene expression, similar to what was observed upon ectopic Cripto-1 transgene expression in embryonic hearts. It is well known that Nkx2.5 and GATA4 are two early markers of precardiac cells, and overexpression of Nkx2.5 and GATA4 enhances cardiac development in committed precursors^29^,^30^. As shown in Fig. 6, our findings revealed the significant reduced expression of two early markers (i.e., Nkx2.5 and GATA4) of the myocardial precursors in mutant embryonic hearts, suggesting the cardiac developmental defects in committed cardiac precursors, thereby causing fatal cardiac defects. The BMP and Smad pathways are required for normal cardiac development *in vivo*, while temporal and spacial precise regulation of the Smads activities is important for normal cardiac development from initial cardiomyocyte differentiation to terminal cardiac morphogenesis^16^,^31^. Additionally, BMPs induce the expression of cardiac-specific genes and cardiomyocyte differentiation in P19CL6 cells and ES cells through activating the Smad pathway^16^,^31^. In this study, we observed a significant decrease in Smad2 phosphorylation in mutant embryonic hearts at E11.5 and E14.5. We suspect that the reduced phospho-Smad2 level in mutant embryonic hearts further induces the down-regulation of the above-mentioned cardiac transcription factors, which in turn leads to multiple cardiac defects. Together, reduced expression of the aforementioned essential regulators of embryonic heart development is likely at least partially responsible for lethal cardiac defects observed in mutant embryonic hearts.

Na^+^-Ca^2+^ exchanger 1 (NCX1) serves a housekeeping function by regulating intracellular Ca^2+^ concentration in heart^28^. The severe disorganization of myofibrils and lack of fetal heartbeat were reported in NCX1-deficient mice^28^ and SRF-deficient mice^26^. Moreover, the depressed expression of other myocardial transcription factors (such as GATA4, Nkx2.5, MYCD and MYH7B) involved in cardiac contraction causes reduced cardiac contraction^24^,^25^,^32^. Our results revealed that mutant embryonic hearts displayed the failed assembly of striated myofibrils and lack of fetal heartbeat, accompanied by the significantly deceased expression of SRF, NCX1, GATA4, Nkx2.5, MYCD and MYH7B, which is likely to at least partially contribute to myocardial contractile defects. Therefore, our results suggest that the lack of fetal heartbeat resulted from the diminished expression of the above-mentioned cardiac contraction-related genes is, at least in part if not all, the leading cause of embryo lethality in mutant embryos with pale body surface or no abnormal appearance. In summary, the reduced expression of these critical cardiac transcription factors provides a potential explanation for the multiple defects in cardiac morphology, structure and function in mutant embryonic hearts.

As shown in Table 1, E14.5-E17.5 mutant embryos exhibited complex phenotypes, such as subcutaneous capillary bleeding (at E14.5-E15.5), lethality (at E14.5-E17.5), pale body surface (at E14.5-E16.5) and no abnormal appearance (at E14.5-E16.5). Few mutant embryos survived to E17.5, and no viable mutant embryos were found at E18.5 or later (data not shown). Based on these results, we comprehensively analyzed the reasons underlying embryonic lethality. One of the most apparent abnormalities in the viable or unresorbed mutant embryos at E14.5-E15.5 was severe hemorrhaging throughout body surface, while partially resorbed mutant embryos (at E14.5-E16.5) displayed widespread hemorrhages, indicating that mutant embryos with widespread bleeding made up a large proportion of 405 mutant embryos (at E14.5-E16.5) analyzed. As shown in Fig. 7C, this severe subcutaneous capillary bleeding led to blood cell leakage from body surface capillaries into subcutaneous tissues, causing ischemia and hypoxia due to the failure to establish effective peripheral blood circulation. This bleeding finally resulted in the death of E14.5 to E16.5 mutant embryos. Additionally, the significant increase in the number of nucleated erythrocytes in E14.5 and E15.5 mutant embryos resulted from the premature release of nucleated red blood cells into the peripheral blood due to ischemia and hypoxia (Fig. 7C), a phenomenon known as stress erythropoiesis. Collectively, our results suggest that subcutaneous capillary hemorrhage is the leading cause of mutant embryo death.

Furthermore, mutant embryos with pale surface or no abnormal appearance were frequently seen at E14.5-E16.5, but were hardly found at E17.5 and were not observed at E18.5 or later (data not shown), suggesting that reason(s) other than hemorrhage might also lead to embryonic lethality. As shown in Fig. 7C, the ectopic Cripto-1 expression caused the above-mentioned cardiac defects, which in turn caused mutant embryonic hearts to be unable to pump blood, and finally led to embryonic lethality at E16.5 and E17.5. Therefore, lethal cardiac defects might be the leading cause of embryo death in mutant embryos with pale body surface or no abnormal appearance.

In summary, our results show that the severe hemorrhages under the skin are the primary cause of lethality at E14.5 and E15.5, and the massive cardiac defects may be the leading cause of embryo death (at E16.5 and E17.5) in those mutant embryos without detectable hemorrhages, pale body surface or no abnormal appearance, strongly suggesting that hemorrhages and cardiac abnormalities are two important lethal factors.

As described in “Supplementary Results” section, the thoracic and abdominal aortas of E11.5, E12.5, E13.5 or E14.5 mutant embryos are thinner than the aortas of control embryos due to a decrease in smooth muscle cell layers. As shown in Fig. 7C, the thin layers of poorly organized smooth muscle cells and the network of thin elastic fibers in the aortas of mutant embryos dramatically reduce the ability of peripheral arterial system to pump blood, thereby aggravating the ischemia and hypoxia in mutant embryos.

The previous report revealed that Cripto-1 can promote the proliferation, migration and invasion of human umbilical vein endothelial cells (HUVECs), induce HUVEC differentiation into vascular-like structures on Matrigel, and stimulate angiogenesis *in vivo*^33^. Interestingly, as described in “Results” section, the subcutaneous capillary lesions (with an unknown etiology) occur in those E14.5 and E15.5 mutant embryos with hemorrhages across body surface, thereby causing blood cell leakage from disrupted body surface capillaries into subcutaneous tissues. Our findings suggest that the ectopic Cripto-1 expression may compromise endothelial cell function (vascular leakage) in E14.5 and E15.5 mutant embryos with severe subcutaneous hemorrhages, which is not consistent with the above-described findings on the function of Cripto-1 in promoting angiogenesis^33^. The exact cause(s) of subcutaneous capillary disruption and hemorrhages in E14.5 and E15.5 mutant embryos and underlying mechanisms remain to be characterized.

As shown in Fig. 7C, the following combined causes, including (1) loss of beating and pumping functions of mutant fetal hearts, (2) reduced pumping function of peripheral arterial system in mutant fetal hearts and (3) failure to establish effective peripheral blood circulation in mutant embryonic hearts, result in ischemia and hypoxia in mutant embryos. The previous study demonstrated that during hypoxia, Cripto-1 expression levels were significantly elevated in ES cells, while in a porcine animal model of ischemia and myocardial infarction (MI) and in human hearts obtained from MI patients, Cripto-1 expression was dramatically elevated in the acut and protracted phases following MI^15^, suggesting that hypoxia may regulate Cripto-1 *in vitro* and *in vivo*. Additionally, Cripto-1 is required for hypoxia to fully differentiate ES cells into cardiomyocytes^15^. In the future, we will further determine whether the hypoxia in mutant embryos due to the aforementioned combined causes may also contribute to wide spread cardiac defects in mutant embryonic hearts.

The transcription factor GATA4 is a critical regulator of cardiac gene expression and is known to modulate cardiomyocyte differentiation. GATA4 has been identified as a nodal regulator of cardiac angiogenesis, and the deletion of GATA4 from cardiomyocytes reduces myocardial capillary density^34^. The E14.5 mutant hearts observed in this study exhibited dramatically reduced coronary vessel density. Thus, the reduced expression of GATA4 in mutant hearts might be the cause of the coronary vessel formation defect observed in E14.5 mutant hearts. The formation of the coronary vessels is a fundamental event in heart development, and defects in the coronary vascular system thus have a significant impact on heart function. The mechanism underlying the impaired formation of coronary vessels in embryos expressing ectopic Cripto-1 remains to be fully investigated.

As mentioned above, our results indicated that the ectopic expression of Cripto-1 in transgenic mouse embryos after E11.5 led to fatal subcutaneous capillary hemorrhages, lethal cardiac defects and vascular defects. Several previous studies have also reported distinct phenotypes associated with the constitutive expression of EGF-CFC genes *in vivo*. For instance, gain-of-function experiments in chick embryos illustrated that addition of soluble Cripto to chick tissues suppressed posterior mesodermal fates and promoted anterior mesendodermal fates^11^. The overexpression of human Cryptic in zebra fish led to developmental delays and abnormal gastrulation movements^35^, whereas the re-expression of secreted forms of oep enhanced dorsoanterior development^36^. In chick embryos, the implantation of cells expressing human Cryptic, mouse Cryptic or chick Cripto on the right side of Hensen’s node led to the randomization of cardiac looping^37^. Moreover, the overexpression of frog FRL-1 in Xenopus animal caps caused neural induction in the absence of mesoderm formation^38^,^39^,^40^. Collectively, these findings display the various gain-of-function phenotypes resulting from the constitutive expression of EGF-CFC genes in mice, chick embryos, zebra fish and frogs.

Taken together, our *in vivo* findings demonstrate that the ectopic expression of Cripto-1 in mouse embryos after E11.5 leads to fatal hemorrhages under subcutaneous capillaries, lethal cardiac defects and vascular defects, which in turn cause embryonic lethality between E14.5 and E17.5. These results suggest that Cripto-1 down-regulation at these later stages is necessary for normal cardiovascular development to proceed.

## Materials and Methods

### Mice

The wild-type FVB/N mice, the homozygous R26R reporter mice (B6; 129S4-Gt(ROSA)26Sortm1Sor/J)^41^, the homozygous EIIa-Cre transgenic mice (FVB/N-Tg(EIIa-Cre)C5379Lmgd/J)^42^ and the heterozygous hUb-CreERT2 mice [129S.Cg-Tg(UBC-cre/ESR1)1Ejb/J; Stock Number: 007179]^22^were obtained from the Model Animal Research Center of Nanjing University, China. The wild-type ICR mice were purchased from Cyagen Biosciences (Guangzhou) Inc., China. The EIIa-Cre mice^42^ carry a Cre transgene under the control of a zygotically expressed (EIIa-Cre) promoter that activates the expression of Cre recombinase in the early mouse embryo. EIIa-Cre-mediated recombination occurs in a wide range of tissues, including the germ cells that transmit the genetic alteration to progeny (http://jaxmice.jax.org/strain/003724.html). hUb-CreERT2 mice were generated through lentitransgenesis using a lentivirus that expresses the Cre-ERT2 from the human ubiquitin C promoter^22^. All of the animal care and experiments were performed according to the Study and Ethical Guidelines for Animal Care, Handling and Termination established by the Southern Medical University subcommittee on laboratory animal care. The present work was approved by the ethical committee of Southern Medical University and is covered by Chinese animal husbandry legislation.

### Production of the RCLG transgenic mice

The pCI-cropto-1^43^ and pCAG-RLG^44^,^45^ vectors were generously provided by Dr. David S. Salomon (Center for Cancer Research, National Cancer Institute, USA) and Prof. Manuela Martins-Green (University of California, USA), respectively. A 600 bp fragment containing the Cripto-1 cDNA was amplified from the pCI-cropto-1 plasmid using PCR^43^, after which the cDNA was then cloned into the *Sma* I site of the pCAG-RLG^44^,^45^ parental vector to generate the RCLG transgenic construct, which is designated as pCAG-RCLG. The vector was characterized molecularly by PCR, enzyme digestion analysis and DNA sequencing (data not shown).

A potent, ubiquitous CMV/β-actin promoter (CAG promoter) was used to drive a series of cassettes in the pCAG-RCLG vector, including a floxed mRFP followed by three copies of a transcription-stopping polyA sequence (3 × PolyA) and a downstream internal ribosome entry site (IRES)-based bicistronic transcript, which included open-reading frames for Cripto-1 and firefly luciferase (Luc). As shown in Fig. 1A, only mRFP will be transcribed in the absence of Cre-mediated recombination, as the expression of the Cripto-1 and Luc transgenes is prevented by a STOP sequence flanked by *lox* P sites. When Cre-mediated recombination occurs, the floxed mRFP +3 × PolyA is excised, and then Cripto-1 and Luc are expressed in a diffuse pattern in RCLG/EIIa-Cre double transgenic mice (Fig. 1A). The construct map is not drawn to scale. Abbreviations: CAG promoter: CMV early enhancer/chicken β actin promoter; mRFP: monomeric red fluorescent protein; Luc: firefly luciferase; pA: polyadenylation signal; ▶: *lox* P site.

RCLG transgenic mice were generated by microinjecting DNA into the pronuclei of fertilized embryos using standard techniques that have previously been described^46^. The FVB/N strain was used as the source of embryos for micromanipulation and the subsequent breeding trials. All of the transgenic lines were created on the FVB/N genetic background. For the microinjection, the 9.436 kb fragment of RCLG transgene (Fig. 1A) was released from the pCAG-RCLG vector backbone by digestion with *Ssp* I and *Sfi* I, and was subsequently isolated and purified using the QIA quick gel extraction kit (Qiagen, Germany). The fragment was diluted to a final concentration of 2 μg/ml in DNA injection buffer (10 mM Tris/0.1 mM EDTA, pH 7.4) and then microinjected into the pronuclei of fertilized FVB/N embryos. About 20~30 injected eggs were transferred into the oviducts of one pseudopregnant ICR mouse and developed to term. Two to three days after birth, the offspring were screened to identify potential RCLG transgenic founders using an mRFP assay by the IVIS Lumina II imaging system (Xenogen Corp., Alameda, CA, USA). The results of this assay were subsequently confirmed with PCR-based genotyping.

### Whole animal (newborn, *in vivo*) and embryonic (*ex vivo*) fluorescence imaging

In this study, juvenile mice (3–5 days old) were imaged in the Xenogen IVIS Lumina II Imaging System and analyzed for fluorescence based on the manufacturer’s recommendations (Xenogen, Alameda, CA), as described previously^47^,^48^,^49^. For the *ex vivo* fluorescence imaging of embryos, fresh mouse embryos were dissected into ice-cold PBS, placed on a 10 cm plate, and then visualized for mRFP fluorescence using a stereo fluorescence microscope (Nikon, AZ100) or the IVIS Lumina II Imaging System (IVIS Imaging System)(Xenogen Corp., Alameda, CA, USA), as described previously^47^,^48^,^49^,^50^.

### Genotype analysis by PCR

PCR was performed on tail genomic DNA to further identify which mice had integrated RCLG into their genome. The forward primer (FP) and reverse primer (RP) sequences used to amplify a fragment of the RCLG transgene were: 5′-GGGAGCGCGTGATGAAC-3′ (FP) and 5′-CGTTGTGGGAGGTGATGTC-3′ (RP). The PCR conditions were as follows: pre-denaturation at 94 °C for 7 min, followed by 30 amplification cycles of denaturation at 94 °C for 1 min, primer annealing at 54 °C for 1 min, and extension at 72 °C for 30 s, and finally an additional extension at 72 °C for 10 min. RCLG construct DNA was used as the positive control for each PCR reaction, and genomic DNA from wild type mice was employed as a negative control for each PCR.

### Establishment of homozygous RCLG transgenic mouse colonies by *in vivo* fluorescence imaging

At 6-8 weeks of age, the RCLG transgenic founder was mated with wild-type FVB/N mice to generate the F1 generation. The mRFP-positive F1 animals derived from this founder were interbred to generate the F2 generation. The mRFP assay was used to determine the genotypes of the founder progeny and transgene inheritance (see above for details). Homozygous RCLG transgenic mice were readily distinguished from the F2 generation derived from the RCLG transgenic founder immediately after birth by using *in vivo* fluorescence imaging as described previously^47^,^48^,^49^.

### *In vivo* and *ex vivo* imaging of firefly luciferase (Luc) activity

Heterozygous or homozygous RCLG mice were crossed to homozygous EIIa-Cre mice to generate RCLG/EIIa-Cre double transgenic mice, in which Luc expression was activated in a diffuse pattern. The bioluminescence of these mice was measured non-invasively using the IVIS Imaging System, as described previously^47^,^48^,^49^,^50^,^51^,^52^. For the *in vivo* imaging of Luc activity, mice were firstly intraperitoneally injected with firefly D-luciferin (150 mg/kg; Caliper Life Sciences, USA). Seven to eight minutes after D-luciferin injection, anesthetized adult mice (3% isoflurane) or non-anesthetized newborn mice were placed in the IVIS imaging system. The bioluminescence signals were repeatedly acquired from the entire body of the animal until the maximum number of photons was obtained (Xenogen, Alameda, CA, USA). The image analysis and quantification of bioluminescence were performed using Living Image software (Xenogen Corp).

For the *ex vivo* bioluminescence imaging of isolated embryos, mice were injected with D-luciferin (150 mg/kg; Caliper Life Sciences, USA) and subsequently anesthetized with isoflurane. After the photon accumulation reached a maximum level, the mice were sacrificed, the embryos were rapidly excised, and the dissected embryos were imaged with the IVIS system.

Additionally, to save D-luciferin, the pregnant mice were first sacrificed, and then the embryos were rapidly excised and placed on a 10 cm plate. The collected embryos were then intraperitoneally injected with D-luciferin, and the dissected embryos were imaged with the IVIS system.

### RNA isolation, RT-PCR and quantitative real-time PCR (qRT-PCR)

For the mRNA analyses, total RNA from embryos or embryonic hearts was extracted using Trizol Reagent (TaKaRa) according to the protocol provided by the manufacturer. Total RNA was reversely transcribed with the PrimeScript RT reagent Kit (TaKaRa). The expression of the human Cripto-1 transgene in RCLG/EIIa-Cre or hUb-CreERT2 transgenic embryos and the corresponding controls was analyzed by RT-PCR using primers specific for human Cripto-1. qRT-PCR was used to determine the expression of the indicated mRNA transcripts.

The qRT-PCR analysis was performed using SYBR Green qRT-PCR master mix (TaKaRa) in a Stratagene Mx3005P qRT-PCR System as described. GAPDH was used as a control and for normalization in the RT-PCR and qRT-PCR assays. The primers used for the amplification of the indicated genes are listed in Supplementary Table S1. For the qRT-PCR analysis, all of the samples were normalized to internal controls, and the fold changes were calculated through relative quantification (2^−ΔΔCt^).

### Western blot analysis

The protocols for Western blot assay were previously well described^53^,^54^,^55^,^56^. For the Western blot analysis, total protein was extracted from embryos or embryonic hearts. The protein lysates were separated by SDS-PAGE and electrophoretically transferred to PVDF (polyvinylidene difluoride) membrane. Then, the blots were probed with primary antibodies (shown in Supplementary Table S2) followed by HRP (horseradish peroxidase)-labeled goat anti-mouse or goat anti-rabbit IgG. The signals were then detected using enhanced chemiluminescence (ECL). GAPDH was used as a protein loading control. For the source and a description of all antibodies used in this study, see Supplementary Table S2.

### Whole-mount X-gal staining and histology

To evaluate the EIIa-Cre mice-mediated excision efficiency in embryonic heart, EIIa-Cre mice were mated with a Cre-dependent Lac Z reporter strain (R26R reporter mice)^41^ to generate R26R/EIIa-Cre double transgenic embryos. In these mice, Lac Z expression is activated after the Cre-mediated excision of the floxed CAT gene. Embryonic hearts were collected at E11.5 and E13.5 and stained in X-Gal staining solution as described previously^24^,^27^.

### Histological analysis

All of the embryos were staged, collected, fixed in 4% paraformaldehyde at 4 °C overnight and embedded in paraffin as described previously^53^,^55^,^56^. The embryos and embryonic hearts were staged by considering the presence of a vaginal plug as day 0.5 after conception. Five millimeter thick sections were mounted on slides and stained with hematoxylin and eosin (H&E staining) according to standard procedures.

### Immunohistochemistry

The cell proliferation rate was determined by bromodeoxyuridine (BrdU) incorporation. E12.5, E13.5 and E14.5 embryos were labeled with BrdU for 2 h by intraperitoneally injecting the pregnant females with BrdU labeling solution (100 μg/g body weight). The cell proliferation index of cardiomyocytes was calculated by dividing the number of BrdU-positive nuclei by the total number of nuclei within a section. Three embryos of each genotype were used to determine the proliferation index for each developmental stage.

The immunohistochemical staining procedure followed the standard streptavidin-peroxidase (SP) protocol. Briefly, embryos were dissected in phosphate buffered saline and embedded in paraffin; the tissue sections were dewaxed and rehydrated. Antigen retrieval was achieved by high-pressure treatment in citrate buffer (pH 6.0) and boiling for 2 min. Endogenous peroxidase and non-specific staining were blocked by with H_2_O_2_ and 1% BSA for 15 min at room temperature, respectively. The sections were then incubated with primary antibodies overnight at 4 °C and subsequently incubated with secondary antibodies. The complex was visualized with DAB and counterstained with hematoxylin. The antibodies and conditions used are summarized in the Supplementary Table S2.

### Statistical analysis

All data were presented as mean ± SD. Statistical analysis was performed using a SPSS 13.0 software package. Values are statistically significant at ^#^*P* < 0.05, *P < 0.01 and **P < 0.001.

## Additional Information

**How to cite this article**: Lin, X. *et al.* Ectopic expression of Cripto-1 in transgenic mouse embryos causes hemorrhages, fatal cardiac defects and embryonic lethality. *Sci. Rep.* **6**, 34501; doi: 10.1038/srep34501 (2016).

## Supplementary Material

Supplementary Movie S1

Supplementary Movie S2

Supplementary Movie S3

Supplementary Movie S4

Supplementary Movie S5

Supplementary Movie S6

Supplementary Movie S7

Supplementary Information

## Figures and Tables

**Table 1:** Genotype frequency of progeny from the mating of RCLG mice and EIIa-Cre mice.

Stage	No. of litters	Genotype (No. of offspring with the indicated genotype)					Total
		G1	G2 (hemorrhage)	G2 (resorbed)	G2 (pale body surface)	G2 (no abnormal appearance)	
E12.5	17	64	0	0	0	89	153
E13.5	15	53	0	0	0	75	128
E14.5	29	103	35	48	37	39	262
E15.5	23	70	13	75	18	27	203
E16.5	16	52	4	49	6	14	125
E17.5	9	25	1	35	1	3	65
Postnatal	20	85	0	0	0	0	85

RCLG transgenic mice used here were heteroygous or homozygous, while EIIa-Cre mice used here were homozygous. The genotypes of offspring were determined by the combinational use of *ex vivo* bioluminescence and fluorescence imaging, as verified by PCR-based genotyping. The genetic background was FVB inbred mice. Genotype-1 (G1): Cre (control embryo), genotype-2 (G2): RCLG/Cre (mutant embryo).
