# Supplementary material for: Ectopic expression of Cripto-1 in transgenic mouse embryos causes hemorrhages, fatal cardiac defects and embryonic lethality
Source: Sci Rep. 2016 Sep 30;6:34501. doi: 10.1038/srep34501 (PMC5043281; doi:10.1038/srep34501)
Supplement: Supplementary Information [file srep34501-s8.pdf]

Ectopic expression of Cripto-1 in transgenic mouse embryos causes hemorrhages, fatal cardiac defects and embryonic lethality

Xiaolin Lin[1,2,4]*, Wentao Zhao[2,5,]*, Junshuang Jia[2,]*, Taoyan Lin[1,2], Gaofang Xiao[2], Shengchun Wang[2], Xia Lin[2], Yu Liu[1], Li Chen[7], Yujuan Qin[2], Jing Li[2], Tingting Zhang[2], Weichao Hao[2], Bangzhu Chen[1], Raoying Xie[2], Yushuang Cheng[2], Kang Xu[6], Kaitai Yao[2], Wenhua Huang[4], Dong Xiao[1,2] & Yan Sun[3]

SUPPLEMENTARY INFORMATION

This supplementary files include:

Supplementary Results

Supplementary Figure S1–S27 (legends included)

Supplementary Tables S1–S3

Supplementary Movie S1-S7

Supplementary references

Supplementary Figure S28 (for the uncropped full-length gels and blots)

Supplementary Results

1. Aortic defects in mutant embryos (RCLG/EIIa-Cre)

The major cardiac defects in the mutant embryos prompted us to further assess the later stages of blood vessel wall morphogenesis and stabilization by examining histological sections of blood vessels from the thoracic and abdominal aortas from mutant embryos.

As shown in Supplementary Figure S21A, Figure S22 and Figure S26, the thoracic aortae from E11.5–14.5 control embryos displayed an intact endothelial cell layer surrounded by a compact tunica media composed of circumferentially elongated vascular smooth muscle cells (VSMCs). In contrast, the thoracic aortae from E11.5–14.5 mutant embryos exhibited an intact endoluminal layer of $CD34^{+}$ endothelial cells surrounded by a poorly organized tunica media composed of α -smooth muscle actin (α -SMA)-expressing VSMCs (Supplementary Figure S21A, Figure S22 and Figure S26).

An examination of histological sections revealed that the E11.5–14.5 mutant embryos possessed thin vessel walls in the thoracic aorta compared with littermate controls, with the vascular wall thinning first becoming apparent at E11.5 and becoming more obvious at E12.5 to E14.5 (Supplementary Figure S21A, B and Figure S22). The thickness of the smooth muscle cell layers of the thoracic aortas of E11.5–14.5 mutant embryos was dramatically reduced (Supplementary Figure S21B-b,d,f and Figure S22). Thus, the thinning of the thoracic aortas of the mutant embryos occurred due to a decrease in the thickness of the smooth muscle cell layers. The mutant embryo VSMCs had prominent nuclei and stained less intensely for α-SMA than the littermate controls (Supplementary Figure S21B-d,f and Figure S22), suggesting reduced smooth muscle differentiation in the thoracic aortae of the mutant embryos. These data suggest that the continued expression of Cripto-1 in the transgenic mouse embryos severely impairs proper vascular development.

Gomori's aldehyde fuchsin staining revealed no significant difference in the elastic fiber thickness of the thoracic aortas of control and mutant embryos at E11.5 (Supplementary Fig. S24) and E12.5 (Supplementary Figure S21C-a,b,c,d), whereas the elastic fibers of the thoracic aortae of E14.5 mutant embryos were significantly thinner than the same fibers in

E14.5 control embryos (Supplementary Figure S21C-e,f,g,h), suggesting abnormal elastic fiber development in the mutant embryos.

The above-mentioned defects were also observed in the smooth muscle cell layers and elastic fibers of the abdominal aortas of E11.5-E13.5 mutant embryos (Supplementary Figure S23 and S25).

Furthermore, there was no significant difference in the endothelial cell layers of the thoracic (Supplementary Figure S26) and abdominal (Supplementary Figure S27) aortas in E11.5 to E14.5 control and mutant embryos.

Thus, the above-mentioned findings indicate that the continued expression of Cripto-1 at E11.5, E12.5, E13.5 or E14.5 causes defects in the smooth muscle cell layers and elastic fibers of the thoracic and abdominal aortas, suggesting possible defects in the patterning of the great vessels and the blood pumping function of the aorta.

Supplementary Figures

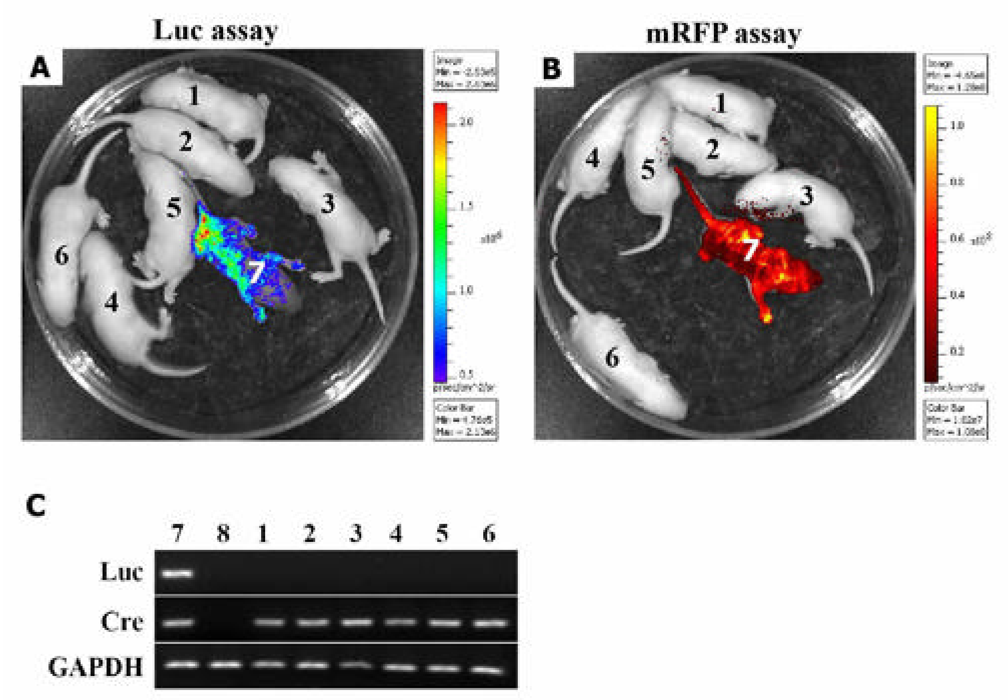

Figure S1. Disappearance of Luc- and mRFP-positive mice from newborn progeny derived from mating heterozygous RCLG mice with homozygous EIIa-Cre mice.

(**A-B**) **In vivo luciferase (A) and mRFP (B) imaging for newborn offspring ($1^{\#}$-$6^{\#}$) derived from mating heterozygous RCLG mice with homozygous EIIa-Cre mice.** Figure 1A fully demonstrates the strategy for conditional expression of Cripto-1 and Luc transgenes using EIIa-Cre mice [in which Cre is under the control of a zygotically expressed (EIIa-Cre) promoter]. mRFP and the activation of Luc expression in a diffuse pattern in newborn RCLG/EIIa-Cre double transgenic mice were detected via the Xenogen IVIS Lumina Ⅱ Imaging System. RLG/EIIa-Cre double transgenic mouse (i.e., referred as to $7^{\#}$) is used as a positive control. Other details for RLG/EIIa-Cre mice as in Supplementary Figure S2.

(C) PCR-based genotyping for Cre and Luc transgenes in RCLG/EIIa-Cre transgenic mice.
Both six mRFP- and Luc-negative mice (i.e., $1^{\#}$-$6^{\#}$), and one mRFP- and Luc-positive mouse (i.e., $7^{\#}$) were individually analyzed by PCR for the genomic integration of Cre and Luc transgenes with tail biopsy-derived DNA from mice ($1^{\#}$-$7^{\#}$). PCR products were amplified by the primer pair P1/P2 (specific for Luc) and by the primer pair P3/P4 (specific for Cre), respectively. P1: 5'-CATCGTTGACCGCCTGAAGT-3', P2: 5'-ACTCCTCCGCGCAACTTTTT-3'; P3: 5'-GAACCTGATGGACATGTTCAGG-3', P4: 5'-AGTGCGTTCGAACGCTAGAGCCTGT-3'. Lane 1-7: $1^{\#}$-$7^{\#}$ mice; lane 8: negative control using genomic DNA from WT mouse as template. Data are representative of three independent PCR experiments that yield similar results. The cropped gels are used in Supplementary Figure S1C, and the full-length gel images are available in Supplementary Figure S28. The gels have been run under the same experimental conditions.

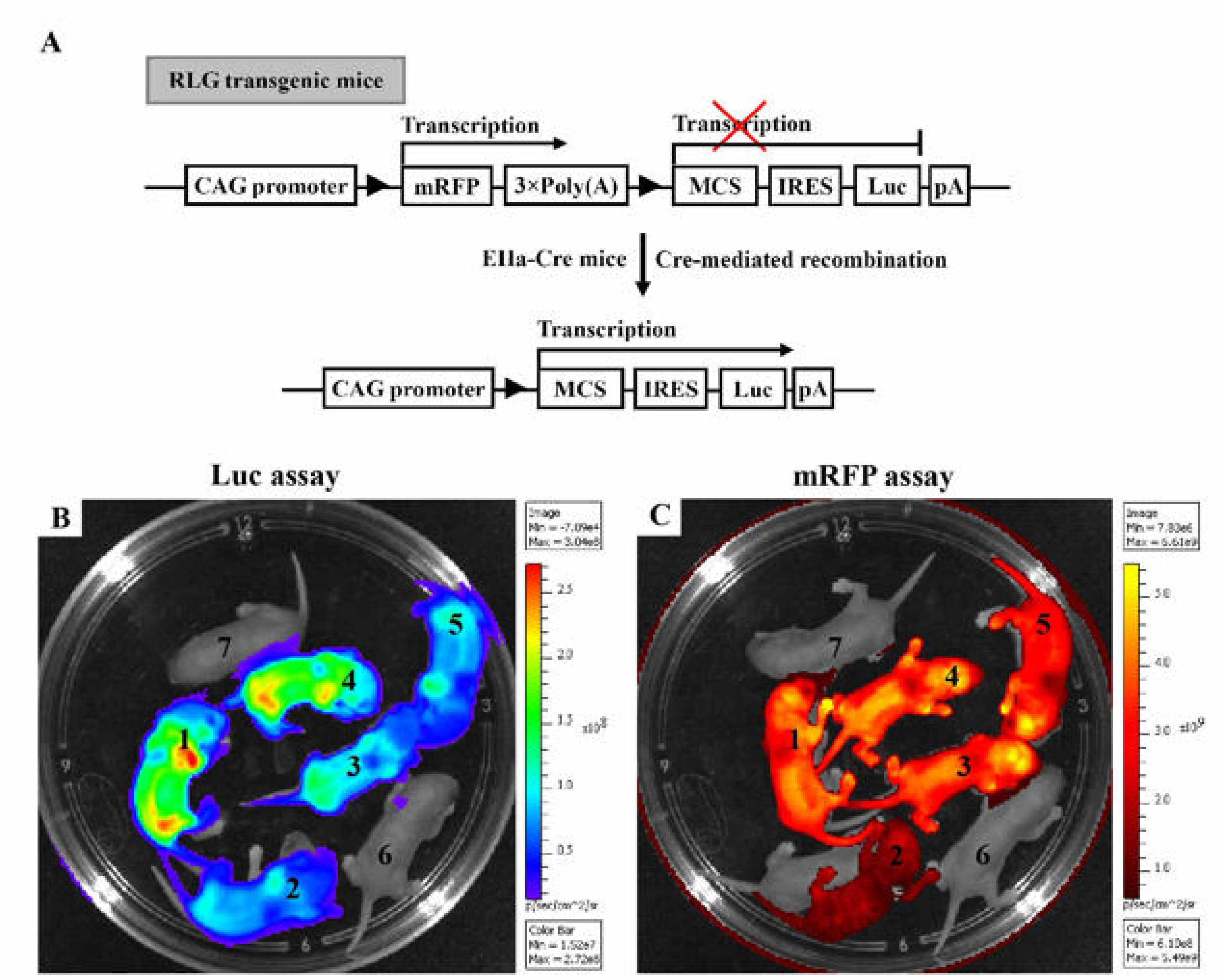

Figure S2. Activation of Luc expression in all of newborn offspring from mating homozygous RLG transgenic mice with homozygous EIIa-Cre mice.

(**A**) **Conditional expression of Luc mediated by Cre/*lox* P system.**

Homozygous RLG transgenic mice harboring CAG-RLG transgene isolated from the vector of pCAG-RLG[1,2] were generated by us. In the absence of Cre-mediated recombination, only mRFP will be transcribed, while Luc gene expression is prevented by STOP sequence flanked by *lox* P sites. When Cre-mediated recombination occurs, the floxed mRFP + 3×PolyA is excised, and Luc expression is activated in a diffuse pattern in RLG/EIIa-Cre double transgenic mice (Supplementary Figure S2B). mRFP expression and the activation of Luc expression were assayed in newborn RLG/EIIa-Cre double transgenic mice by the Xenogen IVIS Lumina II Imaging System. Other details as in Figure 1A. Abbreviations: CAG promoter： CMV early enhancer/chicken β-actin promoter； mRFP: monomeric red fluorescent protein; MCS: multiple cloning site; Luc: firefly luciferase； pA: polyadenylation signal; ▶ : *lox* P site.

(B-C) Activation of Luc expression in a diffuse pattern in all of newborn RLG/EIIa-Cre double transgenic mice

As the efficiency of Cre-mediated recombination in RLG/EIIa-Cre double transgenic mice can not reach 100%, some cells in different organs of RLG/EIIa-Cre double transgenic mice still harbored mRFP gene. Therefore, mRFP expression was still detectable in whole-body (Supplementary Figure S2C) of RLG/EIIa-Cre double transgenic mice.

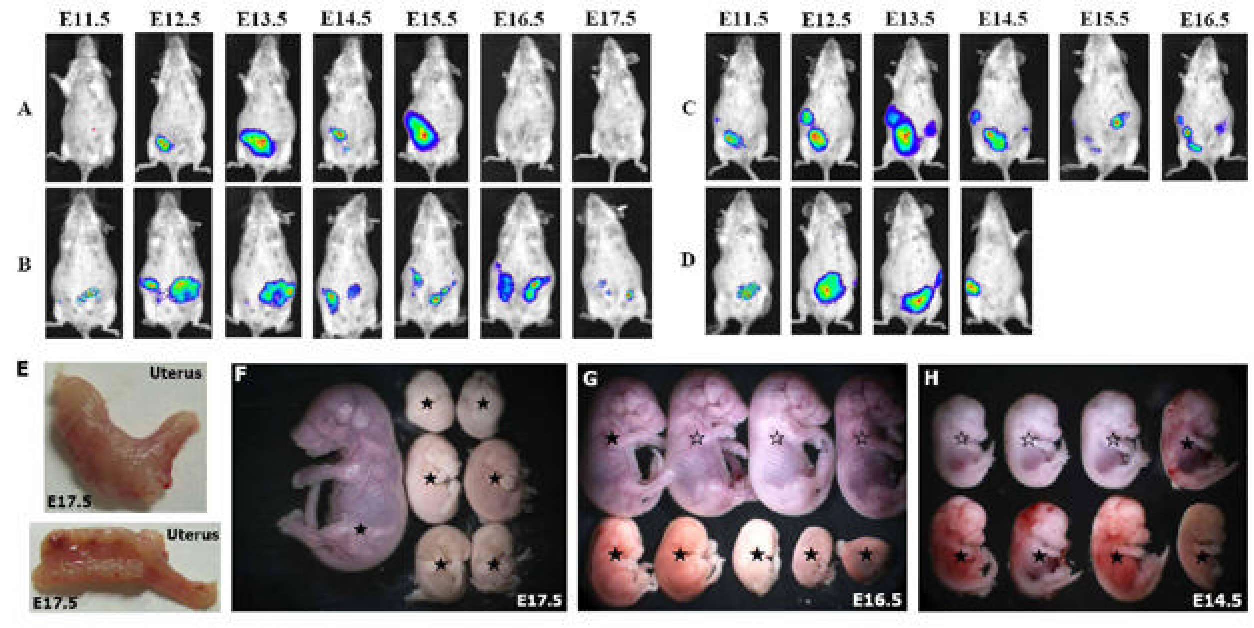

Figure S3. Time point of embryonic lethality determined by in vivo bioluminescence imaging.

(A-D) In vivo bioluminescence imaging for pregnant females.

The pregnant females were derived from the following intercrossings (i.e., A, B, C and D) of RCLG mice and EIIa-Cre mice. In vivo bioluminescence imaging for pregnant females at indicated time was performed to detect Luc-positive embryos. **A**: homozygous RCLG mouse (ID:1248; ♂) × homozygous EIIa-Cre mouse (ID:693; ♀); **B**: homozygous RCLG mouse (ID:1248; ♂) × homozygous EIIa-Cre mouse (ID:1343; ♀); **C**: heteroygous RCLG mouse (ID:1267; ♀) × homozygous EIIa-Cre mouse (ID:956; ♂); **D**: heteroygous RCLG mouse (ID:1274; ♀) × homozygous EIIa-Cre mouse (ID:955; ♂).

(E-H) Embryo macroscopic observation.

After whole-body bioluminescence imaging for pregnant females, embryos were immediately harvested from pregnant females (i.e., 693#, 1343#, 1267# and 1274#) at indicated time points, respectively, and subsequently imaged using the IVIS system for Luc and mRFP assay (Supplementary Figure S4), immediately followed by whole-mount view of the dissected embryos by using stereo fluorescence microscope (Nikon, AZ100) (Supplementary Figure S3E,F,G,H). E, F, G and H indicate embryos harvested from pregnant females, such as 693# (at E17.5), 1343# (at E17.5), 1267# (at E16.5) and 1274# (at E14.5), respectively. Genotype-1: Cre (marked by ☆) (control embryo), and Genotype-2: RCLG/Cre (marked by ★) (mutant embryo). The genotype of all embryos from pregnant females (ID: 693# and 1343#) is RCLG/Cre, while these embryos from pregnant females (ID: 1267# and 1274#) have two genotypes, including Cre (Genotype-1) and RCLG/Cre (Genotype-2).

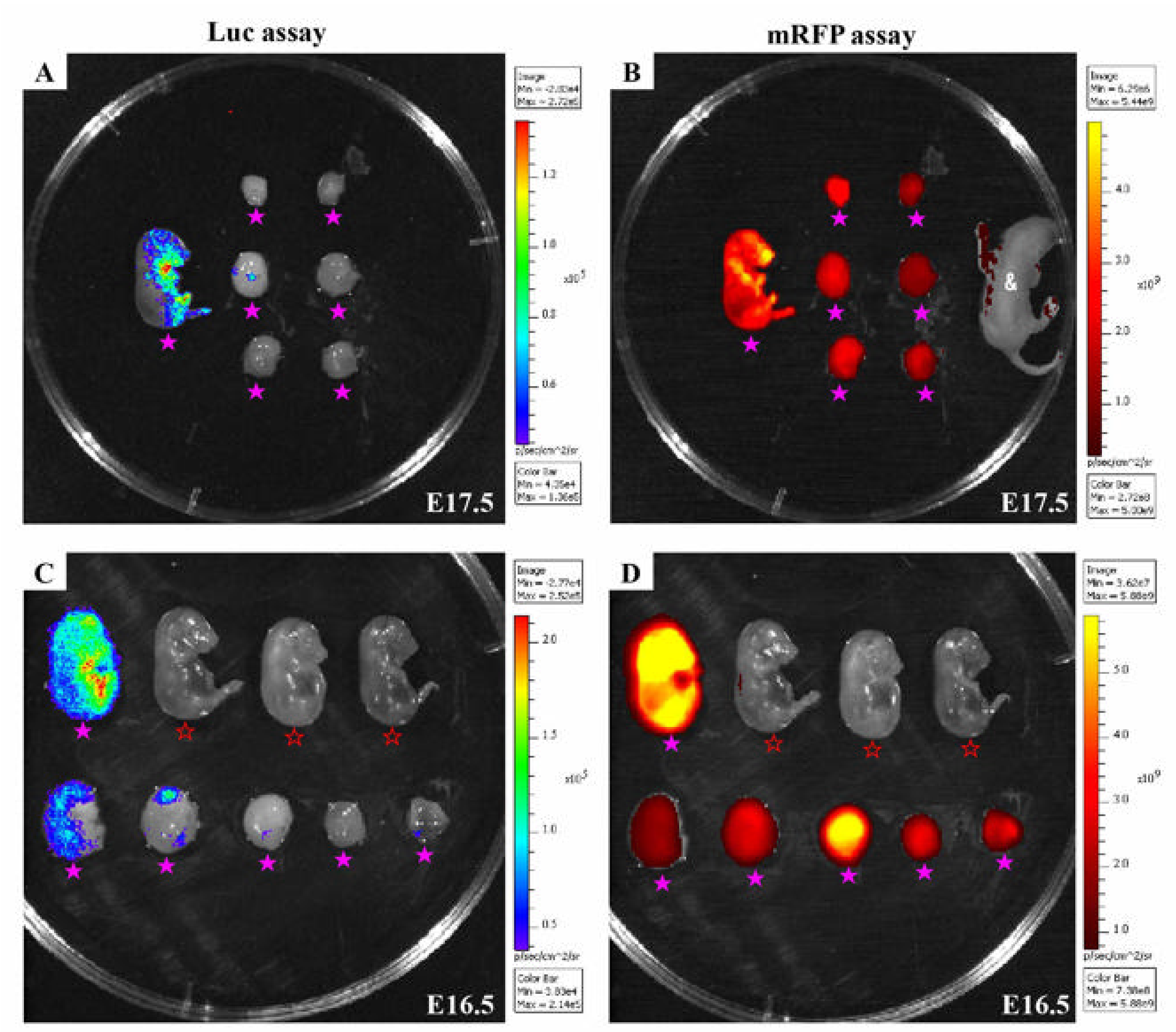

Figure S4. Luc- and mRFP-positive embryos detected by ex vivo bioluminescence and fluorescence imaging.

For ex vivo bioluminescence and fluorescence imaging of isolated embryos at indicated time points, pregnant females were injected with D-luciferin and subsequently anesthetized by isoflurane. After the photon accumulation at 1-min intervals, examined by in vivo imaging, reached a maximum level, the pregnant females were sacrificed, and then the embryos were rapidly excised, followed by the dissected embryos imaged by the IVIS system for detecting Luc and mRFP signal. Genotype-1: Cre (marked by ☆) (control embryo), and Genotype-2: RCLG/Cre (marked by ★) (mutant embryo). Additionally, wild-type newborn mouse (used as negative control during fluorescence imaging) was marked by "&". Genotyping results derived from ex vivo Luc and mRFP imaging were confirmed by PCR-based genotyping. Please see Supplementary Figure S3 for other details.

(**A-B**) **Ex vivo luciferase (A) and mRFP (B) imaging for embryos (E17.5) harvested from pregnant female (ID:1343) (see Supplementary Figure S3 for details).**

The pregnant females (ID:1343) were derived from intercrossing of homozygous RCLG mouse (ID:1248; ♂) and homozygous EIIa-Cre mouse (ID:1343; ♀).

(**C-D**) **Ex vivo luciferase (C) and mRFP (D) imaging for embryos (E16.5) harvested from pregnant female (ID:1267) (see Supplementary Figure S3 for details).**

The pregnant females (ID:1267) were derived from intercrossing of heteroygous RCLG mouse (ID:1267; ♀) and homozygous EIIa-Cre mouse (ID:956; ♂). Moreover, Ex vivo luciferase and mRFP imaging for embryos (E14.5) harvested from pregnant female (ID:1274) which was derived from intercrossing of heteroygous RCLG mouse (ID:1274; ♀) and homozygous EIIa-Cre mouse (ID:955; ♂) was also performed (data not shown).

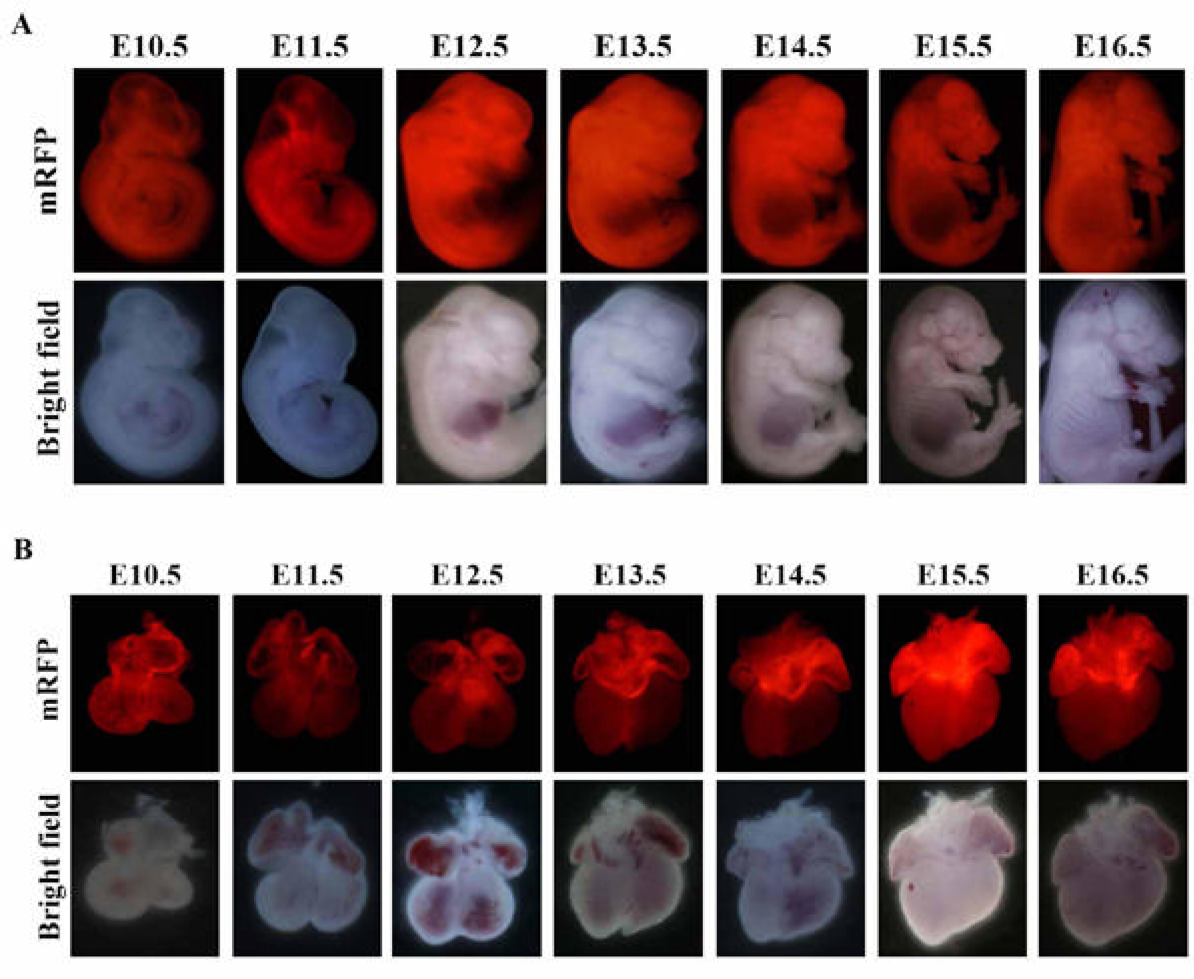

Figure S5. mRFP expression in RCLG transgenic embryos and their hearts.

The embryos and embryonic hearts at E10.5-E16.5 were produced by mating heterozygous RCLG transgenic mice with wild-type mice. mRFP expression in RCLG transgenic embryos and their hearts was assayed under stereo fluorescent microscope (Nikon, AZ100), while mRFP expression in non-transgenic embryos and their hearts (used as negative control) was also assayed under stereo fluorescent microscope (Nikon, AZ100) (data not shown).

(A) mRFP expression in RCLG transgenic embryos.

(B) mRFP expression in the heart of RCLG transgenic embryos.

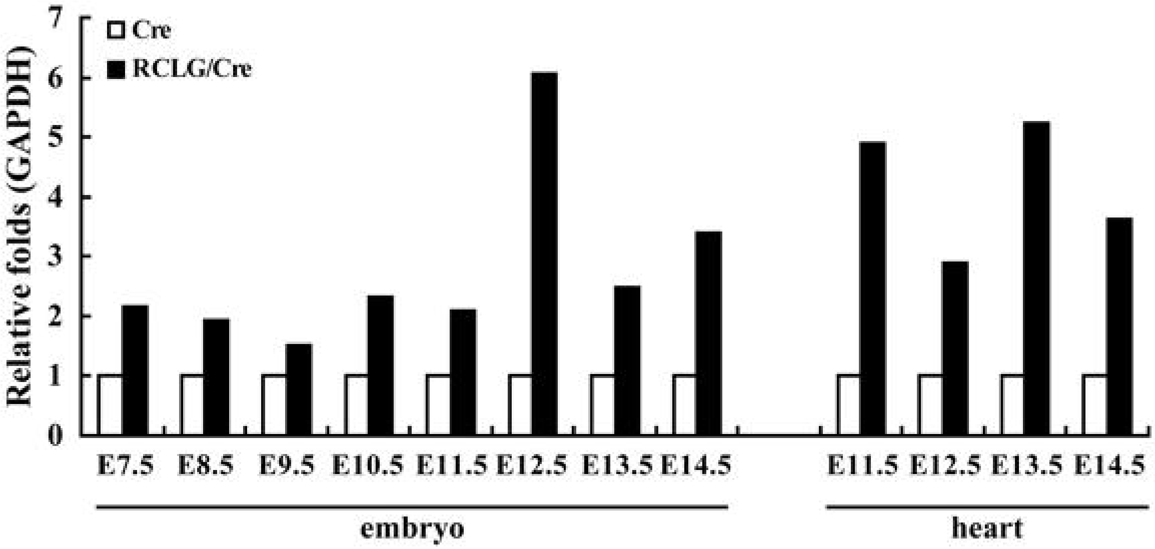

Figure S6 (Extended data Fig. 1G). The quantification of Cripto-1 expression in RCLG/EIIa-Cre transgenic embryos and their hearts.

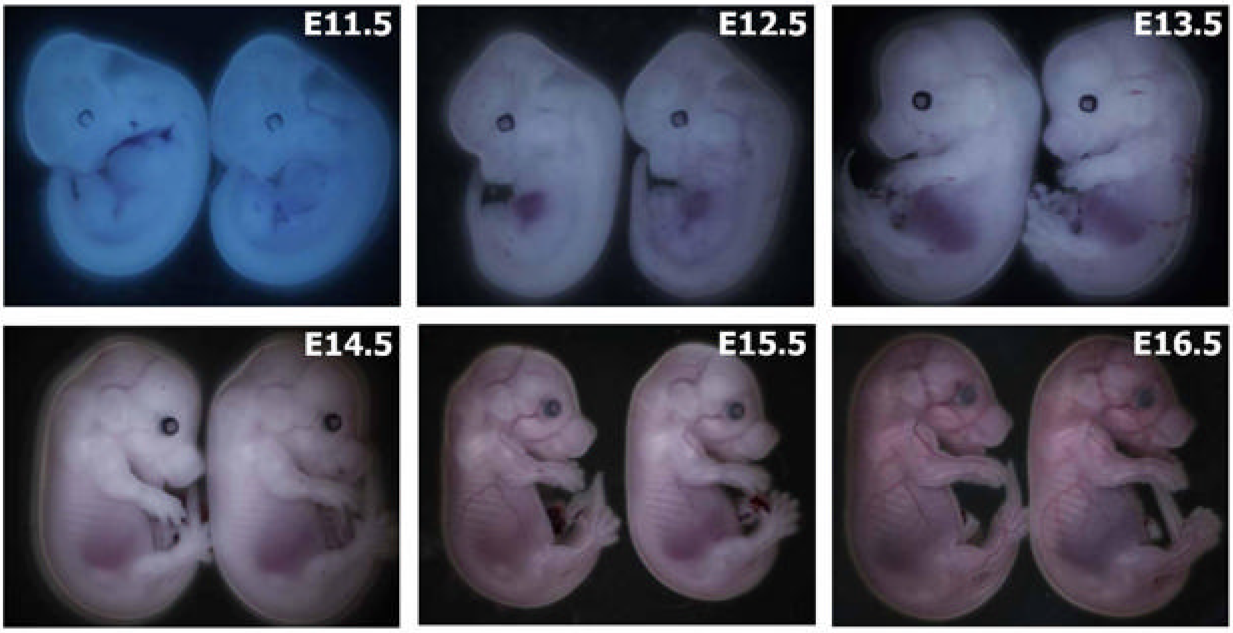

Figure S7. Gross morphology of representative RLG/EIIa-Cre transgenic embryos.

These embryos were collected at the indicated time points from pregnant females which were derived from intercrossing of heteroygous RLG mice (see Supplementary Figure S2 for details) and homozygous EIIa-Cre mice. Genotype of left embryo of each picture is RLG/EIIa-Cre, while genotype of right embryo of each picture is EIIa-Cre.

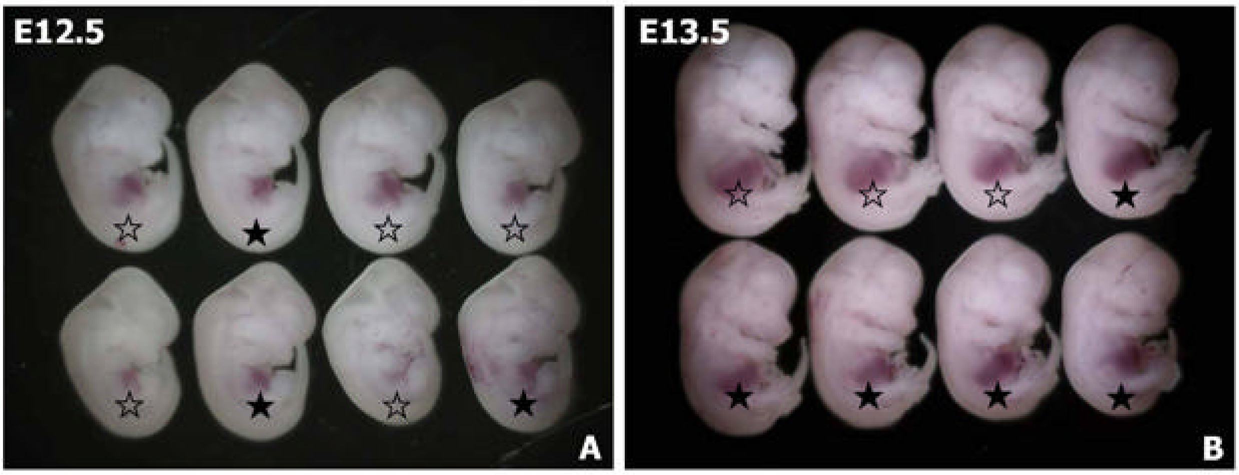

Figure S8. Whole-mount view of mutant and control embryos at E12.5 and E13.5.

These embryos were collected at E12.5 (A) and E13.5 (B) from two pregnant females (i.e., A and B), respectively, while two pregnant females were derived from intercrossing of heteroygous RCLG mice and homozygous EIIa-Cre mice.

Genotype-1: Cre (marked by ☆) (control embryo), and Genotype-2: RCLG/Cre (marked by ★) (mutant embryo).

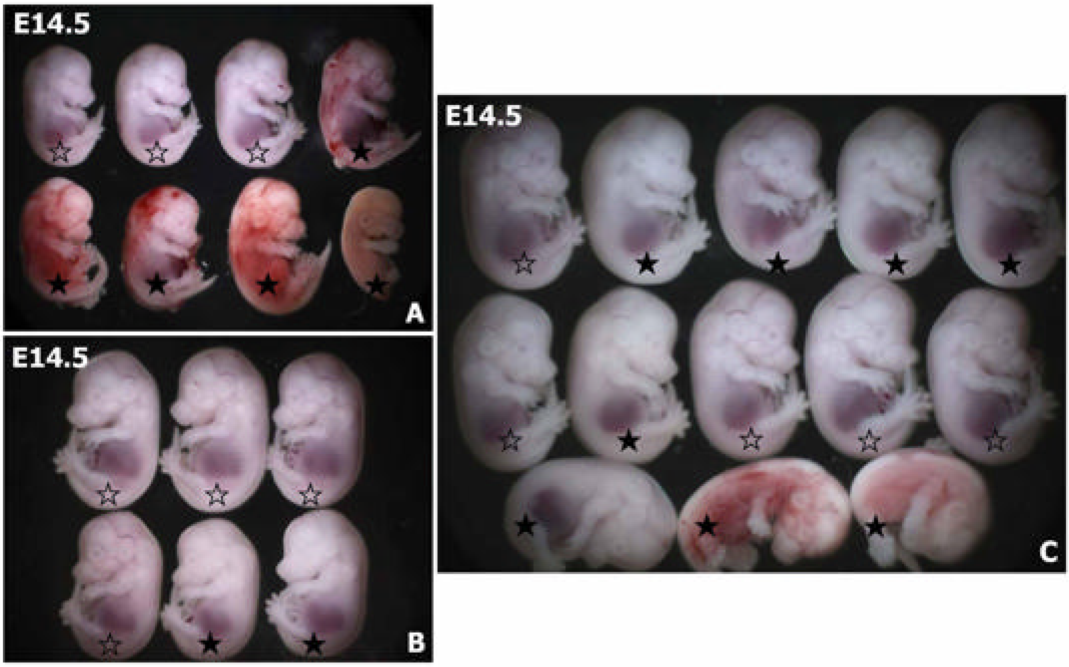

Figure S9. Whole-mount view of mutant and control embryos at E14.5.

These embryos were harvested at E14.5 from three pregnant females (i.e., A, B and C), respectively, while three pregnant females were derived from intercrossing of heteroygous RCLG mice and homozygous EIIa-Cre mice. Other details as in Supplementary Figure S8.

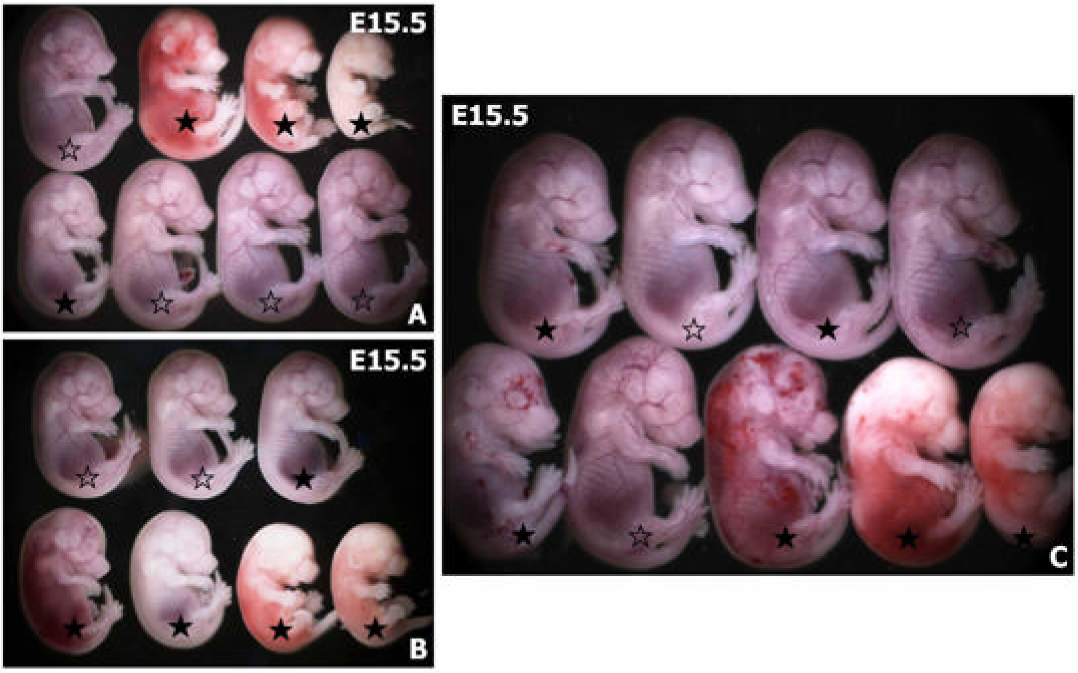

Figure S10. Whole-mount view of mutant and control embryos at E15.5.
These embryos were collected at E15.5 from three pregnant females (i.e., A, B and C), respectively, while three pregnant females were derived from intercrossing of heteroygous RCLG mice and homozygous EIIa-Cre mice. Other details as in Supplementary Figure S8.

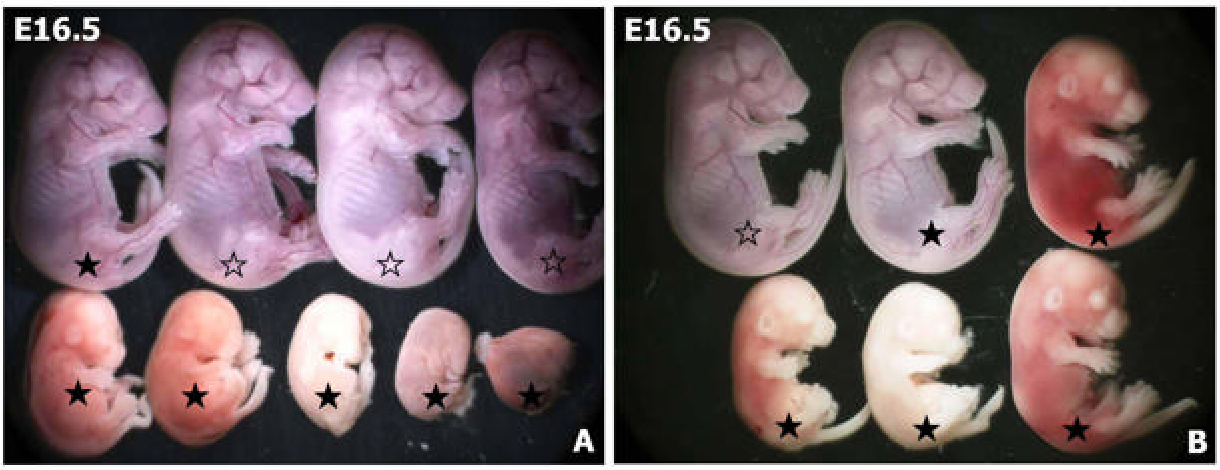

Figure S11. Whole-mount view of mutant and control embryos at E16.5.
These embryos were collected at E16.5 from two pregnant females (i.e., A and B), respectively, while two pregnant females were derived from intercrossing of heteroygous RCLG mice and homozygous EIIa-Cre mice. Other details as in Supplementary Figure S8.

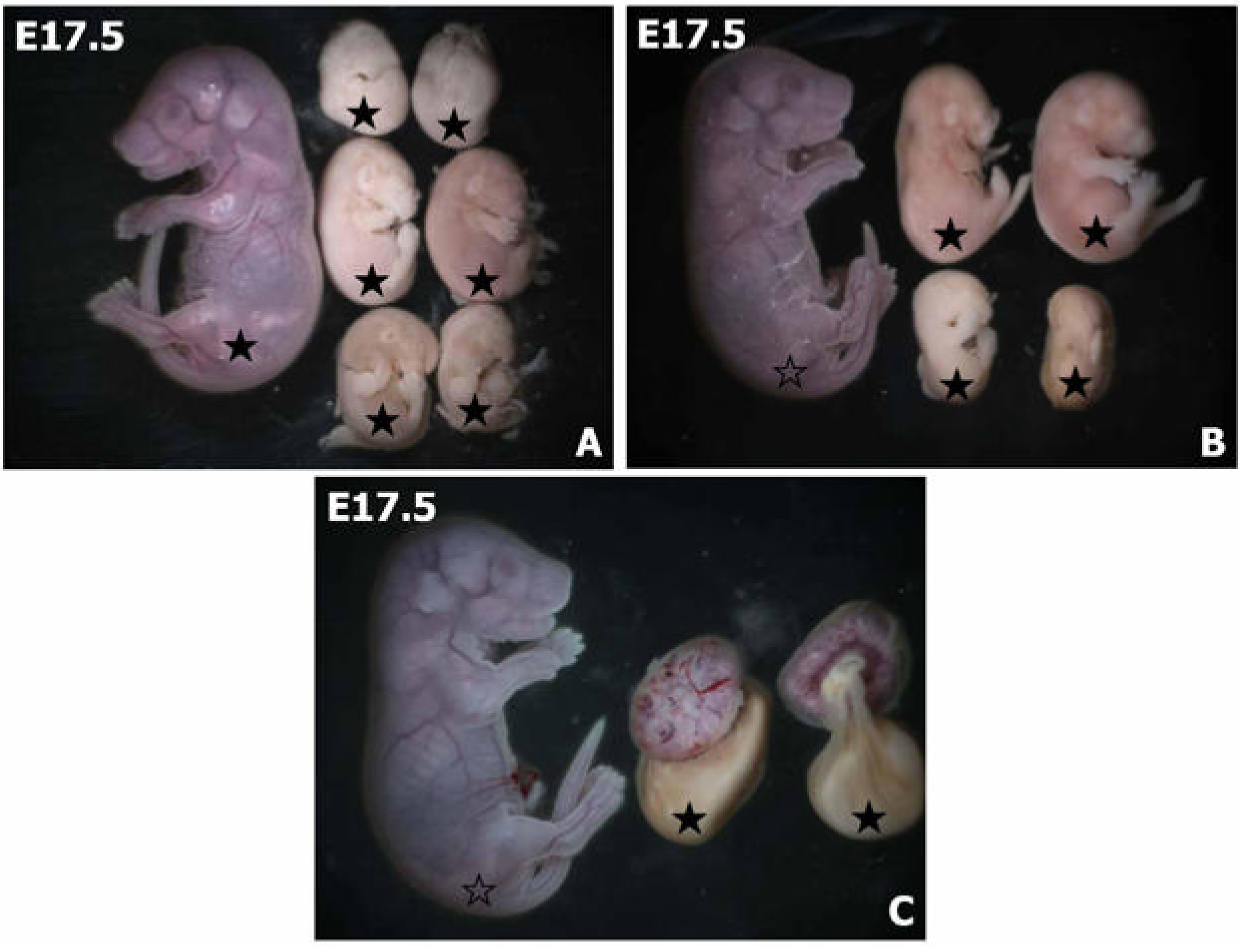

Figure S12. Whole-mount view of mutant and control embryos at E17.5.
These embryos were collected at E17.5 from three pregnant females (i.e., A, B and C), respectively, while three pregnant females were derived from intercrossing of heteroygous RCLG mice and homozygous EIIa-Cre mice. Other details as in Supplementary Figure S8.

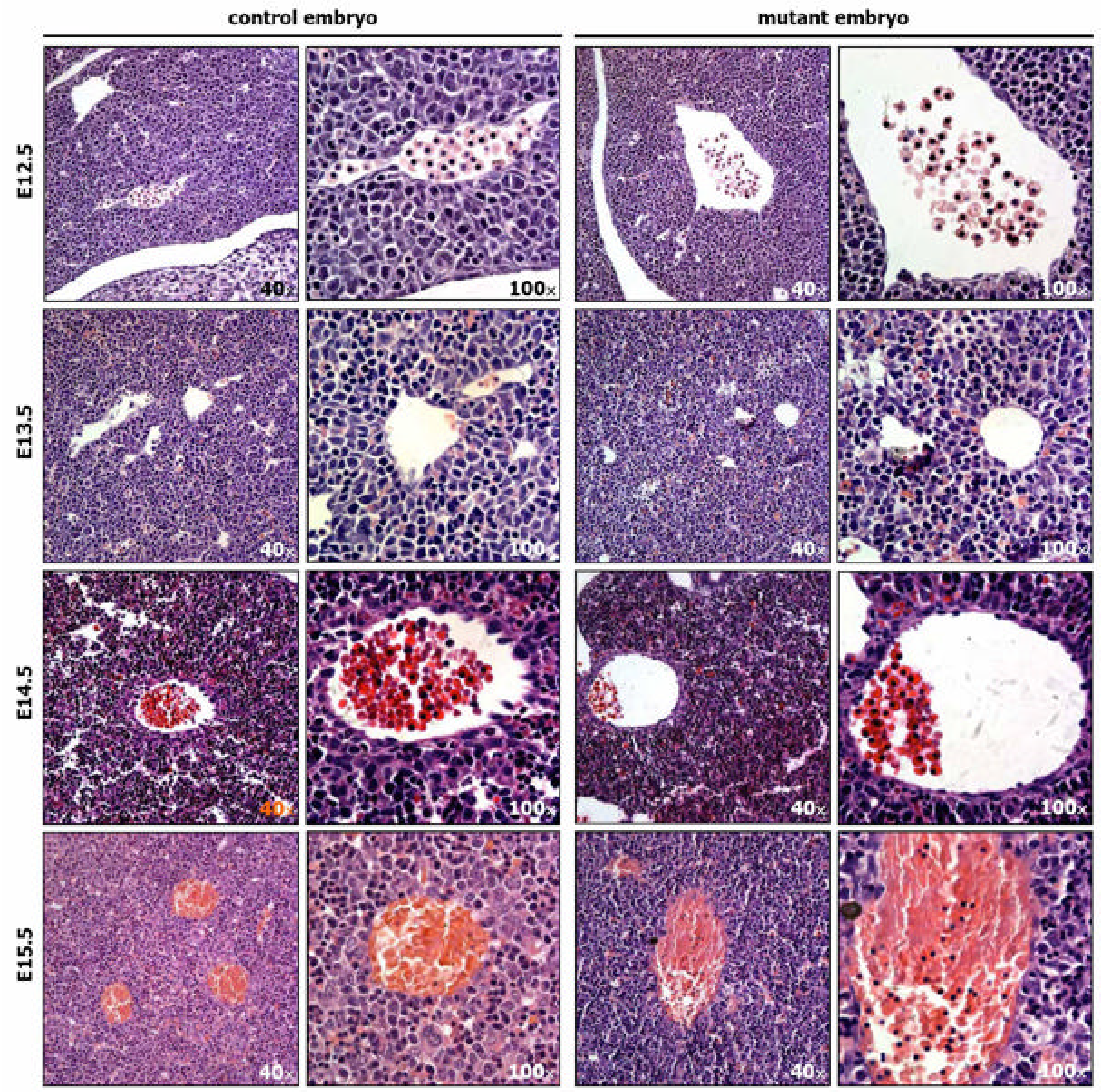

Figure S13. Histological analysis of the embryonic liver of control and mutant embryos between E12.5 and E15.5.

H&E staining of sagittal section of control (EIIa-Cre) and mutant (RCLG/EIIa-Cre) embryos at E12.5 (Supplementary Figure S15), E13.5 (Fig. 2D), E14.5 (Fig. 2B) and E15.5 (Fig. 2B) was performed to fully demonstrate that hemorrhagic changes were only observed on body surface capillaries of E14.5 and E15.5 mutant embryos (Fig. 2B-C), but not in internal organs of mutant embryos, which were verified in sections of liver (Supplementary Figure S13) and other organs (data not shown).

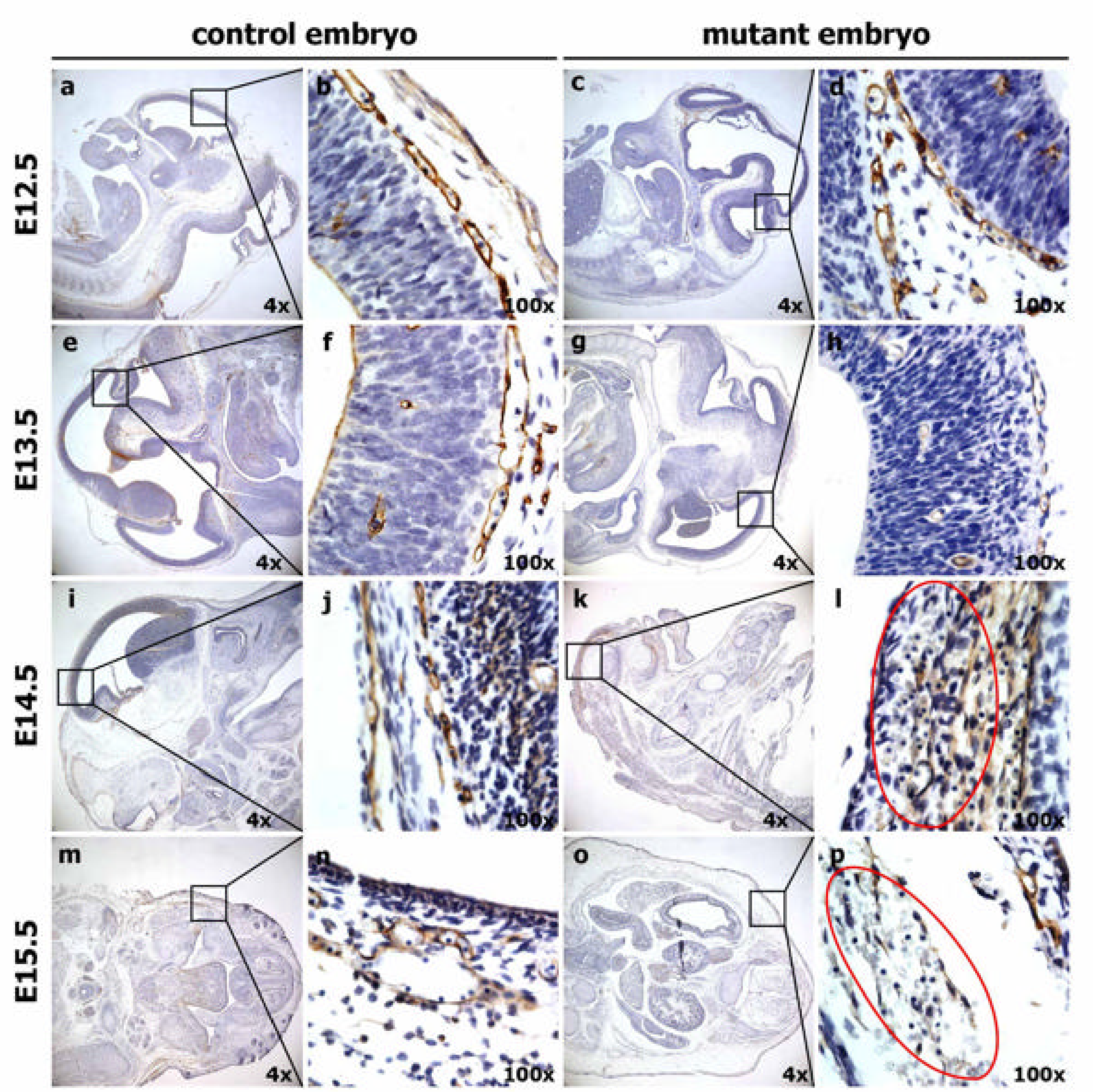

Figure S14 (Extended Data Fig. 2). Immunohistochemical (IHC) staining (for CD34) of the sections from control (EIIa-Cre) and mutant (RCLG/EIIa-Cre) embryos at E12.5-E15.5.

The sections of control and mutant embryos at E12.5 (indicated in Fig. 2A), E13.5 (indicated in Fig. 2D), E14.5 (indicated in Fig. 2B) and E15.5 (Fig. 2B) were stained with an anti-CD34 antibody to reveal endothelial cell layer of blood vessel.

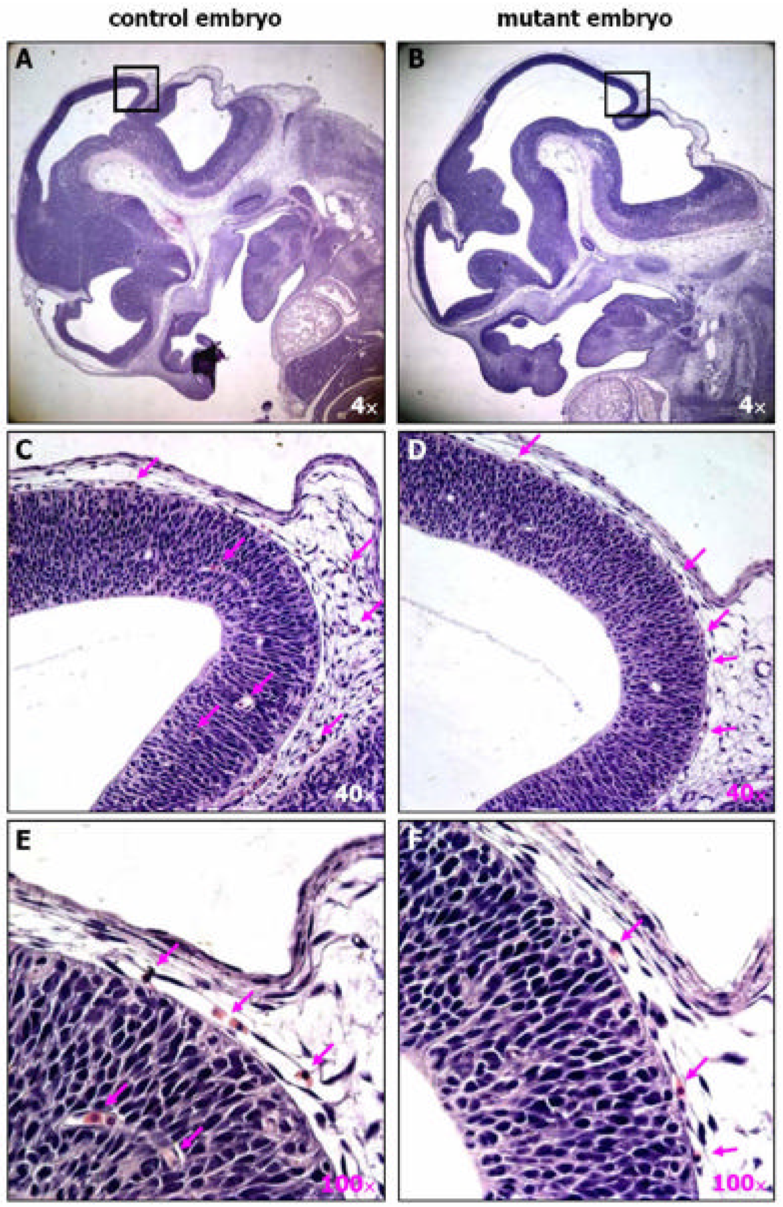

Figure S15. Histological analysis of control (EIIa-Cre) and mutant (RCLG/EIIa-Cre) embryos at E12.5.

No significant difference was found in body surface between E12.5 control and mutant embryos indicated in Fig. 2A and Supplementary Figure S8. H&E staining of sagittal section of control and mutant embryos at E12.5 (Fig. 2A) was performed to determine whether or not the development of body surface capillaries of E12.5 mutant embryos is normal. In Supplementary Figure S15, (C) and (D) are higher magnifications of the black boxes indicated in (A) and (B),respectively, while (E) and (F) are higher magnifications of (C) and (D), separately. Pink arrows indicate normal capillaries (C-F) with erythrocytes, which are nucleated at this stage of development in body surface of control and mutant embryos.

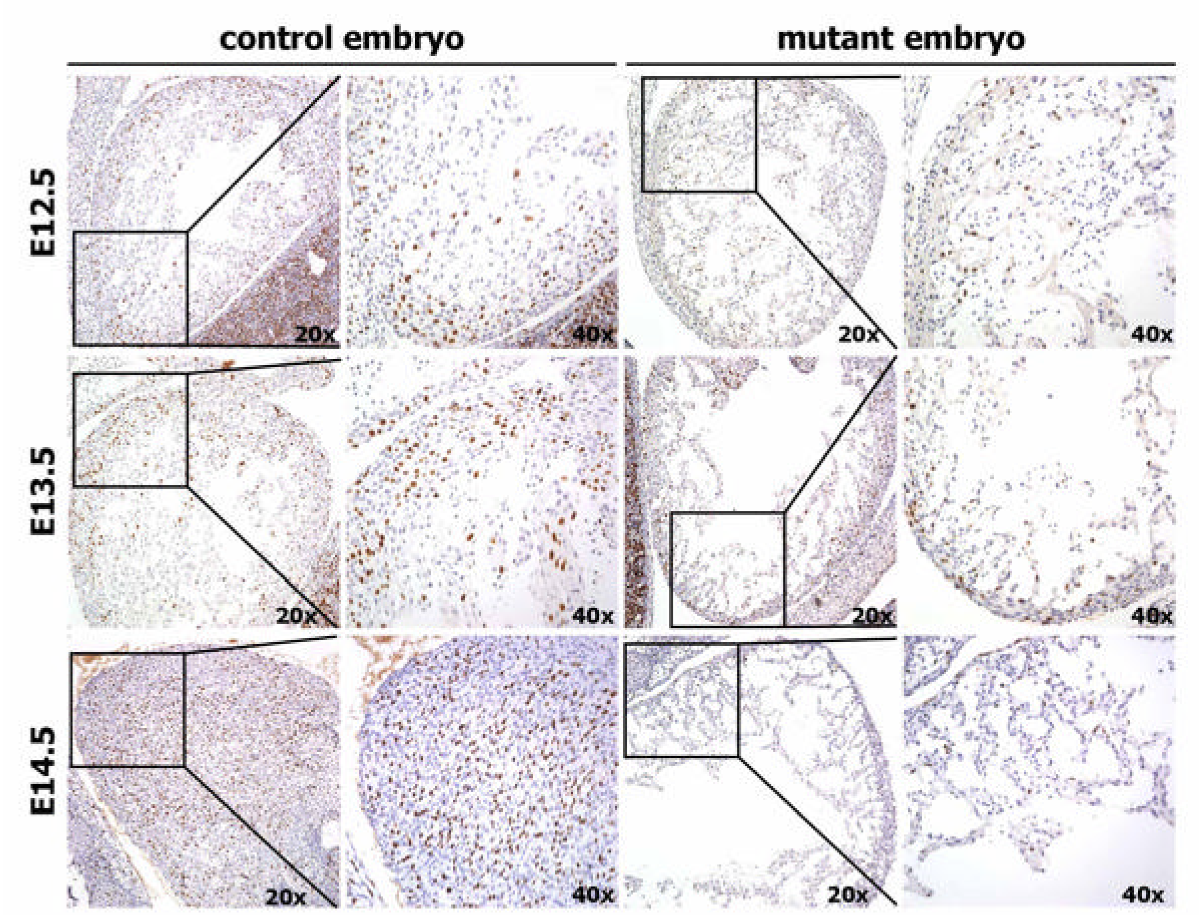

Figure S16 (Extended Data Fig. 4B). Representative BrdU staining of histological sections of hearts from control and mutant embryos at E12.5-E14.5.

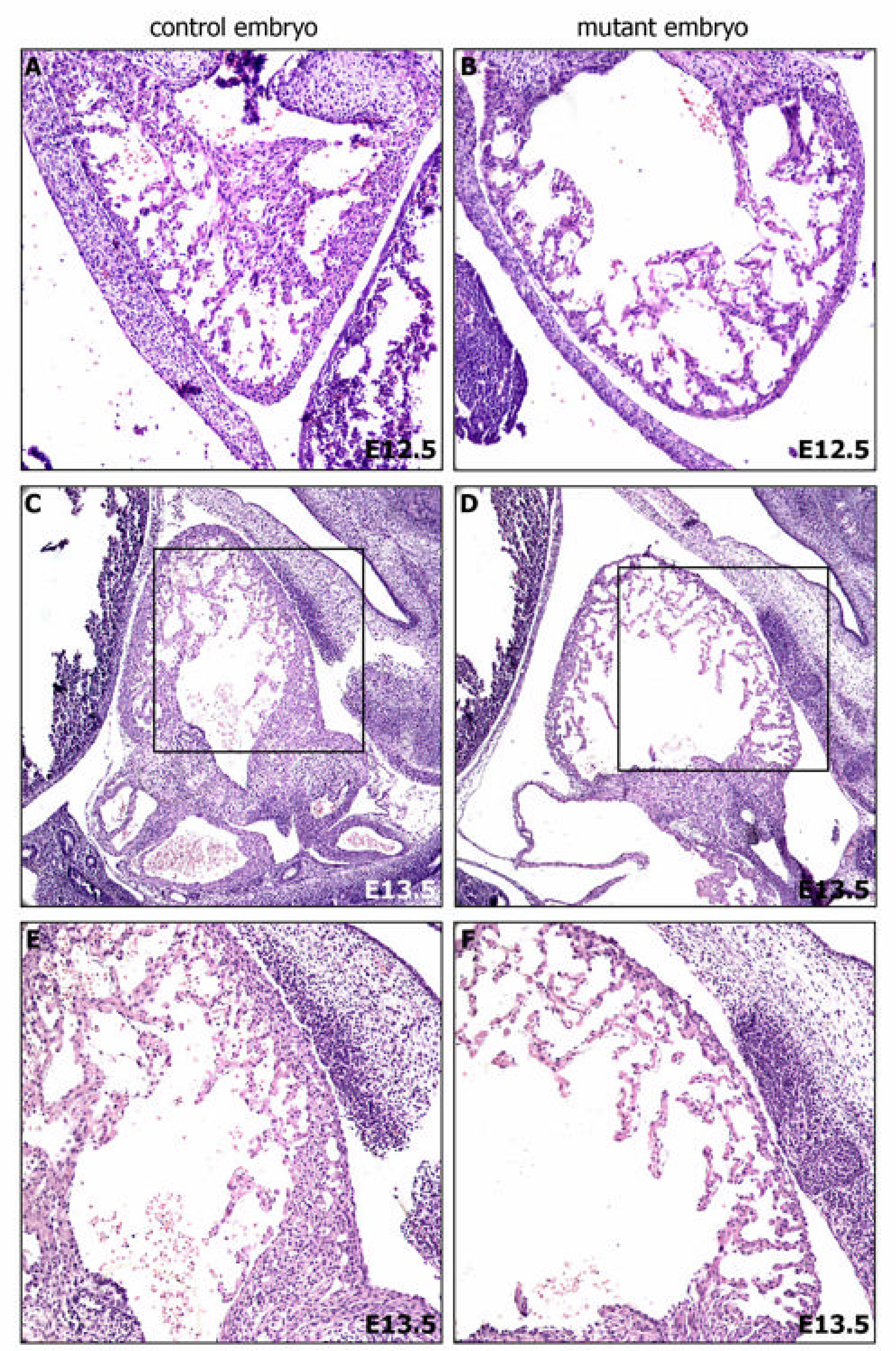

Figure S17. Histological analysis of embryonic hearts of control (EIIa-Cre). (A, C and E) and mutant (RCLG/EIIa-Cre) (B, D and F) embryos at E12.5 and E13.5. (E) and (F) are higher magnifications of the regions indicated in (C) and (D), respectively.

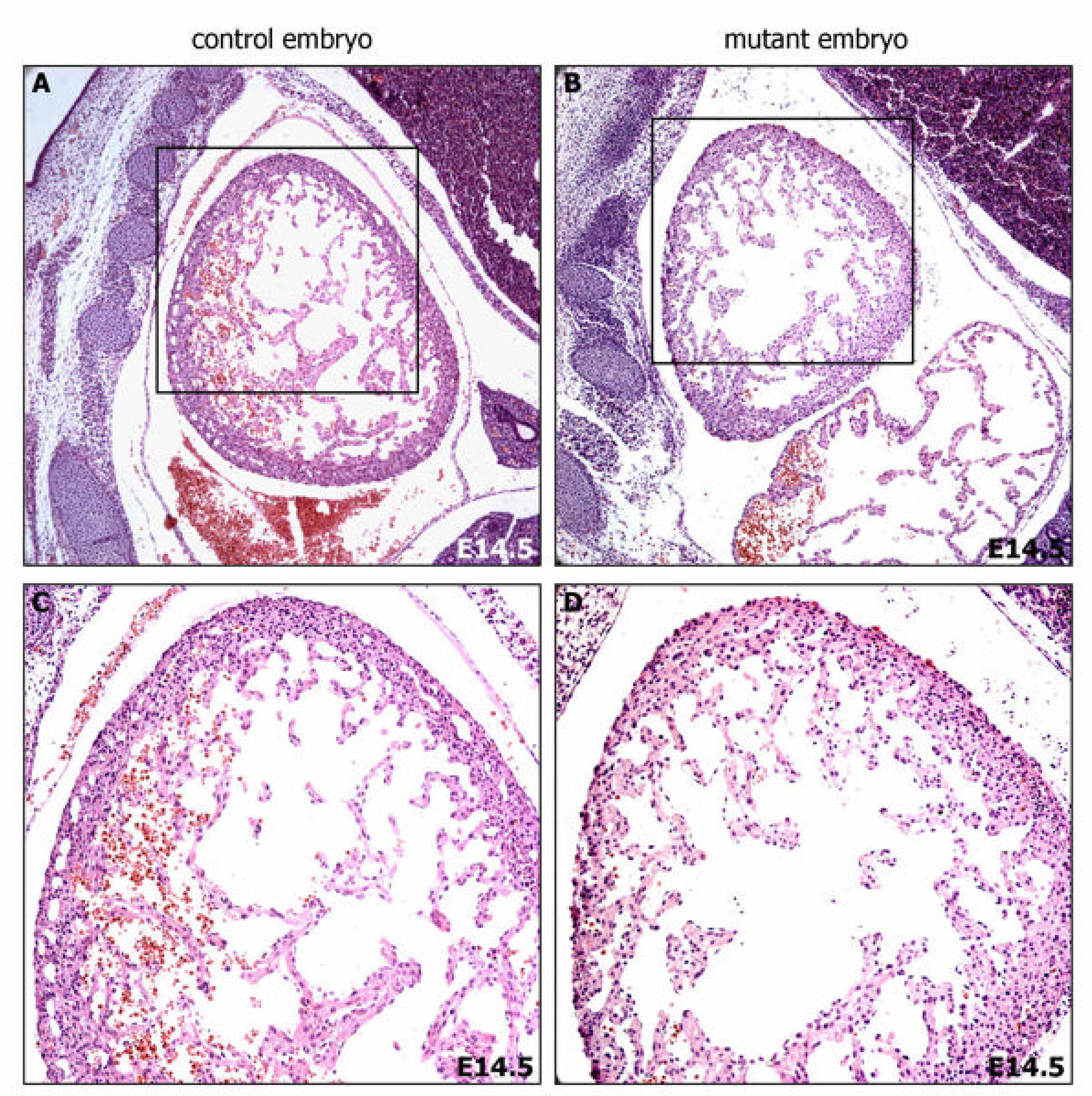

Figure S18. Coronary vascular defects in E14.5 mutant embryo.

H&E staining of embryonic heart sections of control (EIIa-Cre) (A and C) and mutant (RCLG/EIIa-Cre) (B and D) embryos at E14.5 was performed. (C) and (D) are higher magnifications of the regions indicated in (A) and (B), respectively.

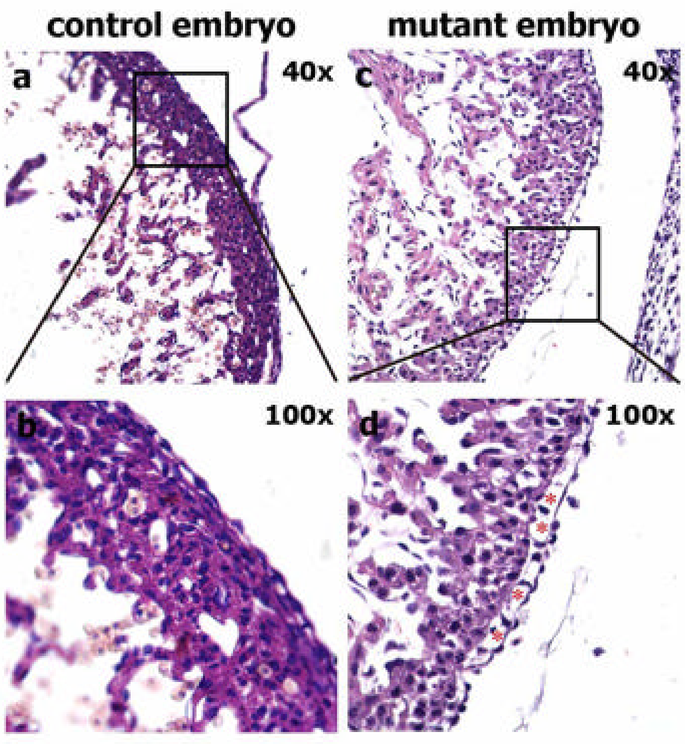

Figure S19. Epicardial blisters in mutant mouse embryonic hearts.

Histological sections of E13.5 mouse embryonic hearts show the epicardial morphology of control embryonic hearts (a,b) and mutant embryonic hearts (c,d). The lower images (b) and (d) are higher magnifications of the rectangular regions indicated in images (a) and (c), respectively. Note the striking epicardial blisters seen in the mutant mouse embryonic heart (asterisks, d).

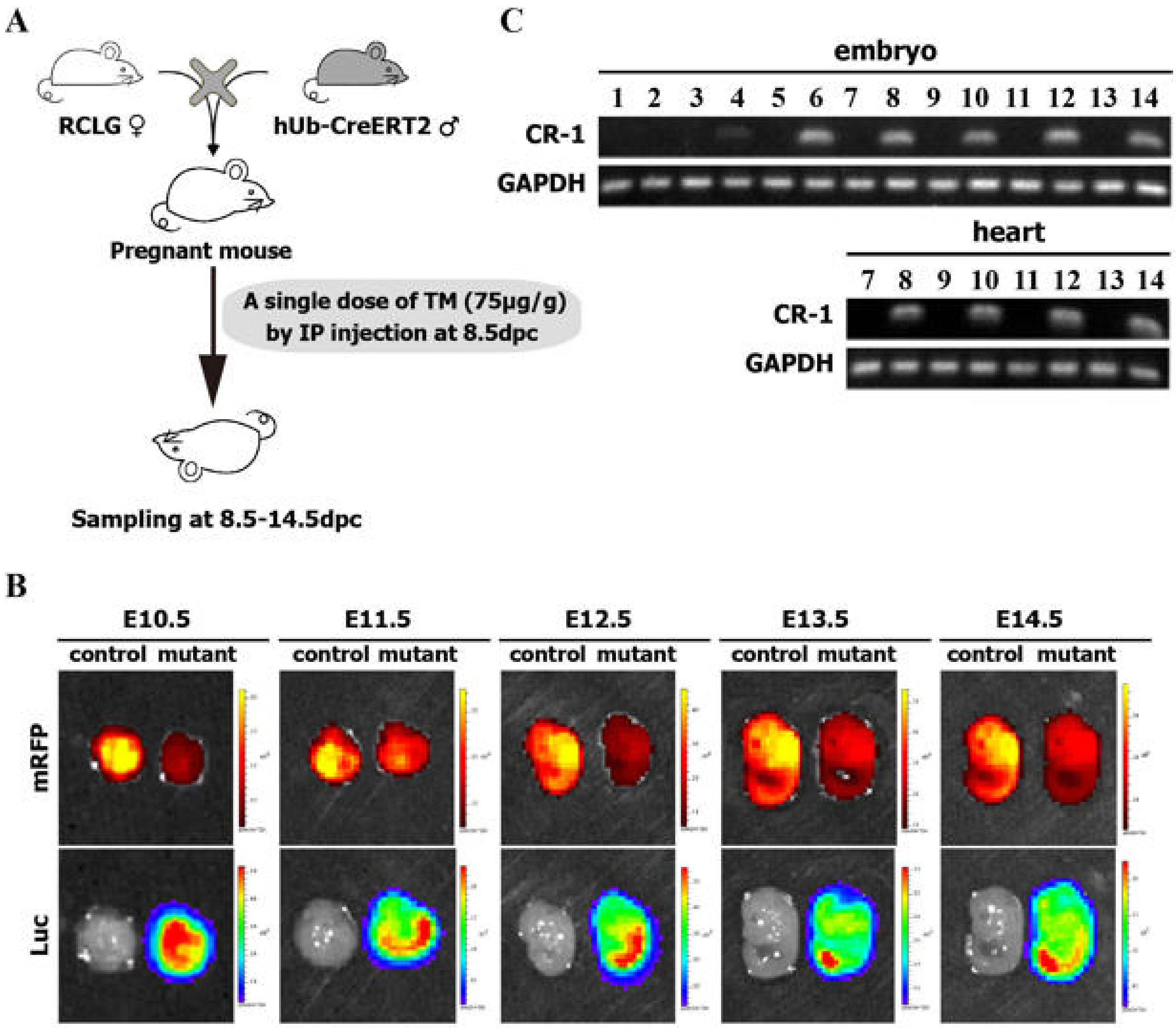

Figure S20. Temporally regulated Cripto-1 and Luc activation in embryos by hUb-CreERT2 mice.

(**A**) The strategy for the temporally regulated activation of the Cripto-1 and Luc transgene expression in embryos is illustrated.

The previous reports fully demonstrated that tamoxifen (TM)-inducible recombination can be used to effectively modify gene function in mouse embryos and most embryonic organs (i.e., embryonic heart)[3,4]. As shown in Supplementary Figure S20A, the homozygous RCLG transgenic mice are mated with the heterozygous hUb-CreERT2 mice [129S.Cg-Tg(UBC-cre/ESR1)1Ejb/J; Stock Number: 007179][5] to generate embryos with two genotypes, including RCLG and RCLG/hUb-CreERT2. To realize the activation of Cripto-1 and Luc transgene expression at the later stages of embryonic development, pregnant mice were treated with tamoxifen (TM) (Sigma-Aldrich; Cat. No. T-5648) by a single, intraperitoneal (IP) injection at 75 μ g/g (Supplementary Figure S20A) at 8.5 days

postcoitum (dpc), followed by sampling at 8.5-14.5dpc. TM was dissolved in corn oil at a concentration of 5 mg/mL.

(B) Activation of Luc expression in RCLG/hUb-CreERT2 double transgenic embryos between E10.5 and E14.5 after a single IP injection of TM at 8.5dpc.

(C) RT-PCR analysis using human-specific primer for human Cripto-1 transgene expression in RCLG/hUb-CreERT2 double transgenic embryos & embryonic hearts.

Lane 1: E8.5+RCLG; lane 2: E8.5+RCLG/Cre; lane 3: E9.5+RCLG; lane 4: E9.5+RCLG/Cre; lane 5: E10.5+RCLG; lane 6: E10.5+RCLG/Cre; lane 7: E11.5+RCLG; lane 8: E11.5+ RCLG/Cre; lane 9: E12.5+RCLG; lane 10: E12.5+RCLG/Cre; lane 11: E13.5+RCLG; lane 12: E13.5+ RCLG/Cre; lane 13: E14.5+RCLG; lane 14: E14.5+ RCLG/Cre. The cropped gels are used in Supplementary Figure S20C, and the full-length gel images are available in Supplementary Figure S28. The gels have been run under the same experimental conditions.

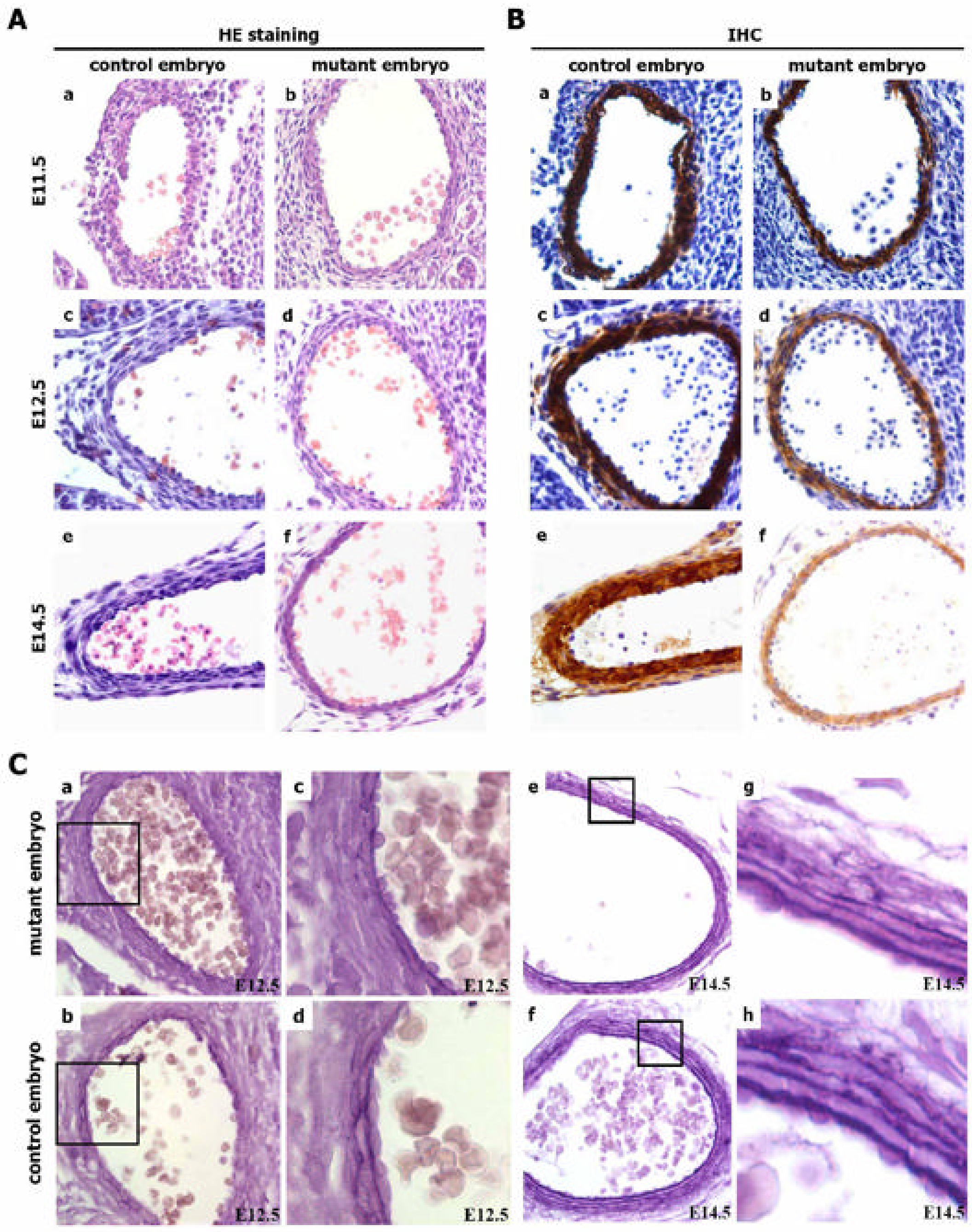

Figure S21. Mutant embryos (RCLG/EIIa-Cre) exhibit defects in the thoracic aorta.

(**A**) H&E staining of thoracic aorta sections from control and mutant embryos.

(**B**) IHC analyses of thoracic aorta sections stained with an anti-smooth muscle α-actin antibody.

(**C**) Gomori's aldehyde fuchsin staining of the elastic fibers of thoracic aorta in control and mutant embryos at E12.5 (a-d) and E14.5 (e-h).

(c), (d), (g) and (h) are higher magnifications of the regions indicated in (a), (b), (e) and (f), respectively.

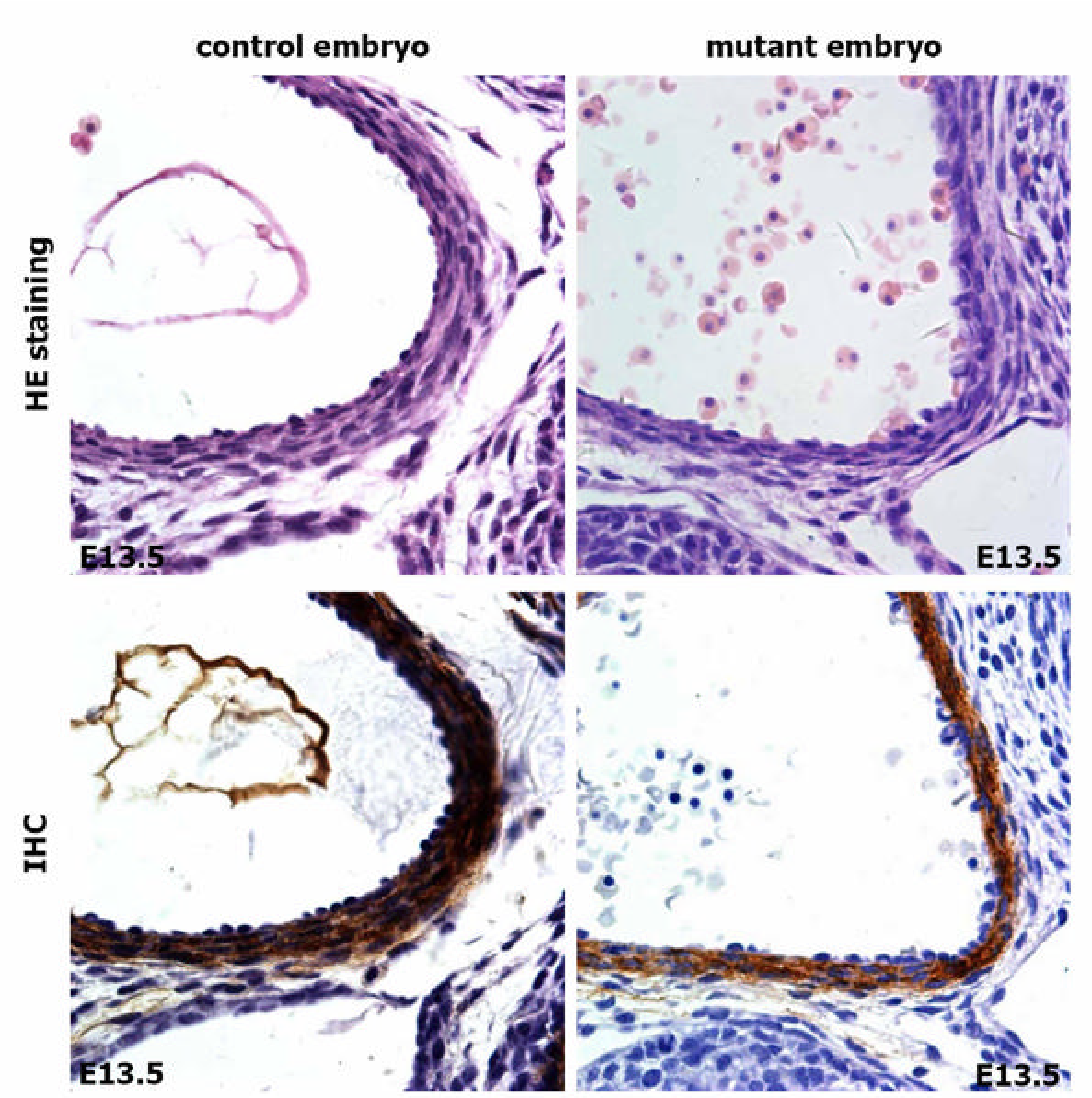

Figure S22. H&E staining and immunohistochemical (IHC) staining (for smooth muscle α-actin) of thoracic aorta sections from E13.5 control (EIIa-Cre) and mutant (RCLG/EIIa-Cre) embryos.

Thoracic aorta sections were stained with an anti-smooth muscle α-actin antibody to demonstrate smooth muscle cell layers of thoracic aorta.

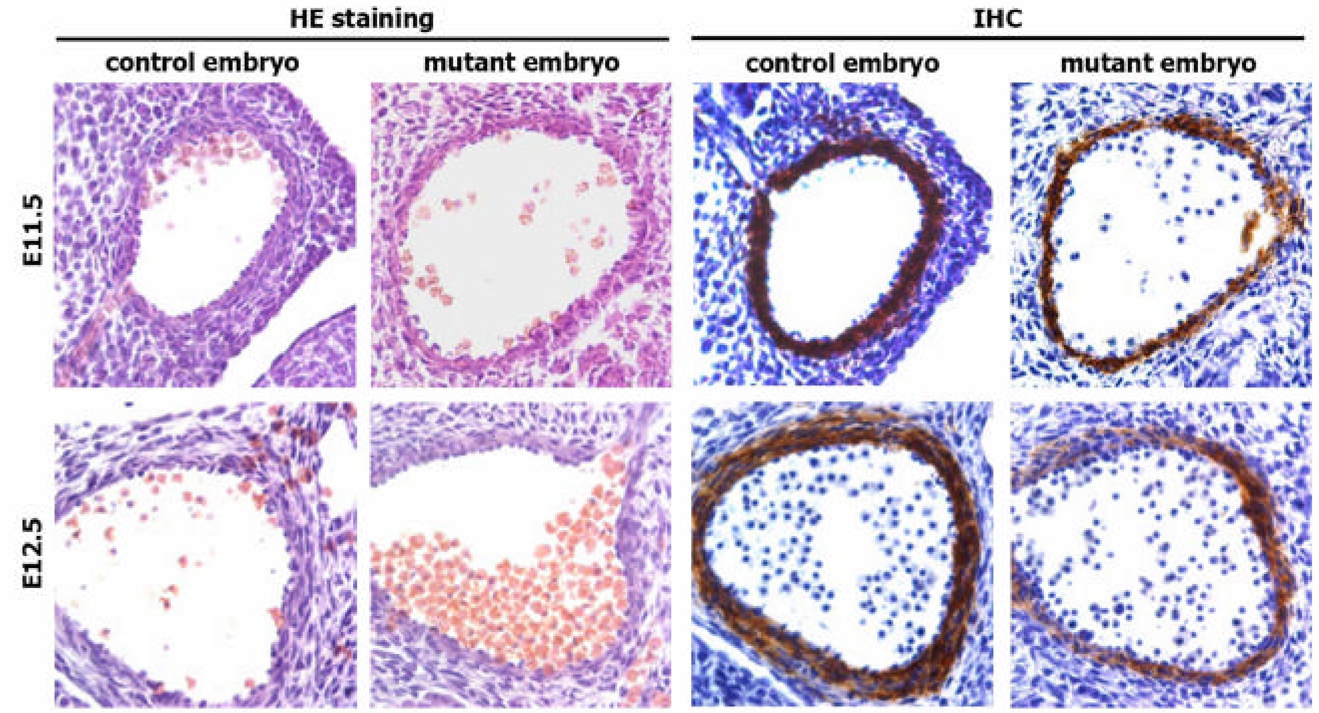

Figure S23. H&E staining and immunohistochemical (IHC) staining (for smooth muscle α-actin) of abdominal aorta sections from control (EIIa-Cre) and mutant (RCLG/EIIa-Cre) embryos at E11.5 and E12.5.

Abdominal aorta sections were stained with an anti-smooth muscle α-actin antibody to demonstrate smooth muscle cell layers of abdominal aorta.

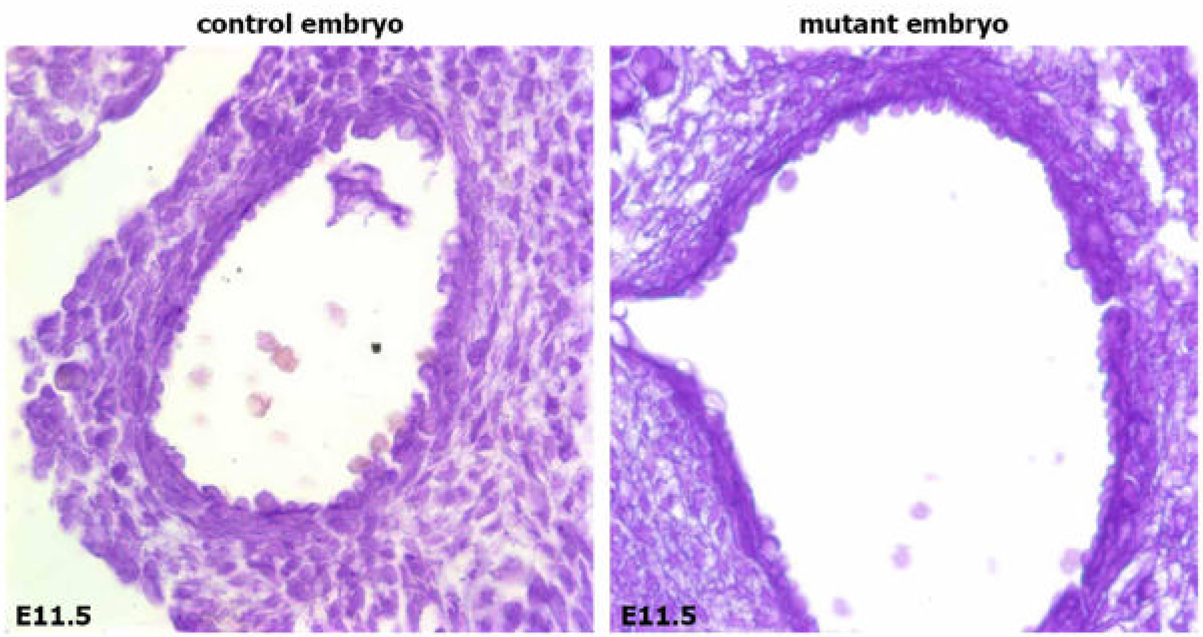

Figure S24. **Gomori's aldehyde fuchsin staining of elastic fibers of thoracic aorta in the control (EIIa-Cre) and mutant (RCLG/EIIa-Cre) embryos at E11.5.**

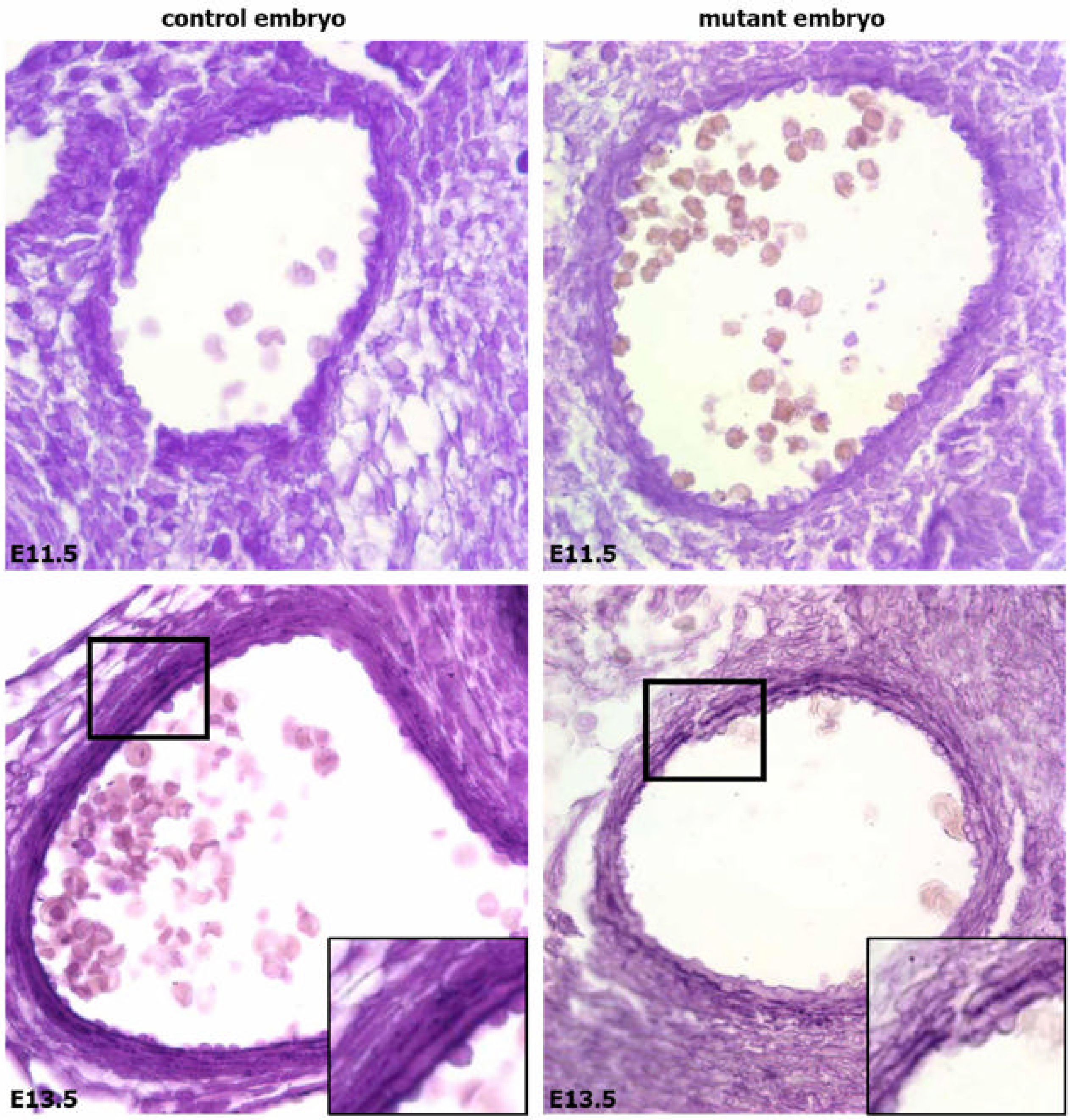

Figure S25. **Gomori's aldehyde fuchsin staining of elastic fibers of abdominal aorta in control (EIIa-Cre) and mutant (RCLG/EIIa-Cre) embryos at E11.5 and E13.5.**

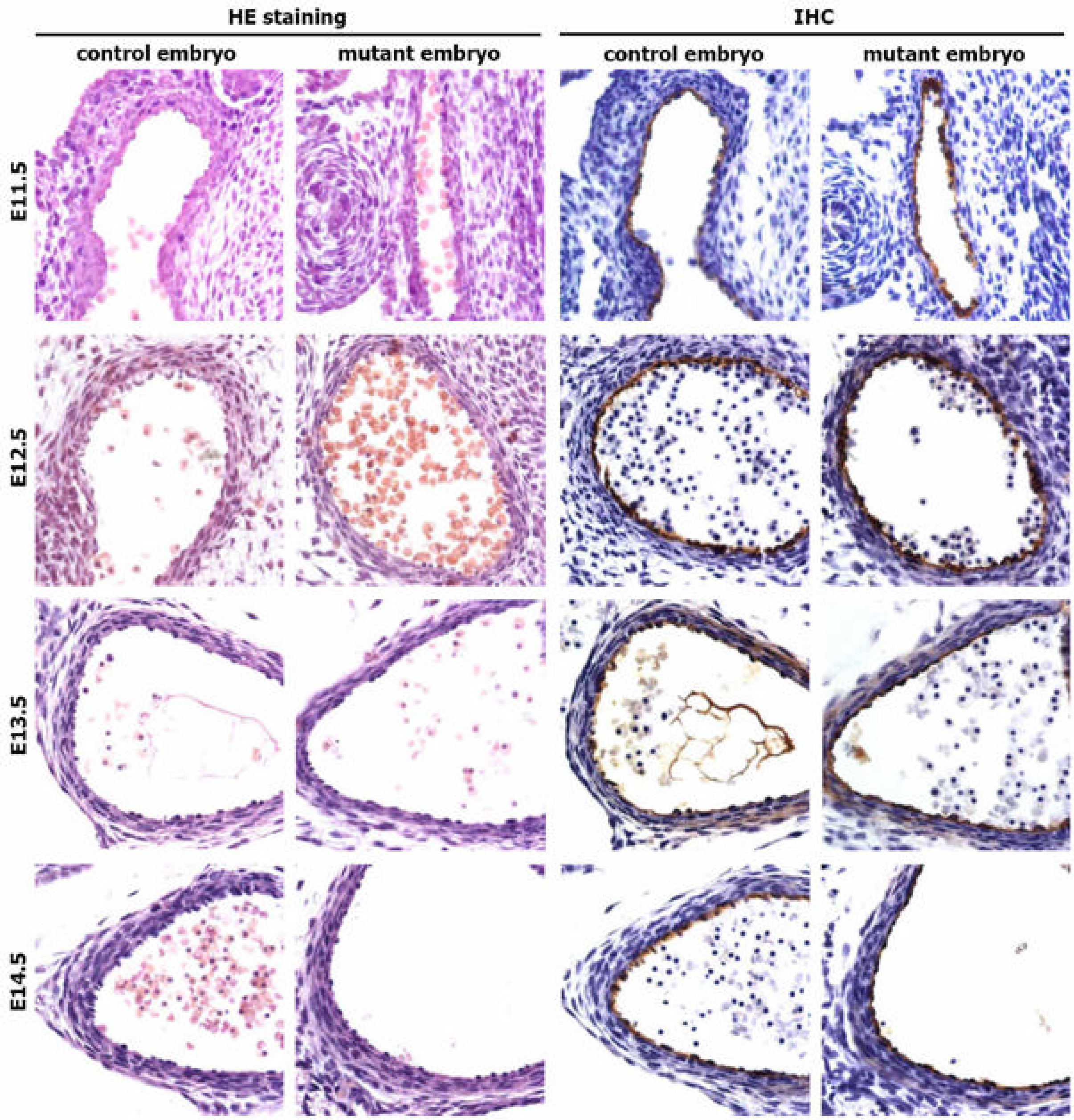

Figure S26. H&E staining and immunohistochemical (IHC) staining (for CD34) of thoracic aorta sections from control (EIIa-Cre) and mutant (RCLG/EIIa-Cre) embryos at E11.5 to E14.5.

Thoracic aorta sections were stained with an anti-CD34 antibody to reveal endothelial cell layer of thoracic aorta. Note the presence of endothelial cells in both control and mutant aortae.

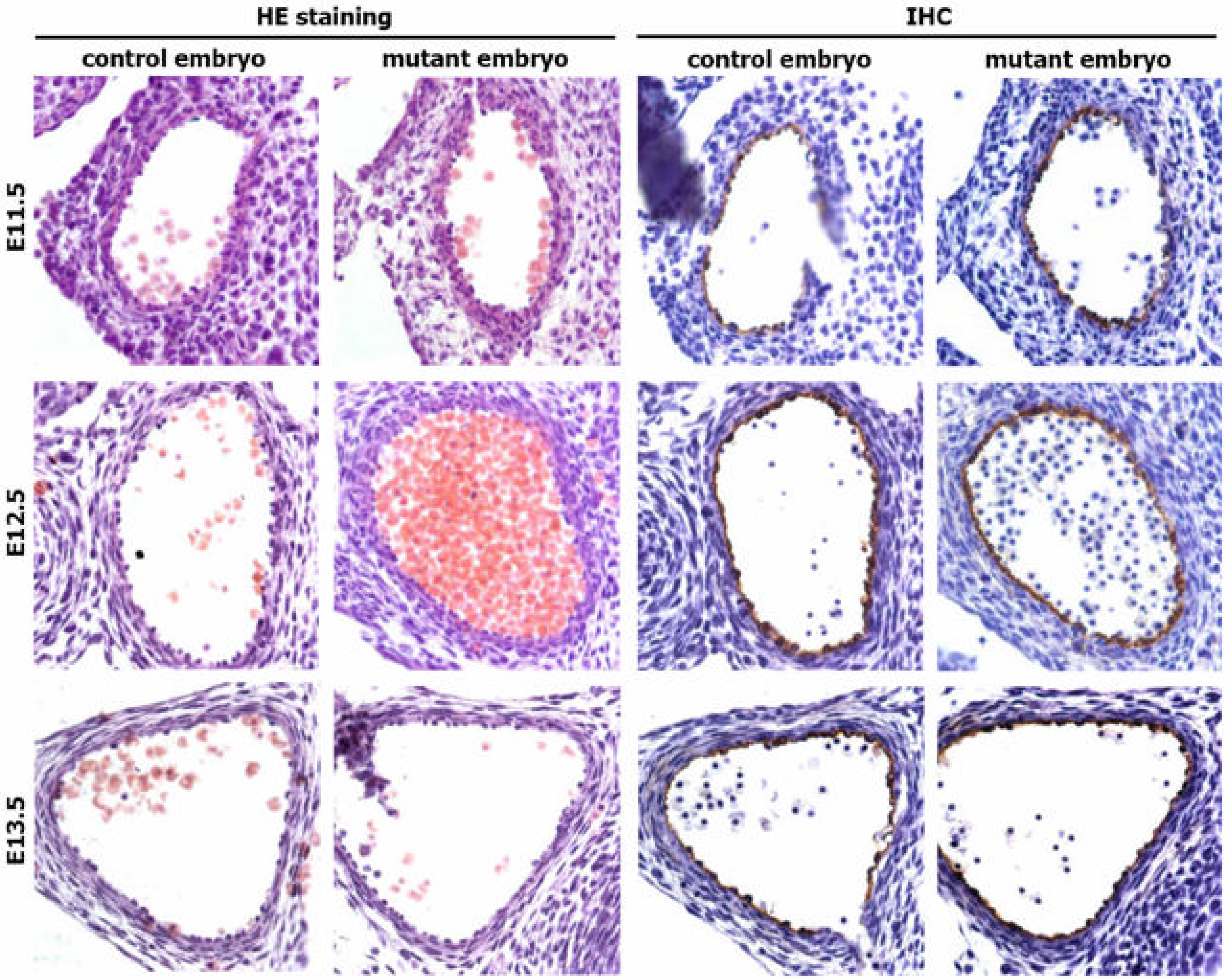

Figure S27. H&E staining and immunohistochemical (IHC) staining (for CD34) of abdominal aorta sections from control (EIIa-Cre) and mutant (RCLG/EIIa-Cre) embryos at E11.5, E12.5 and E13.5.

Abdominal aorta sections were stained with an anti-CD34 antibody to reveal endothelial cell layer of abdominal aorta. Note the presence of endothelial cells in both control and mutant aortae.

Supplementary Tables

Table S1 Primers for RT-PCR analysis

Gene	Forward primer (5′-3′)	Reverse primer (5′-3′)
α-SMA	CCCTGAAGAGCATCCGACA	CCAGAGTCCAGCACAATACC
CNN1	CGCATCGGGAACAACTTCATGTAT	CCCGGCTGCAGCTTGTTGATAAAT
GATA4	TCAAACCAGAAAACGGAAGC	GTGGCATTGCTGGAGTTACC
MYCD	AGGAAGTTCCGATCAGTCTTACA	GGTATTAAGCCTTGGTTAGCCAG
SM22A	ACCGTGGAGATCCCAACTGGTTTA	CATTTGAAGGCCAATGACGTGCT
SRF	CCAGGTGTCGGAATCTGACAG	GCTGACTTGCATGGTGGTAGA
GAPGH	AGGTCGGTGTGAACGGATTTG	GGGGTCGTTGATGGCAACA
hCripto-1	CACGATGTGCGCAAAGAGA	TGTAAATGCTGGCACGGTCA

Table S2 List of antibodies and suppliers used for immunoblotting

Antibody	Isotype	Dilution	Suppliers
Cripto-1	Rabbit IgG	1:1000	Abcam
GATA4	Rabbit IgG	1:1000	Abcam
ANP	Rabbit IgG	1:500	Bioss
MEF2C	Rabbit IgG	1:500	Bioss
MYH7B	Rabbit IgG	1:500	Bioss
α-SMA	Mouse IgG2a	1:400	Boster
Nkx2.5	Rabbit IgG	1:400	Boster
Ncx1	Rabbit IgG	1:400	Boster
CD34	Rabbit IgG	1:300	Boster
P-smad2	Rabbit IgG	1:1000	Cell signaling
Smad2/3	Rabbit IgG	1:1000	Cell signaling
BrdU	Mouse IgG2a	1:50	GE Healthcare
SRF	Rabbit IgG	1:1000	Proteintech
GAPGH	Rabbit IgG	1:1000	Bioss

Table S3 Genotypes of newborn progeny from mating RCLG or RLG transgenic mice with EIIα-Cre mice

Paired date	Parents		Number of offspring	Expression	
	Mother ID	Father ID		mRFP	Luc
2010-01-05	225 / RCLG	218 / EIIa-Cre	4	—	—
2010-03-08	225 / RCLG	284 / EIIa-Cre	9	—	—
2010-03-13	190 / RCLG	284 / EIIa-Cre	11	—	—
2010-03-26	313 / EIIa-Cre	326 / RCLG	2	—	—
2010-04-02	225 / RCLG	220 / EIIa-Cre	7	—	—
2010-04-06	315 / EIIa-Cre	326 / RCLG	1	—	—
2010-04-16	313 / EIIa-Cre	326 / RCLG	8	—	—
2010-04-18	359 / EIIa-Cre	326 / RCLG	6	—	—
2010-11-06	788 / RCLG	956 / EIIa-Cre	4	—	—
2010-10-19	1779 / RCLG	947 / EIIa-Cre	2	—	—
2010-10-13	941 / RCLG	856 / EIIa-Cre	3	—	—
2010-10-18	811 / RCLG	956 / EIIa-Cre	2	—	—
2010-10-22	1167 / EIIa-Cre	787 / RCLG	5	—	—
2010-11-03	1237 / RCLG	856 / EIIa-Cre	4	—	—
			Total：68	0	0
2010-03-05	318、319 / EIIa-Cre	189 / RLG	7	5 "+"	5 "+"
2010-03-25	316 / EIIa-Cre	297 / RLG	3	3"+"	3 "+"
2010-04-16	360 / EIIa-Cre	288 / RLG	9	9 "+"	9 "+"

All of RCLG transgenic mice used in mating are heterozygous. RLG transgenic mice (see Supplementary Figure S2 for details) are heterozygous or homozygous. EIIa-Cre mice are homozygous. Genotypes determined by whole-animal (new-born) fluorescence and bioluminescence imaging was confirmed by PCR-based genotyping (Supplementary Figure S1C).

Supplementary movies

Movie S1 DIC video microscopy of control (left) and mutant (RCLG/EIIa-Cre) (right) embryonic hearts at E12.5.

Movie S2 DIC video microscopy of control (left) and mutant (RCLG/EIIa-Cre) (right) embryonic hearts at E13.5.

Movie S3 DIC video microscopy of control (left) and mutant (RCLG/EIIa-Cre) (right) embryonic hearts at E14.5.

Movie S4 DIC video microscopy of control (left) and mutant (RCLG/EIIa-Cre) (right) embryonic hearts at E15.5.

Movie S5 DIC video microscopy of control (left) and mutant (RCLG/EIIa-Cre) (right) embryonic hearts at E16.5.

Movie S6 DIC video microscopy of control (left) and mutant (RCLG/hUb-CreERT2) (right) embryonic hearts at E13.5.

Movie S7 DIC video microscopy of control (left) and mutant (RCLG/hUb-CreERT2) (right) embryonic hearts at E14.5.

Supplementary references

1. Zheng, L., Njauw, C. N. & Martins-Green, M. A hCXCR1 transgenic mouse model containing a conditional color-switching system for imaging of hCXCL8/IL-8 functions in vivo. *J Leukoc Biol* **82**, 1247-1256, doi:10.1189/jlb.0307141 (2007).
2. Zheng, L., Njauw, C. N. & Martins-Green, M. A one-plasmid conditional color-switching transgenic system for multimodal bioimaging. *Transgenic Res* **17**, 741-747, doi:10.1007/s11248-007-9160-5 (2008).
3. Hayashi, S. & McMahon, A. P. Efficient recombination in diverse tissues by a tamoxifen-inducible form of Cre: a tool for temporally regulated gene activation/inactivation in the mouse. *Developmental biology* **244**, 305-318, doi:10.1006/dbio.2002.0597 (2002).
4. Danielian, P. S., Muccino, D., Rowitch, D. H., Michael, S. K. & McMahon, A. P. Modification of gene activity in mouse embryos in utero by a tamoxifen-inducible form of Cre recombinase. *Curr Biol* **8**, 1323-1326 (1998).
5. Ruzankina, Y. *et al.* Deletion of the developmentally essential gene ATR in adult mice leads to age-related phenotypes and stem cell loss. *Cell Stem Cell* **1**, 113-126, doi:10.1016/j.stem.2007.03.002 (2007).

The uncropped full-length gels and blots are shown below:

Fig. 1F

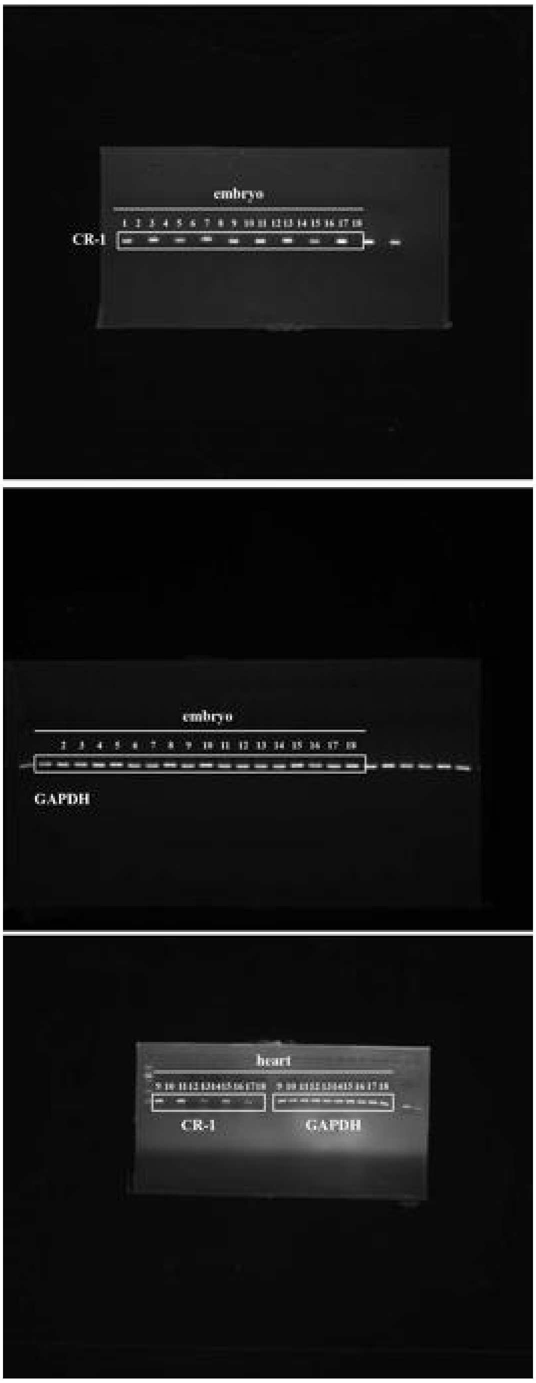

Fig. 1G

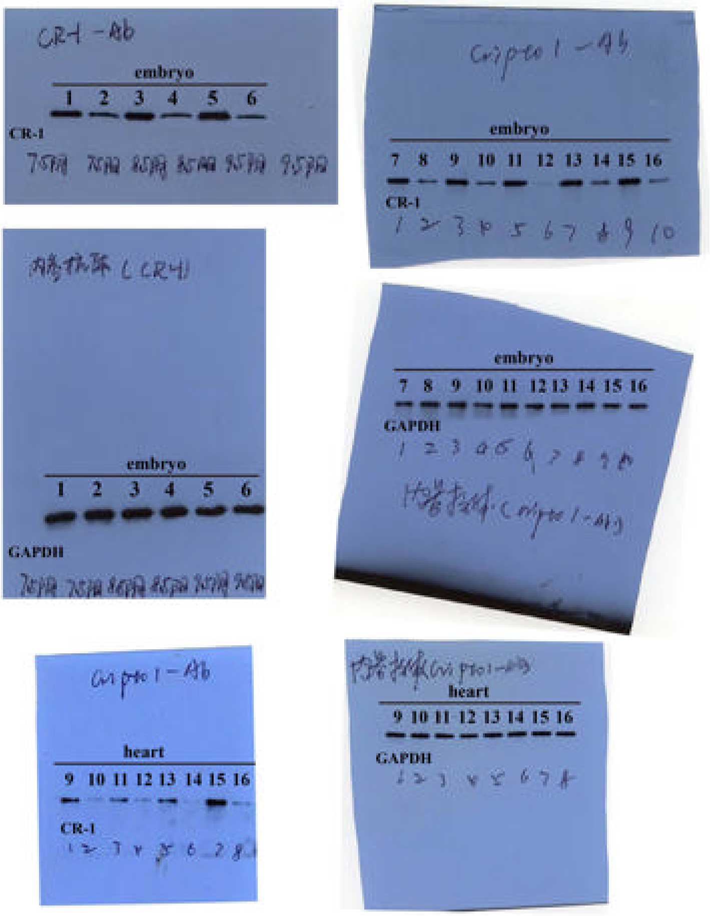

Figure S28 Full scans

Fig.6B

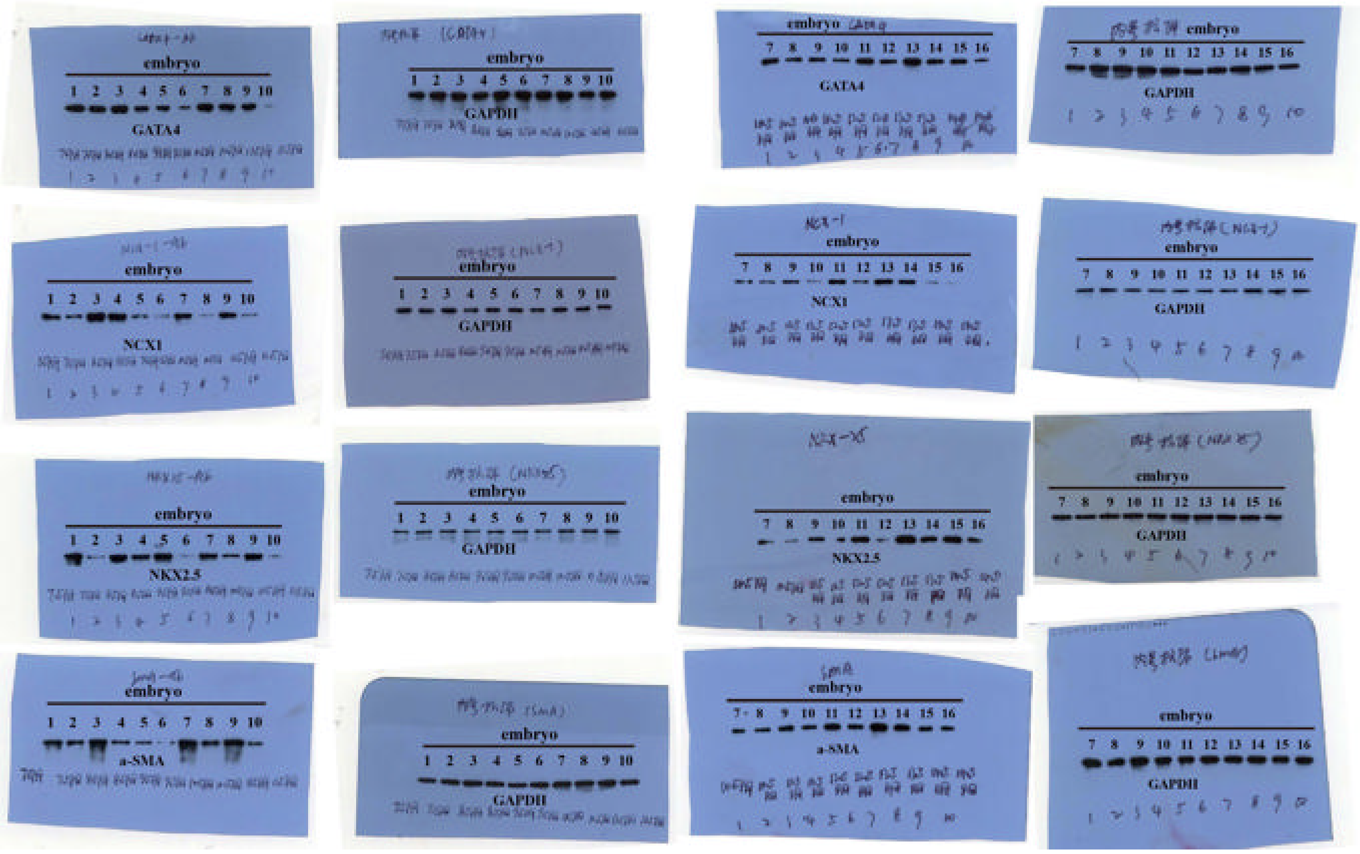

Fig.6C

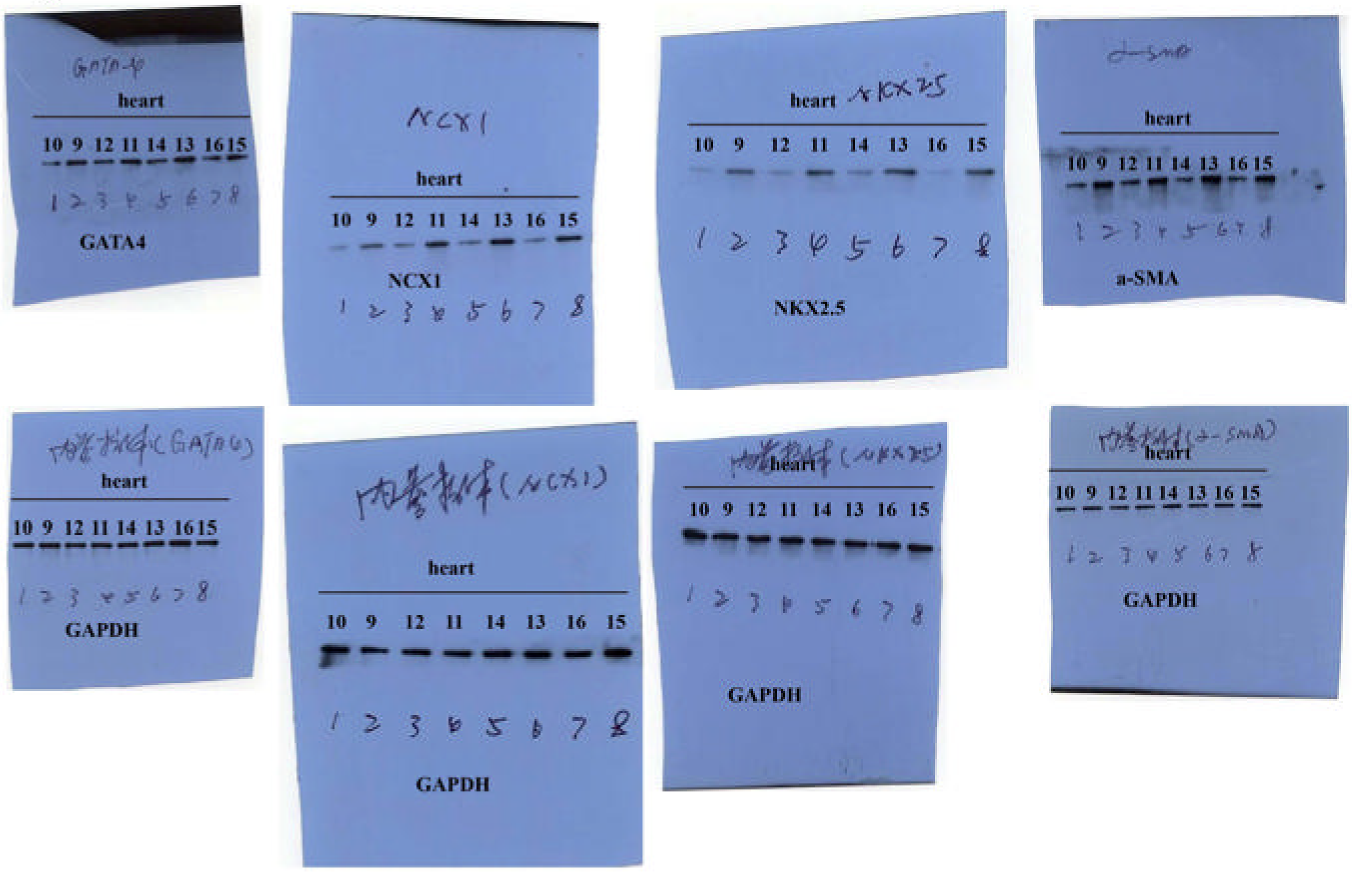

Figure S28 continued

Fig. 6D

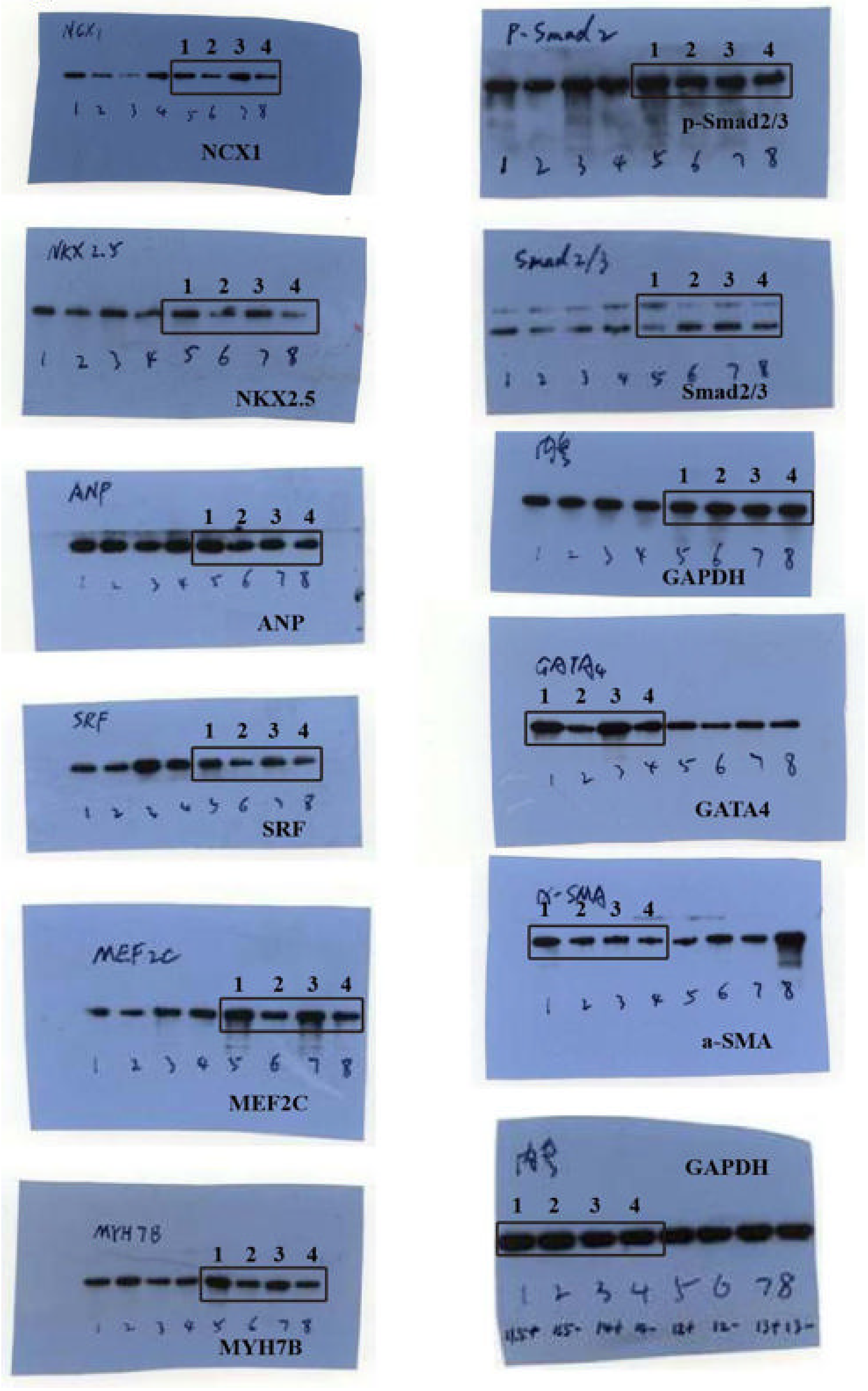

Figure S28 continued

Supplementary Fig. S1C

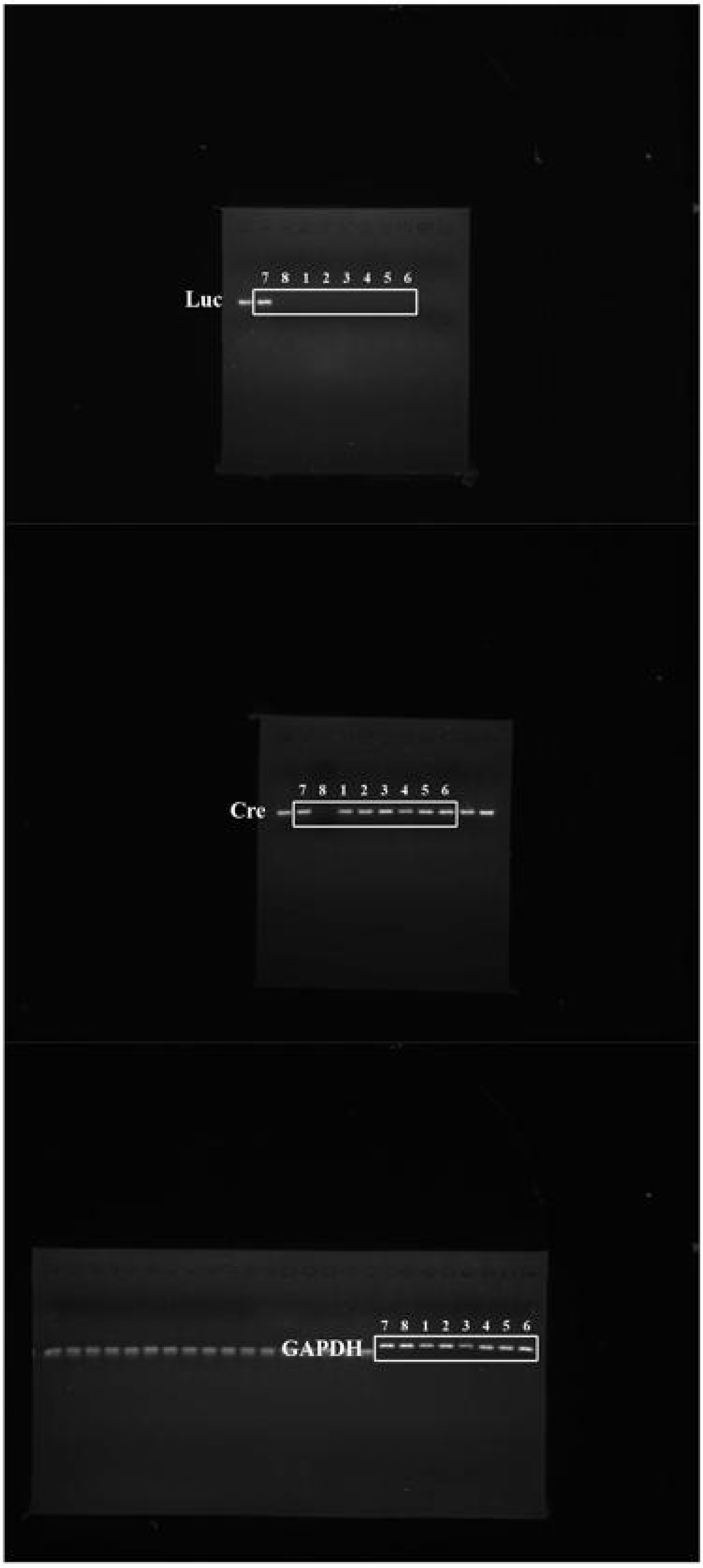

Figure S28 continued

Supplementary Fig. S20C

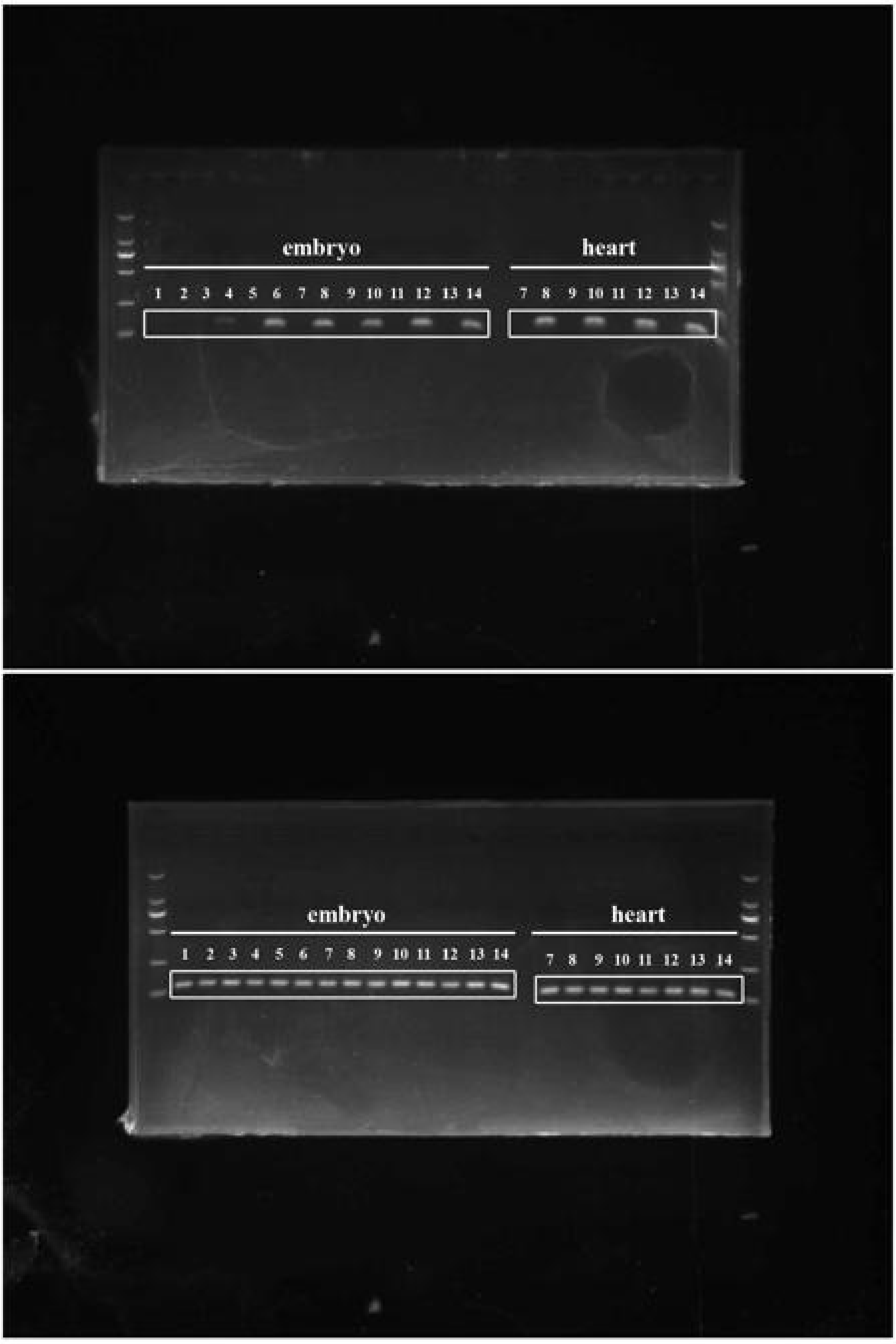

Figure S28 continued
